# Protein-driven RNA nanostructured devices that function in vitro and control mammalian cell fate

**DOI:** 10.1038/s41467-017-00459-x

**Published:** 2017-09-14

**Authors:** Tomonori Shibata, Yoshihiko Fujita, Hirohisa Ohno, Yuki Suzuki, Karin Hayashi, Kaoru R. Komatsu, Shunsuke Kawasaki, Kumi Hidaka, Shin Yonehara, Hiroshi Sugiyama, Masayuki Endo, Hirohide Saito

**Affiliations:** 1 Center for iPS Cell Research and Application, Kyoto University, 53 Kawahara-cho, Shogoin, Sakyo-ku, Kyoto 606-8507 Japan; 2 Department of Chemistry, Graduate School of Science, Kyoto University, Kitashirakawa Oiwake-cho, Sakyo-ku, Kyoto 606-8502 Japan; 3 Graduate School of Biostudies, Kyoto University, Kitashirakawa Oiwake-cho, Sakyo-ku, Kyoto 606-8502 Japan; 4 Institute for Integrated Cell-Material Sciences, Kyoto University, Yoshida-Ushinomiya-ch, Sakyo-ku, Kyoto 606-8501 Japan; 5 Present Address: Frontier Research Institute for Interdisciplinary Sciences, Tohoku University, Aramaki aza Aoba 6-3, Aoba-ku, Sendai 980-8578 Japan

## Abstract

Nucleic acid nanotechnology has great potential for future therapeutic applications. However, the construction of nanostructured devices that control cell fate by detecting and amplifying protein signals has remained a challenge. Here we design and build protein-driven RNA-nanostructured devices that actuate in vitro by RNA-binding-protein-inducible conformational change and regulate mammalian cell fate by RNA–protein interaction-mediated protein assembly. The conformation and function of the RNA nanostructures are dynamically controlled by RNA-binding protein signals. The protein-responsive RNA nanodevices are constructed inside cells using RNA-only delivery, which may provide a safe tool for building functional RNA–protein nanostructures. Moreover, the designed RNA scaffolds that control the assembly and oligomerization of apoptosis-regulatory proteins on a nanometre scale selectively kill target cells via specific RNA–protein interactions. These findings suggest that synthetic RNA nanodevices could function as molecular robots that detect signals and localize target proteins, induce RNA conformational changes, and programme mammalian cellular behaviour.

## Abstract (web summary)

Nucleic acid nanotechnology has great potential for future therapeutic applications. Here the authors build protein-driven RNA nanostructures that can function within mammalian cells and regulate the cell fate.

## Introduction

In the nucleic acid nanotechnology field, a variety of nanostructures have been designed and constructed to utilize the programmable features of nucleic acids and the defined size and periodicity of the double-helical structure^1, 2^. From this field, the concept of ‘nanomachine^3^’ or ‘molecular robots^4^’ has been investigated, because nucleic acids have the potential to change their conformations and functions based on the principle of simple Watson–Crick base pairing. For example, dynamic DNA nanostructures, such as the DNA walker^5^, the DNA motor^6^ and the DNA nanomachine^7–9^, have been constructed using DNA–DNA interactions. For biological applications, it is important to develop functional nanodevices that detect various environmental signals (e.g., RNA or protein signals), induce structural changes and produce desired functions (e.g., control mammalian cell fate). Several DNA nanostructures have been generated for potential biomedical and biotechnology applications, such as target cell-surface detection^10, 11^, imaging^12, 13^, drug delivery^14, 15^ and chemical reaction control^16^. For example, a DNA-based nanorobot has been designed to detect cancer cell-surface receptors and release a drug in target cells^10^. Stimuli-responsive DNA nanohydrogels with size-controllable properties^17^ and pH- or chloride-sensing DNA nanodevices have been constructed inside cells^18, 19^. In addition to DNA, RNA has attracted the attention of bioengineers because of the structural diversity of RNA molecules (i.e., structured RNA uses both canonical Watson–Crick base pairing and non-canonical RNA structural motifs to form various two-dimensional and three-dimensional (3D) structures)^20, 21^. Several RNA nanostructures, such as triangles, squares, nanorings, three-way junctions and prisms, have been constructed in vitro^22–35^ and some have been used for cellular applications through the attachment of a functional molecule, such as RNA (e.g., siRNA or aptamer)^25, 27, 28, 32^ or protein (e.g., cell-surface binder)^26, 27, 31–34^, on the designed RNA structures. Synthetic RNA scaffolds that control the assembly of enzymes for hydrogen production in bacteria have also been reported^26^. However, the construction of nanostructured devices that control mammalian cellular behaviour by detecting or accumulating intracellular protein signals has not yet been demonstrated.

Inside a cell, many RNA molecules cannot function alone. RNA molecules together with RNA-binding proteins construct nanostructured RNA–protein (RNP) complexes. For example, the ribosome, which is composed of ribosomal RNAs and proteins, is a nature-made, sophisticated RNP nanomachine that catalyses protein synthesis based on the information coded in genes. Clustered regularly interspaced short palindromic repeat-CRISPR-associated proteins (CRISPR-Cas9) are another example of RNP complex-mediated nanodevices that enable the editing of a target region of genomes in a customized manner^36^. Several long noncoding RNAs have been shown to function as natural scaffolds that can control the localization and function of chromatin regulatory proteins^37^. The naturally occurring RNP interactions often control a variety of biological functions through dynamic regulation of the structures and activities of intracellular RNA or protein. Thus, we considered building synthetic RNP nanostructured devices by mimicking natural RNP complexes that have the following properties: (1) RNA-nanostructured devices detect and localize target RNA-binding proteins both in vitro and inside cells; (2) the conformation of the RNA devices is dynamically changed through specific RNP interactions; and (3) the actuation of the RNA devices produces functional outputs dependent on the extracellular and intracellular environment.

**Fig. 1:**
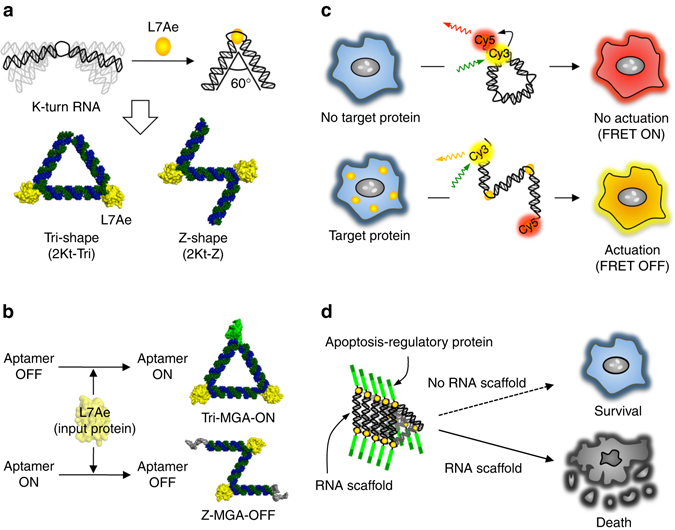
Schematic illustration of protein-driven RNA nanodevices in vitro and in mammalian cells. **a** Protein-triggered conformational change in RNA due to the L7Ae-K-turn interaction (*top*) and three-dimensional modeling of the 2Kt-Tri and 2Kt-Z nanostructures (*bottom*). **b** Protein-driven RNA nanodevices that control RNA function in response to proteins. **c** Actuation of protein-responsive RNA nanodevices that control fluorescence signals within cells. **d** Controlling apoptosis by regulating the assembly and oligomerization of apoptosis regulatory proteins on RNA scaffolds

Here we report protein-driven RNA nanostructured devices that function in vitro and within live mammalian cells. Specific RNP interactions induce both structural and functional changes in the RNA nanodevices. The actuated RNA devices produce various outputs, such as the activation and repression of RNA aptamers (Fig. 1a, b) and the detection of RNA-binding protein in cells (Fig. 1c). In addition, synthetic RNA scaffolds formed in mammalian cells can selectively control cell-death pathways by detecting endogenous RNA-binding protein or microRNA (miRNA) signals and regulating the assembly and oligomerization of apoptosis-regulatory proteins on a nanometre scale (Fig. 1d).

## Results

### Construction of protein-driven RNA nanodevices in vitro

To design protein-responsive RNA nanodevices that induce dynamic conformational change, we employed the box C/D kink-turn (K-turn) RNA and a small RNA-binding protein from archaea, called L7Ae, as an appropriate RNP interaction motif^38^. The K-turn adopts a flexible conformation in a protein-free state, whereas L7Ae strongly stabilizes the K-turn with an approximately 60° bending angle. We have previously designed triangle-like RNP nanostructures in vitro using three K-turn-L7Ae motifs inserted into three vertexes of nanostructures composed of double-stranded RNAs^31–33^. However, it has remained a challenge (1) to induce both dynamic conformational and functional changes in RNA-nanostructured devices in response to a protein signal and (2) to construct protein-driven, functional RNA nanostructures inside mammalian cells.

**Fig. 2:**
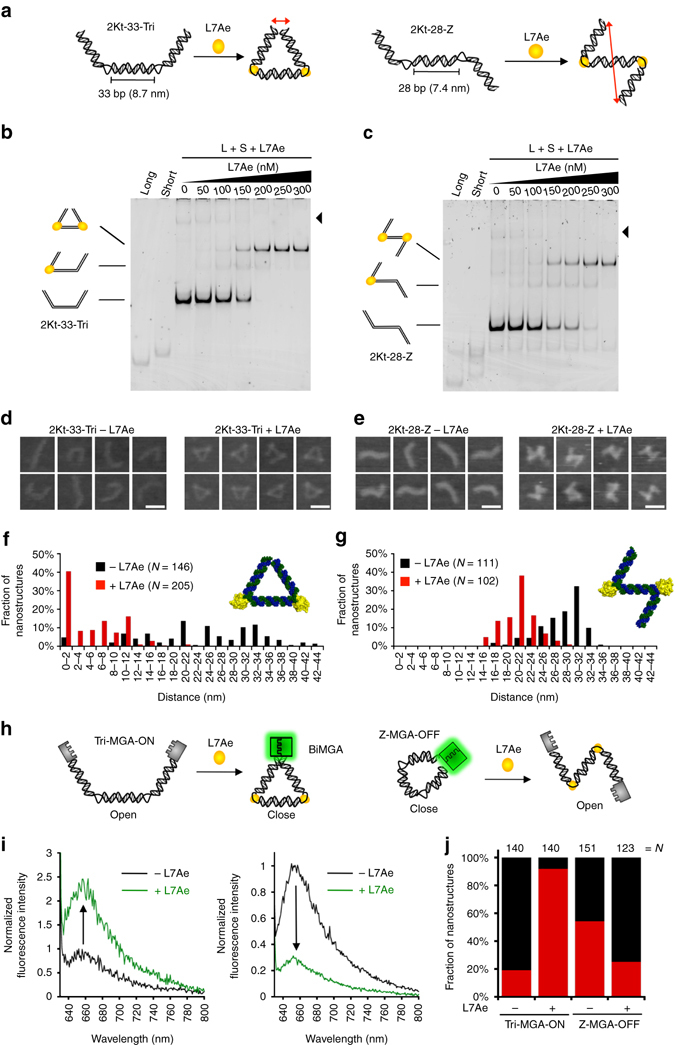
Actuation of protein-driven RNA nanodevices in vitro. **a** Schematic representation of structural changes in 2Kt-33-Tri (*left*) and 2Kt-28-Z (*right*) caused by the induced-fit of RNA in response to L7Ae binding. The distance between the two ends of the RNA is indicated by a red double-headed arrows. The in silico predicted lengths of each side of 2Kt-33-Tri and 2Kt-28-Z were ~8.7 nm and 7.4 nm, respectively. **b**, **c** EMSA confirmed the interaction between 2Kt-33-Tri (**b**) and 2Kt-28-Z (**c**) RNA nanostructures with L7Ae. Higher order bands (*black arrowheads*) indicate heterogeneous oligomers composed of L- and S-RNA strands. Concentrations of long and short RNAs: each 50 nM. **d**, **e** AFM images of the 2Kt-33-Tri (**d**) and 2Kt-28-Z (**e**) RNA nanostructures in the absence (*left*) and presence (*right*) of L7Ae. *Scale bars*, 20 nm. **f**, **g** Statistical distribution of the distance between the two ends of RNA, indicated by the double-headed arrows in **a**, for 2Kt-33-Tri (**f**) and 2Kt-28-Z RNA (**g**) nanostructures in the absence (*black*) and presence (*red*) of L7Ae (*N* number of nanostructures). **h** Schematic illustration of the ON/OFF switching of biMGA activity caused by structural changes in RNA nanodevices in response to L7Ae binding. Fluorescence emission of Tri-MGA-ON is caused by the formation of an active biMGA that occurs with a L7Ae-induced RNA conformational change that places two split aptamers close to each other (*left*). Fluorescence quenching of Z-MGA-OFF is caused by disassembly of the biMGA that occurs with a L7Ae-induced conformational change that separates the two split aptamers (*right*). **i** Fluorescence spectra of Tri-MGA-ON (*left*) and Z-MGA-OFF (*right*) in the absence (*black*) and presence (*green*) of L7Ae. **j** Fraction of open (*black*) and closed (*red*) nanostructures before and after RNP formation (*N* number of nanostructures). Tri-MGA-ON: Tri-MGA-ON-stem B (Supplementary Fig. 10). Z-MGA-OFF: Z-MGA-OFF-stem D (Supplementary Fig. 11)

To induce an apparent conformational change via the RNP interaction, we designed two RNA strands (long (L) and short (S) strands) in which Watson–Crick base pairs are formed within the strands but where the two ends of the double-stranded RNA do not interact with each other (Fig. 2a; protein-free double-stranded RNAs should form open, nearly linear structures). We designed two different types of RNP nanostructures that formed ‘triangle instrument’-like (Tri-shape; L7Ae bends two K-turn motifs to place the two ends of the double-stranded RNA close to each other) or ‘Z-shape’-like nanostructures (Z-shape; L7Ae bends two K-turn motifs in opposite directions; Figs 1a and 2a). To design the Tri-shape and Z-shape nanostructures, we incorporated two K-turn motifs into the 33 and 28 base pairs of 3 RNA duplexes corresponding to 3 and 2.5 (two and a half) helical turns, respectively (Supplementary Figs 1 and 2; 2Kt-33-Tri and 2Kt-28-Z). Through the L7Ae-induced conformational change, 2Kt-33-Tri and 2Kt-28-Z should form Tri-shape and Z-shape nanostructures. These structures enable nanometre-scale control of the distance between the RNA termini (Fig. 2a, *red arrows*).

We first examined the interaction of L7Ae with 2Kt-33-Tri and with 2Kt-28-Z using an electrophoretic mobility shift assay (EMSA; Fig. 2b, c). Both 2Kt-33-Tri and 2Kt-28-Z interacted with L7Ae in a concentration-dependent manner to give shifted bands, whereas defective K-turn (dKt)-containing RNA variants that were designed to be less capable of binding to L7Ae (2dKt-33-Tri and 2dKt-28-Z; Supplementary Figs 1 and 2)^38^ provided no retarded bands (Supplementary Fig. 3), suggesting that two L7Ae proteins bind to two K-turn motifs in 2Kt-33-Tri and 2Kt-28-Z. We next analysed the structural changes of 2Kt-33-Tri and 2Kt-28-Z in the absence and presence of L7Ae by atomic force microscopy (AFM; Fig. 2d, e). In the absence of L7Ae, 2Kt-33-Tri and 2Kt-28-Z adopted heterogeneous, nearly linear RNA structures due to the flexibility of RNA and the lack of interaction between the two edges of the two RNA strands. In contrast, in the presence of L7Ae, we observed a conformational change in 2Kt-33-Tri and 2Kt-28-Z: we found that the opened, nearly linear RNA structures were dynamically changed to Tri-shape- and Z-shape-like forms due to the two L7Ae-K-turn interactions on 2Kt-33-Tri and 2Kt-28-Z, respectively, which were observed at a resolution of a single RNP interaction by AFM (Fig. 2d, e and Supplementary Fig. 4). These results indicated that two L7Ae proteins bound to two K-turn motifs of the RNA nanostructures and induced the RNA conformational change, as expected.

To further analyse the structural change caused by the RNP interaction, we measured the distance between the two ends of 2Kt-33-Tri and 2Kt-28-Z (shown in *red arrows* in Fig. 2a) in the absence and presence of L7Ae (Fig. 2f, g). In the absence of L7Ae, the edge distance of 2Kt-33-Tri was widely distributed from 0 to 44 nm. In contrast, the addition of L7Ae narrowed the distribution of the distance (the major population was within 16 nm), because the two ends of 2Kt-33-Tri came close to each other due to the two L7Ae-K-turn interactions that bent the RNA at the two K-turn regions to give a Tri-like shape as the major population (Fig. 2f). Similarly, we observed a distribution change in the distance between the two ends of 2Kt-28-Z in the absence and presence of L7Ae (the average distance was ~21 nm; Fig. 2g), which was consistent with the expected distance from the 3D modelling of 2Kt-28-Z (~18 nm in the presence of L7Ae).

Conformational changes that regulate the distance between the two ends of structured RNAs support our concept, i.e., that we can control the distance and arrangement of RNA-conjugated molecules on the two edges through protein sensing as an input signal. To confirm our idea, we attached fluorescent Cy3 and Cy5 molecules to the edge of 2Kt-33-Tri and analysed the L7Ae-induced RNA conformational change using Förster resonance energy transfer (FRET; Supplementary Figs 5 and 6). We observed an increased fluorescence signal (*F*_665_/*F*_565_) of 2Kt-33-Tri in the presence of L7Ae, but we did not observe this signal for the defective RNA, 2dKt-33-Tri, suggesting that FRET occurred likely due to the structural transition of 2Kt-33-Tri induced by the L7Ae-K-turn interaction.

### ON/OFF switching of RNA function by changing the RNA shapes

Next, we aimed to control the function of the RNA aptamer (i.e., ON and OFF) using the L7Ae-induced RNA structural change. We chose the split RNA aptamer (binary malachite green aptamer, biMGA)^39^ and conjugated each split sequence to the 5′- and 3′-termini of the S-strand in 2Kt-33-Tri or 2Kt-28-Z (Supplementary Figs 7 and 8). In the presence of L7Ae, activated (ON) and repressed (OFF) states of the aptamer should be formed by the assembly and disassembly of two split RNA fragments through the formation of Tri-shape and Z-shape RNPs, respectively (Fig. 2h).

To modulate the function of biMGA, we optimized the length of the linker region between the RNA devices and biMGA, and the stem sequence of biMGA (Supplementary Figs 9–15 and Supplementary Note 1). We analysed the function of biMGA on the designed RNA nanostructures with malachite green (MG) in the presence and absence of L7Ae by measuring fluorescence spectra. The interaction between L7Ae and the two optimized RNA devices (Tri-MGA-ON and Z-MGA-OFF) controlled the formation of functional RNA aptamers in the ON state and OFF state, respectively (Fig. 2i). AFM analyses of Tri-MGA-ON and Z-MGA-OFF confirmed that the modulation of biMGA activity was indeed due to conformational changes in the designed RNA devices (Fig. 2j and Supplementary Fig. 16). Thus, we are able to control both the functions (the activation and repression of the aptamer) and structures (Tri-shape and Z-shape) of the RNA nanodevices through detection of the target protein signal.

### Actuation of protein-driven RNA nanodevices in living cells

**Fig. 3:**
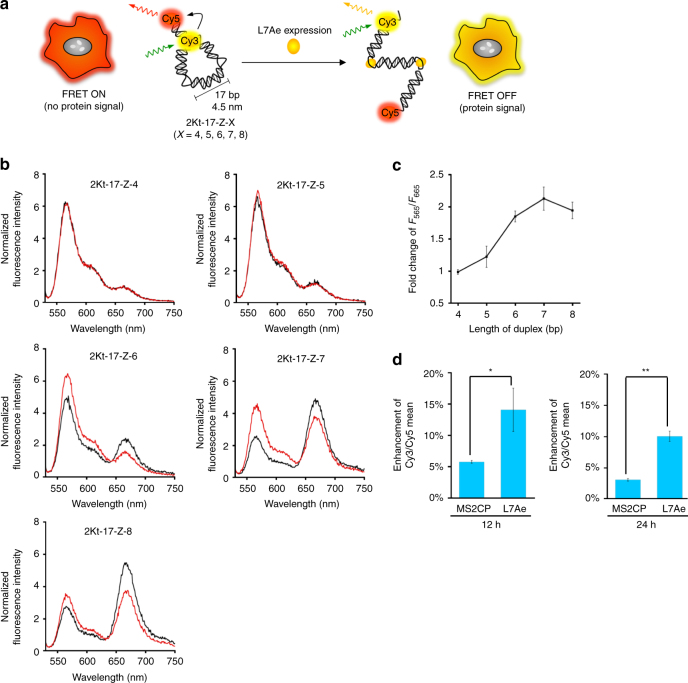
Actuation of protein-driven RNA nanodevice in vitro and inside live mammalian cells. **a** Schematic illustration of an intracellular RNA nanodevice that produces a change in the FRET signal upon detecting protein signals inside live mammalian cells. The in silico predicted lengths of each side of 2Kt-17-Z-X were ~4.5 nm. **b** Fluorescence spectra of 2Kt-17-Z-X (*X* = 4, 5, 6, 7, 8) in the absence (*black*) and presence (*red*) of L7Ae in vitro. **c** Plots of fold change in *F*_565_/*F*_665_, [*F*_565_/*F*_665_ (+ L7Ae)]/ [*F*_565_/*F*_665_ (− L7Ae)] versus the length of the RNA duplex. **d** Flow cytometric analysis of the actuation of the RNA nanodevice by a specific L7Ae-K-turn interaction within cells. MS2CP- or L7Ae-coding modRNA (500 ng) and 2Kt-17-Z-7 or 2dKt-17-Z-7 RNA nanodevices (8 pmol) were co-transfected into HeLa cells. Enhancement of the Cy3/Cy5 mean after 12 (*left*) and 24 (*right*) hours transfection was calculated from the Cy3/Cy5 mean intensities of cells transfected with 2Kt-17-Z-7 normalized to the Cy3/Cy5 mean intensity of cells transfected with 2dKt-17-Z-7. The data are presented as the mean ± SD (*n* = 3). *P < 0.05, **P < 0.01 (Welch’s *t*-test)

In cell-signalling pathways, proteins are central to processing information and controlling cell behaviour. We next aimed to construct and actuate protein-responsive RNA nanodevices within live mammalian cells: the RNA nanostructures should induce a conformational change when detecting a protein expressed in target cells. We thought that our in vitro designed Z-shape nanostructures could be used for protein-detector devices that output fluorescence signals through a specific RNP interaction-mediated conformational change in cells (Fig. 3a).

An RNA device with a Cy3- and Cy5-labelled short RNA duplex at the termini should produce an increase in the Cy3 signal at 565 nm (*F*_565_) and a decrease in the Cy5 signal at 665 nm (*F*_665_) by preventing FRET due to the formation of Z-shape RNP nanostructures. Thus, we first optimized the sequence of the short RNA duplex at the ends of the RNA devices in vitro by measuring the change in the *F*_565_/*F*_665_ value before and after the RNP formation. We engineered 2Kt-17-Z by introducing Watson–Crick base pairs (from 4 to 8 bp) at the 5′- and 3′-ends of the RNA device (2Kt-17-Z-4, 5, 6, 7 and 8; Fig. 3a and Supplementary Fig. 17) and measured the fluorescence spectra of the RNA variants labelled with Cy3 and Cy5 in the presence and absence of L7Ae (Fig. 3b). The fluorescence spectra of 2Kt-17-Z-4 and 2Kt-17-Z-5 showed few changes in the presence of L7Ae. In contrast, for 2Kt-17-Z-6, 2Kt-17-Z-7 and 2Kt-17-Z-8, *F*_565_ increased and *F*_665_ decreased simultaneously due to the RNP formation. We found the highest fold change in the *F*_565_/*F*_665_ values before and after the RNP formation when employing 2Kt-17-Z-7 (Fig. 3c). To check size effects of the RNA nanostructures, we also constructed larger Z-shape nanostructures, 2Kt-28-Z, with short RNA duplexes (from 5 to 7 bp) and confirmed that changes in the fluorescence signal were similar to 2Kt-17-Z (Supplementary Figs 18 and 19). Thus, we chose 2Kt-17-Z-7 for the following cellular experiments.

To deliver and construct protein-responsive RNA nanostructured devices inside mammalian cells, we chose the synthetic RNA delivery approach. This approach may provide a safe tool to manipulate cells without random genomic integration^40^, and in vitro transcribed RNAs (mRNA that codes a targt protein and small RNAs to make RNA devices) are directly used to construct RNP nanostructures in cells. Synthetic RNA devices (2Kt-17-Z-7) and chemically modified mRNA (modRNA; the use of modRNA was expected to reduce the interferon response)^40^ coding for L7Ae were co-transfected into HeLa cells. The expression of L7Ae was confirmed by capillary-based immunodetection (Supplementary Fig. 20). To detect the structural change in RNA nanodevices inside cells, we performed a flow cytometry-based FRET assay^41^ using Cy3- and Cy5-labelled RNA nanodevices (Fig. 3a and Supplementary Fig. 21). After 12 and 24 h of RNA transfection, we found that the Cy3/Cy5 signal of 2Kt-17-Z-7 increased in the presence of L7Ae compared with the signal in the presence of noncognate MS2CP protein or a defective RNA device (2dKt-17-Z-7) (Fig. 3d and Supplementary Fig. 21d–g). Although the enhancement of the signal in the cell was less than that observed in vitro, these results indicated that the increased Cy3/Cy5 signal of 2Kt-17-Z-7 was likely due to the conformational change of the intracellular RNA nanostructure caused by specific L7Ae-K-turn interactions. Thus, the protein signal could actuate the RNA nanostructured device in cell.

### RNA scaffold-based cell-fate control by assembling proteins

We next aimed to control cell fate (i.e., selective cell killing) by detecting and assembling an apoptosis regulatory protein, caspase-8 (Casp-8), through specific RNP interactions on designed RNA scaffolds formed in target cells. In naturally occurring systems, Casp-8 contains the death effector domain (DED), which is responsible for the assembly and activation of Casp-8 molecules through proximity-induced oligomerization^42^. We replaced DED with L7Ae, expecting that the fused L7Ae should bring multiple Casp-8 catalytic domains together on the RNA scaffold to induce caspase activation without requirement of the DED-mediated Casp-8 chain assembly. We therefore fused L7Ae with DED-defective Casp-8 (dCasp-8) and examined whether the RNP interaction-mediated accumulation of L7Ae-dCasp-8 on the RNA scaffold induces cell death (Fig. 1d). To control the assembly of L7Ae-dCasp-8 on the RNA scaffold, we newly designed a set of RNA scaffolds that contained 1, 3, 6, 9 or 14 K-turn motifs (1Kt-33-Tri, 3Kt-33-Tri, 6Kt-33-Tri, 9Kt-33-Tri or 14Kt-33-Tri, respectively), which would collect a different number of L7Ae-dCasp-8 molecules on the RNA scaffold at nanometre scale (Supplementary Fig. 22).

**Fig. 4:**
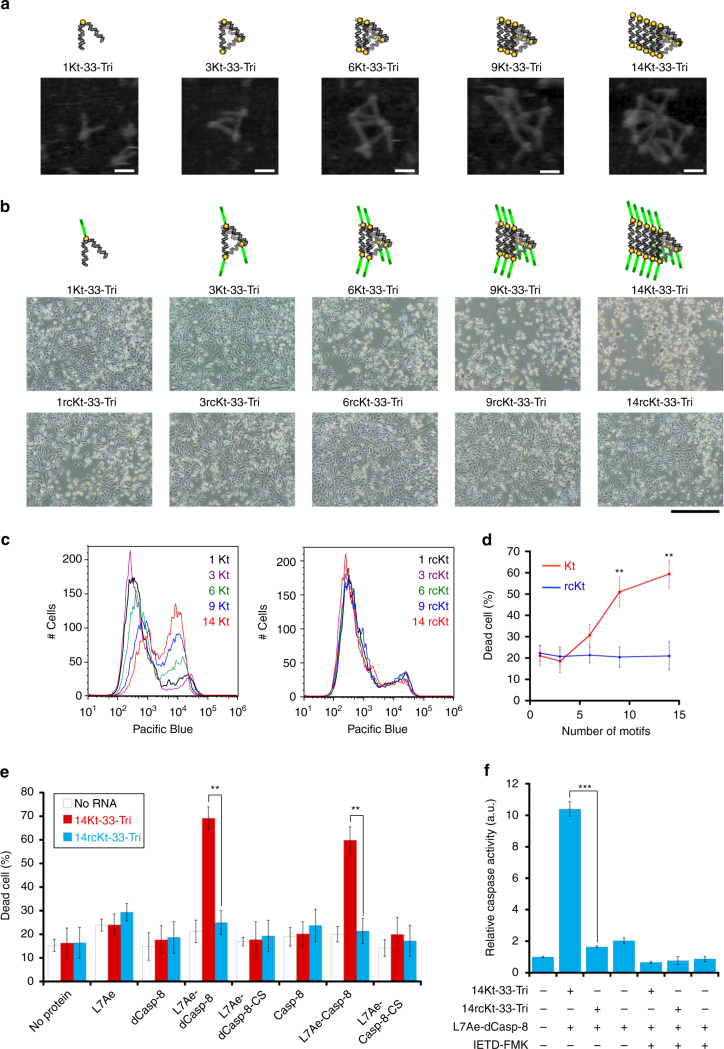
Control of mammalian cell fate by the assembly of apoptosis regulatory proteins on synthetic RNA scaffolds. **a** AFM images of 1, 3, 6, 9 and 14Kt-33-Tri RNA nanostructures in the presence of L7Ae. *Scale bars*, 10 nm. **b**, **c** Microscopic images (**b**) and flow cytometric analysis (**c**) of cells that contain RNA nanostructures and L7Ae-dCasp-8-coding modRNA. *Scale bars*, 500 µm. The cells were stained with Pacific Blue Annexin V and dead cell stain, and were analysed using flow cytometry. **d** Plots of the dead cell population versus the number of Kt (rcKt) motifs. Dead cells were counted by considering Pacific Blue-positive cells to be dead. **e** Selective cell death by a specific RNP interaction between 14Kt-33-Tri and L7Ae-dCasp-8 (or L7Ae-Casp-8). RNA nanostructures and modRNAs were co-transfected into HeLa cells. Cells stained with Pacific Blue Annexin V were counted as dead cells. **f** Caspase fluorometric assay for the detection of caspase activity. The caspase activity of HeLa cells transfected with RNA devices and modRNAs was measured using a fluorogenic substrate IETD-AMC. IETD-FMK was used as a caspase inhibitor. The data are presented as the mean ± SD (*n* = 3). **P < 0.01, ***P < 0.001 (Welch’s *t*-test)

The specific RNP interactions between 1-(3-, 6-, 9- or 14-)Kt-33-Tri and L7Ae were confirmed in vitro by EMSA and AFM (Fig. 4a and Supplementary Figs 23 and 24). In contrast, interactions between defective RNA scaffolds with reverse-complement K-turn motifs (rcKt; they do not interact with L7Ae; Supplementary Figs 23 and 25) and L7Ae were not. We found that the structures of the RNA scaffolds were clearly changed by the L7Ae addition (Supplementary Fig. 24). Furthermore, we directly observed that the number of L7Ae proteins assembled on the scaffolds increased as the number of K-turn motifs on the scaffolds increased (Fig. 4a). We confirmed L7Ae-dCasp-8 expression in cells by capillary-based immunodetection (Supplementary Fig. 26a). We also performed a translational repression assay^40^ and confirmed that the fusion of dCasp-8 to L7Ae maintained L7Ae-K-turn interactions in cells (Supplementary Fig. 26b, c). To investigate whether the designed RNA scaffolds could induce cell death through the accumulation of L7Ae-dCasp-8, each RNA device and L7Ae-dCasp-8-coding modRNA were co-transfected into HeLa cells and the cells were analysed by phase microscopy or flow cytometry after 24 h of transfection. Notably, we observed that 14Kt-33-Tri with L7Ae-dCasp-8 effectively induced cell death (Fig. 4b, *top right picture* and Fig. 4c, *left*), whereas the defective RNA scaffold (14rcKt-33-Tri) with L7Ae-dCasp-8 did not (Fig. 4b, *bottom right picture* and Fig. 4c, *right*). This result indicated that L7Ae-dCasp-8 alone does not induce cell death effectively, but the accumulation of L7Ae-dCasp-8 on the RNA devices facilitates cell-death induction. In addition, we observed that the dead cell populations increased as the number of Kt on the RNA scaffolds increased (9Kt and 14Kt), whereas the number of rcKt on the scaffolds did not affect cell death (Fig. 4c, d). These results suggest that we can tune cell-death signals by changing the number of dCasp-8 assembled on the designed RNA scaffold.

To further investigate whether cell death was induced by Casp-8-mediated apoptosis through specific RNP interactions, we constructed inactive variants of Casp-8 by replacing the catalytically active cysteine residue of Casp-8 with serine (Casp-8-CS)^43^ and transfected modRNAs encoding various protein variants (L7Ae, dCasp-8, L7Ae-dCasp-8, L7Ae-dCasp-8-CS, Casp-8, L7Ae-Casp-8 and L7Ae-Casp-8-CS) together with RNA scaffolds (14Kt-33-Tri or 14rcKt-33-Tri) (Fig. 4e and Supplementary Fig. 27). We confirmed that specific interactions between L7Ae-(d)Casp-8 and 14Kt-33-Tri are necessary for the induction of effective cell death, because L7Ae or (d)Casp-8 alone did not induce cell death even in the presence of 14Kt-33-Tri (Fig. 4e and Supplementary Fig. 27). Notably, cell death was not induced by full-length Casp-8 (or L7Ae-Casp-8) alone, whereas effective cell death was observed in 14Kt-33-Tri with L7Ae-Casp-8, indicating that the expression level of Casp-8 from modRNA is not enough to activate Casp-8 under our experimental condition, but 14Kt-33-Tri could increase the local concentration of L7Ae-Casp-8 to initiate cell death. This result suggests that RNA scaffolds may function as a signal amplifier capable of inducing cell death. In addition, we observed that 14Kt-33-Tri with L7Ae-(d)Casp-8-CS abolished the cell-death induction, indicating that Casp-8 activity is essential for triggering apoptosis mediated by the RNP interaction. We also measured caspase activity using a fluorogenic substrate (IETD-AMC). The highest caspase activity was observed in cells transfected with 14Kt-33-Tri and L7Ae-dCasp-8-coding modRNA, and the addition of a caspase inhibitor (IETD-FMK) abolished the observed activity (Fig. 4f). Together, these results confirm that we can selectively control cell-death signalling by regulating the oligomerization of pro-apoptosis regulatory proteins on synthetic RNA scaffolds formed in cells.

**Fig. 5:**
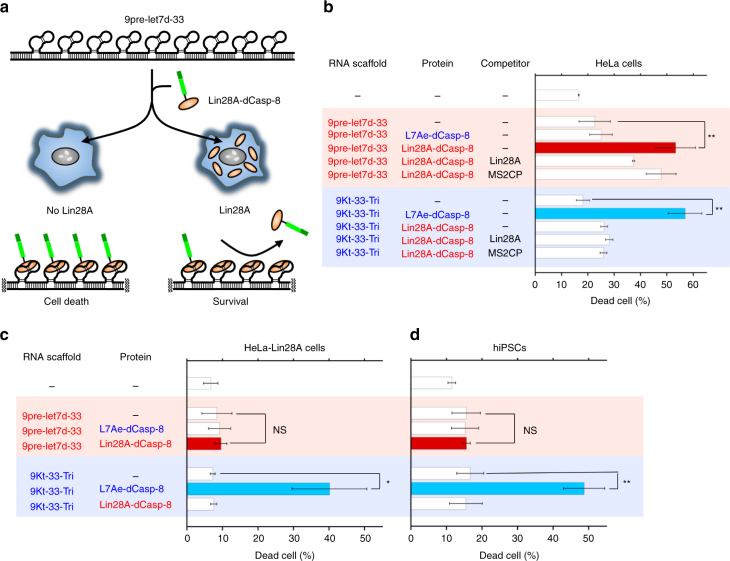
RNA scaffold-mediated cell-death control by Lin28A detection. **a** Schematic representation of a Lin28A-responsive RNA nanostructured device composed of 9pre-let7d-33 and Lin28A-dCasp-8. **b** Lin28A-responsive control of cell fate using 9pre-let7d-33 and Lin28A-dCasp-8 in HeLa cells. L7Ae-responsive 9Kt-33-Tri was used as a control. Cell death is induced in the presence of both 9pre-let7d-33 and Lin28A-dCasp-8. The induction of cell death is partially repressed by adding Lin28A-coding modRNA as a competitor. **c**, **d** Selective cell-fate control by detecting Lin28A in HeLa-Lin28A cells (**c**) and hiPSCs (**d**). Dead cells were stained with Pacific Blue Annexin V and analysed using flow cytometry. The data are presented as the mean ± SD (*n* = 3). NS not significant, *P < 0.05, **P < 0.01 (Welch’s *t*-test)

Next, we designed an alternative RNA scaffold to detect a different protein signal and control cell fate. Lin28A protein is a RNA-binding protein that regulates the biogenesis of several miRNAs (e.g., let-7d miRNA) and has important roles in cancer, tissue regeneration and the self-renewal of stem cells^44^. To develop a Lin28A-responsive cell-killing system with RNP nanostructures, we constructed Lin28A-fused dCasp-8 (Lin28A-dCasp-8) and a Lin28A-binding RNA scaffold, 9pre-let7d-33, by replacing the K-turn motif of 9Kt-33-Tri with pre-let-7d (RNA sequences derived from let-7d miRNA precursors), which interacts with Lin28A (Fig. 5 and Supplementary Fig. 28). We expected that the RNP interaction between Lin28A-dCasp-8 and 9pre-let7d-33 would induce cell death in a similar manner to L7Ae-dCasp-8 and 9Kt-33-Tri (Fig. 5a, *left*). Conversely, in cells that express Lin28A, the effect of cell death should be modulated by competitive binding between Lin28A and Lin28A-dCasp-8 to 9let7d-33, which should reduce the level of Casp-8 activation on the RNA scaffold (Fig. 5a, *right*). Transfection of 9pre-let7d-33 with Lin28A-dCasp-8-coding modRNA to HeLa cells showed effective cell death comparable to the effect of 9Kt-33-Tri with L7Ae-dCasp-8-coding modRNA, whereas Lin28A-dCasp-8 or 9pre-let7d-33 alone did not induce cell death, confirming that the RNP interaction is essential for triggering cell death. In addition, we observed that co-transfection of Lin28A-coding modRNA together with 9pre-let7d-33 and Lin28A-dCasp-8-coding modRNA reduced the number of dead cells (Fig. 5b), suggesting that Lin28A competes with Lin28A-dCasp-8 to bind 9pre-let7d-33. We also confirmed the expression of Lin28A and Lin28A-dCasp-8 in cells by capillary-based immunodetection (Supplementary Fig. 29).

To further investigate whether the system could selectively control cell fate by detecting endogenous expression levels of Lin28A, we constructed a stable HeLa-Lin28A cell line that expresses Lin28A from the genome (Supplementary Fig. 29). We compared Lin28A expression levels in HeLa-Lin28A and human induced pluripotent stem cells (hiPSCs, 201B7) that express endogenous Lin28A. We found that the Lin28A expression level in HeLa-Lin28A was lower than that in hiPSCs (Supplementary Fig. 30), suggesting HeLa-Lin28A cells do not express Lin28A beyond endogenous levels. We next transfected either 9pre-let7d-33 with Lin28A-dCasp-8-coding modRNA or 9Kt-33-Tri with L7Ae-dCasp-8-coding modRNA to HeLa-Lin28A cells (Fig. 5c). We observed that cells treated with 9pre-let7d-33 with Lin28A-dCasp-8 did not induce cell death, whereas cells treated with 9Kt-33-Tri with L7Ae-dCasp-8 increased the number of dead cells. These results indicate that cell-death signals initiated by 9pre-let7d-33 with Lin28A-dCasp-8 can be attenuated when Lin28A is detected in the cell. In addition, we transfected these RNA nanodevices in hiPSCs and found that Lin28A-dCasp-8 with 9pre-let7d-33 did not induce cell death, whereas L7Ae-dCasp-8 with 9Kt-33-Tri did in a manner similar to that in HeLa-Lin28A cells (Fig. 5d and Supplementary Fig. 31). Based on these results, we concluded that RNA-scaffold mediated cell-death control is adaptable to not only the L7Ae-K-turn interaction, but also the Lin28A-pre-let-7d interaction.

### Actuation of protein-driven RNA devices by miRNA signals

**Fig. 6:**
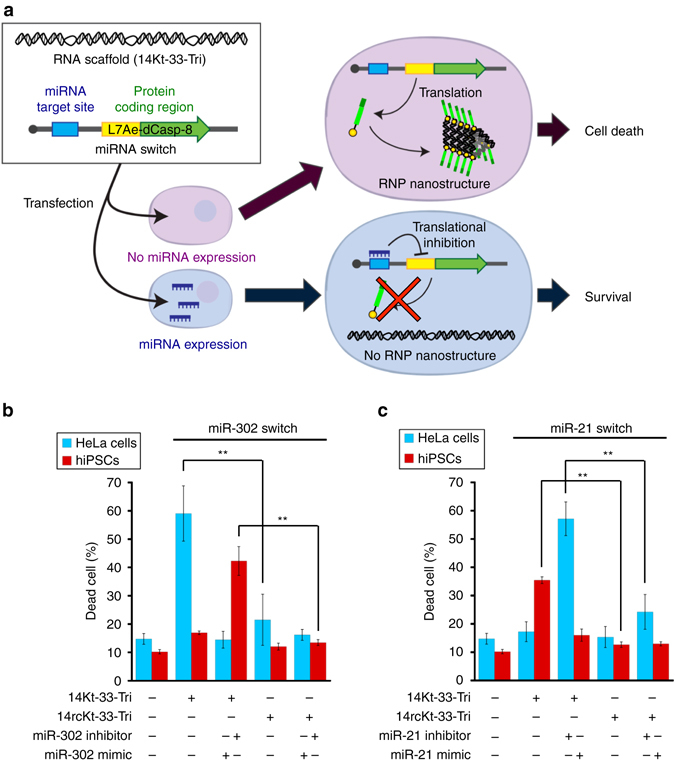
RNP nanodevices that detect endogenous miRNAs and selectively control cell-death signals. **a** Schematic representation of miRNA-responsive cell-death control mediated by miRNA switches encoding L7Ae-dCasp-8 and RNA scaffolds. **b**, **c** Cell-death induction using miR-302 (**b**) and miR-21 (**c**) responsive switches encoding L7Ae-dCasp-8 and 14Kt-33-Tri (14rcKt-33-Tri) in HeLa cells (*blue*) and hiPSCs (*red*). The dead cells were stained with Pacific Blue Annexin V and analysed using flow cytometry. The data are presented as the mean ± SD (*n* = 3). **P < 0.01 (Welch’s *t*-test)

Finally, in order to detect intracellular RNA signals and actuate protein-responsive RNA nanodevices, we constructed a system that responds to endogenous miRNAs and regulates Casp-8 protein signals. We designed synthetic mRNA^45^ that controls L7Ae-dCasp-8 expression in response to endogenous miRNAs expressed in target cells (miR-L7Ae-dCasp-8 switch). We linked this switch to an RNA device, 14Kt-33-Tri (Fig. 6a). To selectively control cell-death signals through miRNA detection, we applied our system to HeLa cells and hiPSCs, which express active miR-21-5p (miR-21) and miR-302a-5p (miR-302), respectively. Accordingly, we designed miR-21- and miR-302-responsive L7Ae-dCasp-8 mRNA switches. Transfection of miR-21 (miR-302)-L7Ae-dCasp-8 switch with the defective RNA scaffold (14rcKt-33-Tri) did not induce cell death in either cell type (Fig. 6b, c), indicating that miR-L7Ae-dCasp-8 switch without the correct RNA scaffold cannot trigger cell death. On the other hand, miR-302-L7Ae-dCasp-8 switch with 14Kt-33-Tri efficiently and selectively induced HeLa cell death but not hiPSC death (Fig. 6b) and miR-21-L7Ae-dCasp-8 switch with 14Kt-33-Tri selectively induced hiPSC death but not HeLa cell death (Fig. 6c). These results suggest that L7Ae-dCasp-8 protein assembly on 14Kt-33-Tri is essential to initiate cell death. In addition, we observed an increased level of dead cells by the addition of miR-302 or miR-21 inhibitors with the corresponding switches in hiPSCs and HeLa cells, respectively (Fig. 6b, c), indicating that endogenous miR-302 and miR-21 can control cell-death signals activated by the formation of RNP nanostructures. We also observed that the addition of a miR-302 or miR-21 mimic with the corresponding switches repressed the death of HeLa cells and hiPSCs, respectively (Fig. 6b, c). Together, these results suggest that we can control RNA scaffold-mediated cell death by detecting endogenous miRNAs and modulating protein signals in target cells.

## Discussion

In this study, we demonstrated the construction of protein-triggered, functional RNA nanostructured devices in vitro and within mammalian cells. Both the structures and the functions of the devices can be controlled via specific RNP interactions, which produce various output signals (i.e., ON/OFF switching of aptamer formation, intracellular protein-responsive actuation and cell-fate control by detecting intracellular protein or miRNA signals). Protein-responsive RNA-nanostructured devices may regulate various biological processes, because the spatiotemporal regulation of naturally occurring RNP complexes has a crucial role in the control of gene expressions, signal transductions and cell-fate conversions.

Our system provides a tool for controlling the localization and accumulation of signal proteins on RNA scaffolds that were designed on a nanometre scale. In fact, we found that the oligomerization of L7Ae-dCasp-8 proteins (i.e., more than nine dCasp8 proteins) on a designed RNA scaffold is important for the induction of effective cell death. In naturally occurring systems, the assembly and activation of Casp-8 are induced by the formation of death-inducing signalling complex (DISC), which is a key step in the death receptor-mediated activation of apoptotic pathways. Dickens et al.^46^ reported that DISC contains ninefold more Casp-8 than Fas-associated protein with death domain (FADD) and they proposed a DED chain assembly model for DISC formation. They demonstrated that the recruitment of many Casp-8 molecules to a single FADD molecule was required for DISC formation and subsequent apoptotic cell death. The DISC model seems to agree well with our observation that the assembly of more than nine L7Ae-dCasp-8 proteins on a single RNA scaffold facilitated effective Casp-8 activation and cell-death induction (Fig. 4). To further examine the effect of RNA structural configuration on cell-killing activity, we designed an alternative scaffold that formed ‘zigzag-shaped’ nanostructures (14Kt-27,28-Zig) by RNP interaction (Supplementary Fig. 32). The cell-killing activity of 14Kt-27,28-Zig was comparable to that of 14Kt-33-Tri (Supplementary Fig. 33), suggesting that the assembly and oligomerization of dCasp-8 proteins on the RNA scaffolds are important for cell-death induction.

The molecular design of RNA nanodevices can be applied to various RNA motifs that bind to target proteins, including naturally occurring motifs and in vitro selected aptamers. Indeed, we designed and constructed alternative RNA nanodevices that control their structures by responding to U1A protein by employing U1A-binding motifs^47^ (Supplementary Fig. 34). We observed that U1A protein interacted with the RNA device to induce RNA conformational change (Supplementary Fig. 35). To actuate RNA nanodevices in response to a target protein within mammalian cells, RNA-binding proteins of interest (e.g., Lin28A) could be fused to functional proteins (e.g., dCasp-8) and the protein-binding RNA motifs can be incorporated into the RNA nanodevices. We designed a Lin28A-resposive, cell-fate control system using the 9pre-let7d-33 nanodevice (Fig. 5). We found that Lin28A expressed in HeLa-Lin28A cells or hiPSCs inhibited Lin28A-dCasp-8-mediated cell killing activity, probably due to the competitive binding between Lin28A and Lin28A-dCasp-8 to 9pre-let7d-33. These data suggest that the RNA scaffold-mediated cell death system could be modular and controllable by detecting various protein signals.

To detect endogenous miRNAs and control protein signals on the RNA scaffold, we combined our protein-responsive RNA nanostructured devices with miRNA-responsive mRNAs (miR-switches)^45^. The combination of 14Kt-33-Tri and miR-L7Ae-dCasp-8 switch selectively induced cell death in HeLa cells or hiPSCs by responding to the endogenous miRNA signatures (miR-21 or miR-302) and by regulating Casp-8-mediated apoptosis signals on the RNA scaffold. Transfection of full-length Casp-8- or L7Ae-Casp-8-encoding mRNA did not induce cell death (Fig. 4e), indicating that simple use of miRNA switches that express Casp-8 cannot control the cell phenotype efficiently. In contrast, RNA scaffold 14Kt-33-Tri amplified cell-death signals produced from miR-L7Ae-dCasp-8 switch and increased the number of dead cells in the absence of the target miRNAs. We propose that the combination of miRNA switches and synthetic RNA scaffolds can facilitate the control of miRNA-responsive cell-signalling pathways for the following reasons: (1) the simple use of miRNA switches that encode cell-death inducers may cause leaky expression of the proteins even in the presence of miRNA, which could blur the threshold between different cell types, because the expression balance between miR-switches and miRNA is critical for controlling cell death; (2) synthetic RNA scaffolds that assemble and oligomerize target proteins at nanometer scale can amplify the effect of protein signals by increasing the local concentration of the proteins on the scaffold: signal amplification processes using RNA scaffolds facilitate to control cell fate; and (3) RNA scaffolds can tune the signals by changing the assembly number of the apoptosis regulatory proteins on the scaffold (Fig. 4a–d).

To develop functional nanodevices in mammalian cells for future therapeutic applications, the introduction and formation of nanostructures inside target cells should be safe and efficient. Thus, we thought that the formation of RNP nanostructures in cells by RNA-only delivery is ideal for the following reasons: (1) the gene delivery of synthetic RNA rather than DNA is more likely to avoid potential genomic damage caused by random genomic integration of devices^40^; (2) modRNA can produce RNA-binding proteins without an immune response^40^; (3) the function of RNA devices can be transiently actuated inside cells, reducing the risk of potential side effects; and (4) the self-folding of RNA strands in vitro before transfection may facilitate the efficient detection of intracellular proteins via RNP interactions in the cytoplasm and the subsequent control of cell functions, because neither transcription nor RNA export from the nucleus are required in the case of RNA delivery; (5) RNA delivery approaches may have diverse medical applications, such as RNA vaccines^48^, cell reprogramming^49^, cell purification^45^ and cell-fate control^40^.

The construction of protein-responsive nucleic acid nanodevices in vitro has been reported. Examples of such nanodevices include nanomechanical DNA origami that detects proteins at the single-molecule level^8^, DNA nanorobots that recognize the cell surface and control cell signalling by utilizing the interaction between DNA aptamers and protein^10^, and protein-triggered DNA nanomachines on gold nanoparticles that control the assembly and cleavage of DNA components^50^. However, the detection of proteins by DNA nanodevices has been limited to in vitro or cell surfaces. Protein-assembled RNA nanostructures have also been constructed in vitro^31–34^, although these RNA nanostructures had rather static scaffolds and their conformations and functions were not controlled by the protein signal. Our RNA nanostructured devices can be actuated *via* their conformational changes both in vitro and inside mammalian cells in response to extracellular and intracellular protein signals, respectively. In addition, our RNA devices are signal transducers that are capable of converting the input protein signal into the work of RNA (e.g., the formation of active aptamers) or protein (e.g., apoptosis induction) by employing a dynamic RNA scaffold. We believe that RNA and RNP nanodevices can be employed as a material to construct molecular robots that regulate their structures and produce desired functions by sensing various types of extracellular and intracellular information.

## Methods

### Plasmid construction

DNA templates of 14Kt-33-Tri-long and 14Kt-33-Tri-short and 14Kt-27,28-Zig-long and 14Kt-27,28-Zig-short with *Bam*HI and *Xba*I sites were purchased from Life Technology. Preparation of 9pre-let7d-33-long plasmid was delegated to FASMAC. The DNA templates were inserted into the *Bam*HI–*Xba*I sites of pcDNA4/TO to give the desired plasmids. We used pcDNA3.1-L7Ae-myc-His6 and pBS-mFLICE^51^ (or pCSII-EF-Flag-mCasp8 CS-IRES-GFP^43^) for the construction of L7Ae-dCasp-8 and L7Ae-Casp-8 (or L7Ae-dCasp-8-CS and L7Ae-Casp-8-CS) plasmids. The dCasp-8 and full length Casp-8 was amplified from pBS-mouse Casp-8 by PCR using the corresponding primer sets, digested with *Eco*RI and *Xho*I, and inserted into the *Eco*RI–*Xho*I site of pcDNA3.1-L7Ae-myc-His6 to give pcDNA3.1-L7Ae-dCasp-8-myc-His6, pcDNA3.1-L7Ae-Casp-8-myc-His6 and the corresponding CS mutants. Lin28A was amplified from pLIN28Amyc-T2A-tagRFP by PCR using Lin28A-Fwd and Lin28A-Rev, digested with *Eco*RI and *Not*I, and inserted into the *Eco*RI–*Not*I site of pcDNA3.1-L7Ae-dCasp-8-myc-His6 to give pcDNA3.1-Lin28A-dCasp-8-myc-His6.

### DNA and RNA preparation for constructing RNA nanostructures

DNA templates and primers were purchased from Greiner Japan. DNA templates for in vitro transcription were prepared by PCR using KOD-Plus-Neo DNA Polymerase (TOYOBO). The DNA templates were purified using a MinElute PCR Purification Kit (QIAGEN), according to the manufacturer’s protocol. RNAs were transcribed by a MEGAshortscript Kit (Ambion), according to the manufacturer’s protocols. The transcribed RNAs were purified by denaturing polyacrylamide gel electrophoresis (PAGE). After the recovery of the RNAs, the RNA concentration was measured using NanoDrop (Thermo Scientific). The DNA templates of 1Kt-33-Tri and 1rcKt-33-Tri were generated by PCR using synthetic oligo-DNAs. The DNA templates of 3, 6, 9 and 14Kt-33-Tri, and the corresponding mutants (3, 6, 9 and 14rcKt-33-Tri) were amplified from pcDNA4/TO-14Kt-33-Tri by PCR using the corresponding primer sets. The DNA templates were transcribed using a MEGAshortscript Kit (Ambion) and a modified protocol. Psuedouridine-5’-triphosphate and 5-methylcytidine-5’-triphosphate (TriLink BioTechnology) were used instead of uridine-5’-triphosphate and cytidine-5’-triphosphate. The transcription of 9pre-let7d-33 was conducted with only cytosine modification to maintain the RNP interaction between pre-let-7d and Lin28A. Reaction mixtures were incubated at 37 °C for 4–16 h. TURBO DNase (Ambion) was added to the reaction mixtures to remove template DNAs. After incubation at 37 °C for 30 min, the RNAs were purified using a FavorPrep Blood/Cultured Cells total RNA extraction column (Favorgen Biotech) and then treated with Antarctic Phosphatase (New England Biolabs). After incubation at 37 °C for 30 min, the resulting RNAs were purified by an RNeasy MinElute Cleanup Kit (QIAGEN) and denaturing PAGE.

### Preparation of DNA template for modRNA synthesis

The 5′- and 3′-untranslated regions (UTRs) were generated by using PCR with the proper sets of synthetic oligo-DNAs. The protein-coding regions of the desired proteins were prepared by PCR with the corresponding protein-containing plasmids using proper primer sets. The template plasmids were digested with the *Dpn*I restriction enzyme (TOYOBO) for 30 min at 37 °C. The PCR products were purified using a MinElute PCR Purification Kit (QIAGEN), according to the manufacturer’s protocol. DNA templates for modRNA synthesis were generated by PCR using the primer set T7FwdG3C (0.3 µM) and Rev120A (0.3 µM) and using the PCR products of the 5′-UTR PCR (10 nM), 3′-UTR PCR (10 nM), and a coding region (0.2 ng µl^−1^). DNA templates for enhanced green fluorescent protein (EGFP)-myc-coding modRNA synthesis were generated by PCR using the primer set CGCT7G2 (0.3 µM) and Rev120A (0.3 µM), the PCR products of 5′-UTR PCR (10 nM) and an EGFP coding region. DNA templates for miRNA-responsive modRNAs (miRNA switches) were prepared by using the PCR products of miRNA target site-containing 5′-UTR (5′-UTR-T21-5p PCR or 5′-UTR-T302a-5p PCR) instead of the above 5′-UTR PCR. The PCR products were purified with the above-mentioned protocol.

### Preparation of modRNAs

mRNAs were generated from the corresponding DNA templates using a MEGAscript T7 Kit (Ambion) and a modified protocol. Psuedouridine-5’-triphosphate and 5-methylcytidine-5’-triphosphate (TriLink BioTechnology) were used instead of uridine-5’-triphosphate and cytidine-5’-triphosphate. A mixture of Anti-Reverse Cap Analog (TriLink) and guanosine-5’-triphosphate (4:1) was used instead of guanosine-5’-triphosphate. Reaction mixtures were incubated at 37 °C for 4 h and then treated with TURBO DNase (Ambion) at 37 °C for 30 min. The RNAs were purified using a FavorPrep Blood/Cultured Cells total RNA extraction column (Favorgen Biotech) and then treated with Antarctic Phosphatase (New England Biolabs) at 37 °C for 30 min. The resulting mRNAs were purified using an RNeasy MinElute Cleanup Kit (QIAGEN).

### Cy3- and Cy5-labelling of RNA

Cy3- and Cy5-labelled RNAs were prepared by ligation using T4 RNA ligase (Ambion), pCp-Cy3 (Jena Bioscience) and pCp-Cy5 (Jena Bioscience). Cy3- and Cy5-labelling was performed with 150 pmol RNA using 10 U T4 RNA ligase, 3 nmol pCp-Cy3 or pCp-Cy5 and 10% (v/v) dimethyl sulfoxide in 10 µl at 16 °C for 36–48 h. The Cy3- and Cy5-labelled RNAs were purified using an RNeasy MinElute Cleanup Kit (Qiagen). After the recovery of RNAs, the RNA concentration was measured in a NanoDrop (Thermo Scientific). The labelling efficiencies of Cy3 and Cy5 were calculated from the absorbance of Cy3 and Cy5, respectively.

### Electrophoretic mobility shift assay

Mixtures of L-RNA and S-RNA (each 20–50 nM) in 20 mM phosphate buffer, pH 7.0, 1.5 mM MgCl_2_ and 150 mM KCl were heated at 80 °C for 3 min and then cooled at room temperature for 10 min. After the addition of L7Ae (50–1000 nM), the mixtures were incubated at room temperature for 10–30 min. The mixtures were mixed with 10 × loading buffer (0.25% bromophenol blue, 30% glycerol) and were subjected to electrophoresis through a native polyacrylamide gel with 0.5 × Tris/Borate/EDTA buffer at room temperature. After electrophoresis, the gels were stained for 10 min with SYBR Green I and II (Lonza). Images of the gels were collected using a Gel Doc EZ Imager (BIO-RAD) or a Typhoon FLA 7000 laser scanner (GE Healthcare).

### Atomic force microscopy

The RNA and RNP nanostructures were observed in solution. Their samples were prepared as described for the EMSA. The AFM images were collected using Nano Live Vision (Research Institute of Biomolecules Metrology Co.) or BIXAM (Olympus). Small cantilevers (9 µm long, 2 µm wide and 130 nm thick; BL-AC10EGS, Olympus) having a spring constant of ~0.1 N m^−1^ and a resonant frequency of ~300–600 kHz in water were used to scan the sample surface. The 320 × 240-pixel images were collected at a scan rate of 0.2 frames per second. A fresh mica surface was coated with 0.1% 3-aminopropyltriethoxy silane. The samples (20–50 nM RNA nanostructure with or without 600–1,000 nM protein) were prepared in RNP binding buffer and diluted five- (for RNP nanostructures) or tenfold (for RNA nanostructures) with AFM observation buffer (20 mM Tris-HCl (pH 7.6) and 10 mM MgCl_2_). Then, they were applied to the mica for 5 min at room temperature and were washed with buffer solution. The image sequences were analysed using ImageJ (http://imagej.nih.gov/ij/) software.

### FRET experiment

Cy3- and Cy5-labelled RNAs (each 50 nM) were heated at 80 °C for 3 min and then cooled at room temperature for 10 min in 20 mM phosphate buffer (pH 7.0) containing 1.5 mM MgCl_2_ and 150 mM KCl. Tween-20 (0.1%) and either protein buffer (20 mM phosphate buffer, pH 7.0, 1.5 mM MgCl_2_, 150 mM KCl and 40% glycerol) or L7Ae (600 nM) dissolved in protein buffer was added to the mixtures and incubated at room temperature for 10–30 min. The excitation wavelength was 512 nm and the emission and excitation bandwidths were 10 nm. Fluorescence measurements were carried out with Infinite M1000 Plate Reader (TECAN).

### Fluorescence measurement of biMGA

biMGA-conjugated RNA nanostructures (100 nM) were heated at 80 °C for 3 min and then cooled at room temperature for 10 min in 20 mM phosphate buffer (pH 7.0) containing 1.5 mM MgCl_2_ and 150 mM KCl. MG (2 µM), Tween-20 (0.1%) and either protein buffer (20 mM phosphate buffer, pH 7.0, 1.5 mM MgCl_2_, 150 mM KCl and 40% glycerol) or L7Ae (600 nM) dissolved in protein buffer were added to the mixtures and incubated at room temperature for 10–30 min. The excitation wavelength was 610 nm and the emission and excitation bandwidths were 10 and 20 nm, respectively. Fluorescence measurements were carried out with Infinite M1000 Plate Reader (TECAN).

### Cell cultures

HeLa cells (originally obtained from ATCC) were cultured at 37 °C in 5% CO_2_ in Dulbecco’s modified Eagle’s medium–High Glucose (Nacalai Tesque) containing 10% fetal bovine serum (Japan Bio Serum), non-essential amino acids (Invitrogen), sodium pyruvate (Sigma) and antibiotic antimycotic solution (Sigma). HeLa cells stably expressing Lin28A (a kind gift from Dr. Kozo Tomita, Tokyo University) were established by transfecting pIRES-puro vector containing Flag-tagged Lin28A sequence into HeLa cells. Positive clones were obtained by selection with puromycin (0.5 μg ml^−1^) for 2 weeks. Human iPS cells (201B7, a kind gift from Dr Masato Nakagawa, Kyoto University) were maintained in feeder-free condition^52^ with StemFit AK03 (Ajinomoto) on laminin-511 E8 (iMatrix-511, Nippi).

### RNA transfection

Mixtures of L-RNA and S-RNA (each 0.1–4 μM) in 20 mM phosphate buffer, pH 7.0, 1.5 mM MgCl_2_ and 150 mM KCl were heated at 80 or 95 °C for 3 min and then cooled at room temperature for 10 min. The mixtures of RNA nanostructures (0.1–8 pmol) and modRNAs (10–500 ng) were used for RNA delivery using Stemfect RNA Transfection Reagent (STEMGENT) according to the manufacturer’s protocol.

### Flow cytometric-based FRET assay

A total of 5 × 10^4^ cells were seeded into 12-well plates. After a 24 h culture, RNA nanodevices (8 pmol) and modRNA (500 ng) were co-transfected into the cells using 2 µl of Stemfect RNA Transfection Reagent (STEMGENT). The medium was changed after 2 h. After 12 or 24 h of transfection, the cells were washed with phosphate-buffered saline (PBS) and incubated in 200 µl of 0.25% Trypsin-EDTA (Nacalai Tesque) at 37 °C for 3 min. After the addition of 100 µl of Dulbecco’s PBS containing 2% fetal bovine serum, the cells were analysed with a FACSAria cell sorter (BD Bioscience). A 532 nm laser and a 575/25 nm optical filter were used to detect Cy3 fluorescence. A 532 nm laser and a 670/30 nm optical filter were used to detect the Cy5 fluorescence attributed to FRET. In the flow cytometric analysis, dead cells were removed using a dot plot of forward scatter (FSC) versus side scatter (SSC). The evaluation of the FRET signal change by the actuation of an RNA nanodevice was performed by calculating the mean of the ratio between the Cy3 and Cy5 fluorescence intensities. Enhancement of the Cy3/Cy5 mean was calculated from the Cy3/Cy5 mean normalized to cells transfected with 2dKt-17-Z-7 and either MS2CP- or L7Ae-coding modRNA. The obtained means were normalized to the mean of negative control cells transfected with MS2CP-conding modRNA and 2dKt-17-Z-7 for calculating relative Cy3/Cy5 mean intensities.

### Cell-death assay

A total of 5 × 10^4^ cells were seeded into 24-well plates. After a 24 h culture, the corresponding RNA nanostructures (0.1 pmol) and modRNA (10 ng) were co-transfected into the cells using 1 µl of Stemfect RNA Transfection Reagent (STEMGENT). After 2 h, the medium was changed. Cell images were taken after 24 h of transfection. The supernatants and cells were recovered and stained with Pacific Blue-labelled Annexin V (Life Technologies). Dead cells were stained with SYTOX AADvanced Dead Cell stain (Life Technologies), if needed. The stained cells were analysed using a FACSAria cell sorter and LSR Fortessa (BD Bioscience). A 405 laser and a 488 nm laser were used for the excitation of Pacific Blue and SYTOX AADvanced Dead Cell Stain, respectively; 450/50 and 660/20 nm optical filters were used to detect the fluorescence of Pacific Blue and SYTOX AADvanced Dead Cell Stain, respectively. Cells with high Pacific Blue fluorescence intensity were counted as apoptotic and dead cells.

### miRNA-responsive cell-death regulation assay

A total of 5 × 10^4^ cells (HeLa cells and hiPSCs) were seeded into 24-well plates. After a 24 h culture, the corresponding RNA nanostructure (0.1 pmol for HeLa cells and 0.2 pmol for hiPSCs) and miRNA-responsive modRNA switch (10 ng for HeLa cells and 30 ng for hiPSCs) were transfected into the cells using 1 µl of Stemfect RNA Transfection Reagent (STEMGENT). MiR-21-5p inhibitor (2 pmol) and miR-302a-5p mimic (0.5 pmol) were used for HeLa cells. MiR-21-5p mimic (2 pmol) and miR-302a-5p inhibitor (2 pmol) were used for hiPSCs. The following protocols were similar to ‘Cell-death assay.’

### Lin28A-responsive cell-death regulation assay

A total of 5 × 10^4^ cells (HeLa cells, HeLa-Lin28A cells and hiPSCs) were seeded into 24-well plates. After a 24 h culture, the corresponding RNA nanostructures (0.2 pmol for HeLa cells and HeLa-Lin28A cells, 0.4 pmol for hiPSCs) and Lin28A-dCasp-8 (40 ng for HeLa cells, HeLa-Lin28A cells and hiPSCs)- or L7Ae-dCasp-8 (10 ng for HeLa cells, HeLa-Lin28A cells, 30 ng for hiPSCs)-coding modRNA were co-transfected into the cells using 1 µl of Stemfect RNA Transfection Reagent (STEMGENT). Lin28A- or MS2CP-coding modRNA (40 ng for HeLa cells) was used as a competitor for the data in Fig. 6b. The following protocols were similar to ‘Cell-death assay.’

### Measurement of caspase activity

A total of 2 × 10^5^ HeLa cells were seeded into six-well plates and incubated for 24 h. The corresponding RNA nanostructures (0.4 pmol) and modRNA (40 ng) were co-transfected to the cells using 4 µl of Stemfect RNA Transfection Reagent (STEMGENT). After 2 h, the medium was changed. The measurement of caspase activity was performed after 6 h of transfection. The caspase activity was measured using an APOPCYTO Casp-8 Fluorometric Assay Kit (MBL), according to the manufacturer’s protocol. IETD-AMC (100 μM) and IETD-FMK (10 μM) were used as caspase substrate and inhibitor, respectively.

### Translational repression assay

A total of 5 × 10^4^ HeLa cells were seeded into 24-well plates and incubated for 24 h. K-turn (Kt) motif-containing EGFP (Kt-EGFP)-coding modRNA (100 ng) and a mixture of L7Ae- or L7Ae-dCasp-8-coding modRNA with MS2CP-coding modRNA (total 250 ng) were co-transfected into the cells using 1 µl Stemfect RNA Transfection Reagent (STEMGENT). The medium was changed after 2 h. After 24 h transfection, the cells were recovered after Trypsin-EDTA treatment and analysed with LSR Fortessa (BD Bioscience). A 488 nm laser and a 530/30 nm optical filter were used to detect EGFP fluorescence. In the flow cytometric analysis, dead cells were removed using a dot plot of FSC versus SSC. The evaluation of translational repression was performed by calculating the mean of the EGFP fluorescence intensities. The obtained means were normalized to the mean of cells transfected with Kt-EGFP-coding modRNA (100 ng) and MS2CP-coding modRNA (250 ng).

### Immunodetection of proteins

A total of 2 × 10^5^ cells were seeded into six-well plates. Cells transfected with various amounts of modRNAs encoding L7Ae-myc-His6, L7Ae-dCasp-8-myc-His6 and Lin28A were prepared in a similar manner as described in Cell-death assay using 4 μl Stemfect RNA Transfection Reagent (STEMGENT). After 24 h transfection, the supernatant and adhesive cells were collected and washed by PBS. The collected cells were lysed in M-PER Mammalian Protein Extraction Reagent (Thermo) for 5 min, then the debris of the cells were removed by centrifugation. The total amount of protein in the supernatant was determined by BCA assay kit (Thermo). GAPDH, Myc-tag and Lin28A were detected by an automated capillary-based immunodetection system (Wes SimpleWestern, ProteinSimple) using 100-fold diluted Anti-GAPDH (Santa Cruz, FL-355, sc-25778), 20-fold diluted Anti-Myc (Santa Cruz, sc-789) and 100-fold diluted Anti-Lin28A (R&D Systems, AF3757) antibodies as primary antibodies, respectively. HRP-conjugated Anti-rabbit (ProteinSimple, 042-206) and 100-fold diluted Anti-goat (Thermo, 81-1620) antibodies were used as secondary antibodies. The expression levels of the target proteins were quantified by Compass software (ProteinSimple).

### Statistical analysis

The statistical significance was analysed by Welch’s *t*-test (KaleidaGraph 4.5.1, HULINKS). The levels of significance are denoted as *P < 0.05, **P < 0.01, ***P < 0.001, NS not significant. All data are presented as the mean ± SD.

### Data availability

The data that support the findings of this study are available from the corresponding author upon request.

## Electronic supplementary material

Supplementary Information
