# Supplementary material for: Protein-driven RNA nanostructured devices that function in vitro and control mammalian cell fate
Source: Nat Commun. 2017 Sep 14;8:540. doi: 10.1038/s41467-017-00459-x (PMC5599586; doi:10.1038/s41467-017-00459-x)
Supplement: Supplementary file 1 — Supplementary Information [file 41467_2017_459_MOESM1_ESM.pdf]

Supplementary Information

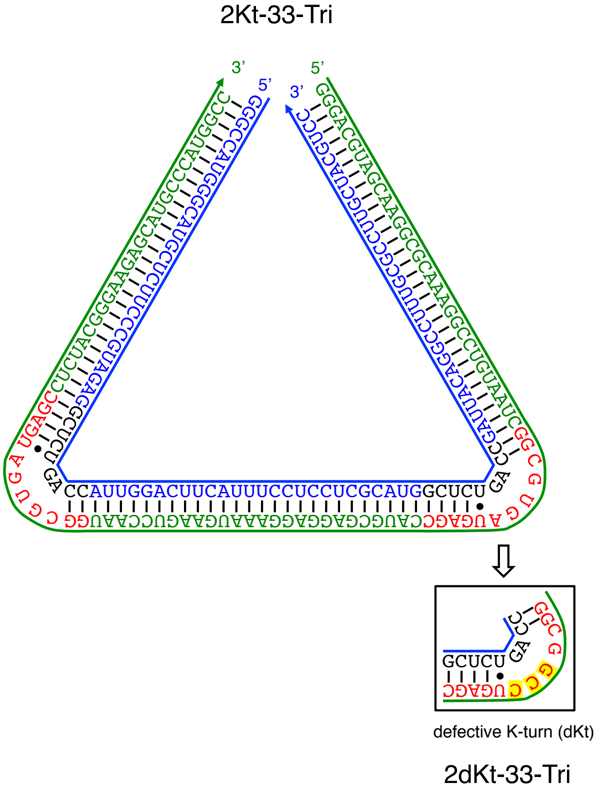

2Kt-33-Tri-long:
GGGACGUAGCAAGGCGCAAAGGCCUGUAAUCGGCGUGAUGAGCCAUGCGA
GGAGGAAAUGAAGUCCAAUGGCGUGAUGAGCCUCUACGGGAAGAGCAUGC
CCAUGGCC

2dKt-33-Tri-long:
GGGACGUAGCAAGGCGCAAAGGCCUGUAAUCGGCGGCCUGAGCCAUGCGA
GGAGGAAAUGAAGUCCAAUGGCGGCCUGAGCCUCUACGGGAAGAGCAUGC
CCAUGGCC

2Kt-33-Tri-short:
GGGCCAUGGGCAUGCUCUUCCCGUAGAGGCUCUGACCAUUGGACUUCAUU
UCCUCCUCGCAUGGCUCUGACCGAUUACAGGCCUUUGCGCCUUGCUACGU
CC

Supplementary Figure 1

2D structures and RNA sequence of 2Kt-33-Tri and 2dKt-33-Tri RNA nanostructures. The RNA nanostructures consist of two RNA strands: “long strand” (green) and “short strand” (blue). The two K-turn motifs are shown in red and black letters. Letters highlighted in yellow indicate the mutated nucleobases in the K-turn motifs.

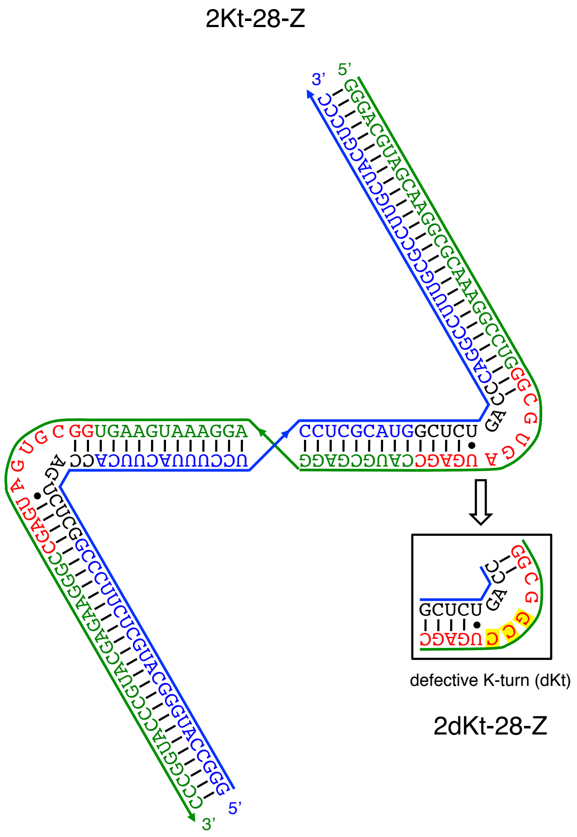

2Kt-28-Z-long: GGGACGUAGCAAGGCGCAAAGGCCUGGGCGUGAUGAGCCAUGCGAGGAGG
AAAUGAAGUGGCGUGAUGAGCCGGGAAGAGCAUGCCCAUGGCCC

2dKt-28-Z-long: GGGACGUAGCAAGGCGCAAAGGCCUGGGCGGCCUGAGCCAUGCGAGGAGG
AAAUGAAGUGGCGGCCUGAGCCGGGAAGAGCAUGCCCAUGGCCC

2Kt-28-Z-short: GGGCCAUGGGCAUGCUCUUCCCGGCUCUGACCACUUCAUUUCCUCCUCGC
AUGGCUCUGACCCAGGCCUUUGCGCCUUGCUACGUCCC

Supplementary Figure 2

2D structures and RNA sequence of 2Kt-28-Z and 2dKt-28-Z RNA nanostructures. The RNA nanostructures consist of two RNA strands: “long strand” (green) and “short strand” (blue). The two K-turn motifs are shown in red and black letters. Letters highlighted in yellow indicate the mutated nucleobases in the K-turn motifs.

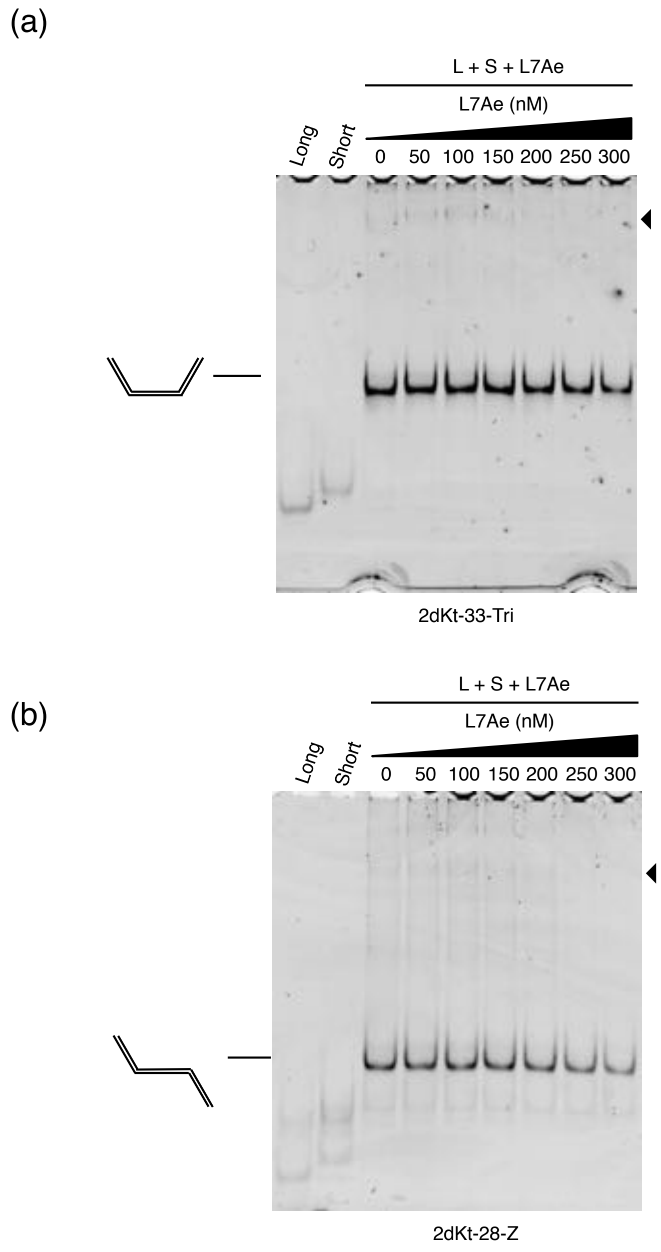

Supplementary Figure 3

EMSA to confirm the interaction between defective K-turn motifs and L7Ae. Interaction between (a) 2dKt-33-Tri or (b) 2dKt-28-Z RNA nanostructures and L7Ae. No retarded bands were observed for either of the RNA nanostructures with defective K-turn motifs (2dKt-33-Tri and 2dKt-28-Z). Higher order bands (black arrowheads) indicate heterogeneous oligomers composed of L- and S-RNA strands. Concentrations of long and short RNAs: each 50 nM.

(a)

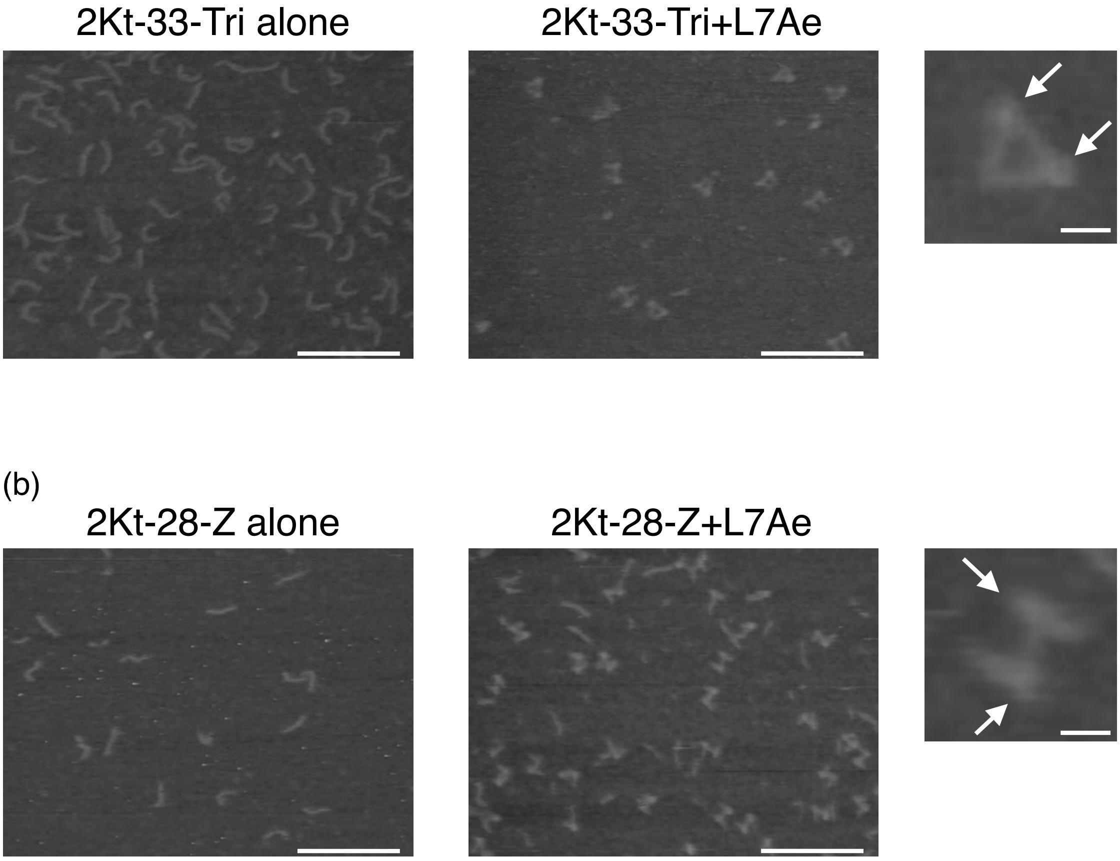

Supplementary Figure 4

AFM images of (a) 2Kt-33-Tri and (b) 2Kt-28-Z in the absence (left) and presence (right) of L7Ae. Scale bars: 100 nm. Enlarged images in the presence of L7Ae are shown on the right. White arrows represent a single L7Ae protein. Scale bars: 10 nm.

Cy3, Cy5-labeled 2Kt-33-Tri

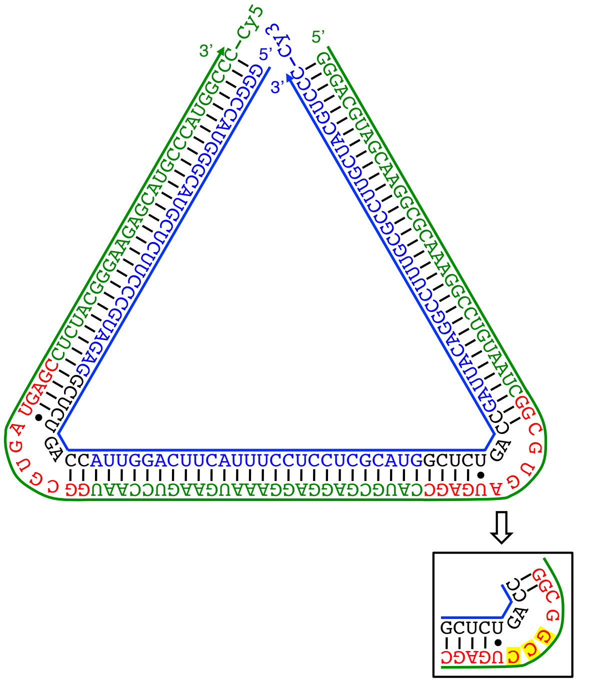

defective K-turn (dKt)

2dKt-33-Tri

2Kt-33-Tri-long-Cy5: GGGACGUAGCAAGGCGCAAAGGCCUGUAAUCGGCGUGAUGAGCCAUGCGA GGAGGAAAUGAAGUCCAAUGGCGUGAUGAGCCUCUACGGGAAGAGCAUGC CCAUGGCCC(Cy5)

2dKt-33-Tri-long-Cy5: GGGACGUAGCAAGGCGCAAAGGCCUGUAAUCGGCGGCCUGAGCCAUGCGA GGAGGAAAUGAAGUCCAAUGGCGGCCUGAGCCUCUACGGGAAGAGCAUGC CCAUGGCCC(Cy5)

2Kt-33-Tri-short-Cy3: GGGCCAUGGGCAUGCUCUUCCCGUAGAGGCUCUGACCAUUGGACUUCAUU UCCUCCUCGCAUGGCUCUGACCGAUUACAGGCCUUUGCGCCUUGCUACGU CCC(Cy3)

Supplementary Figure 5

2D structures and RNA sequence of Cy3- and Cy5-labelled 2Kt-33-Tri and 2dKt-33-Tri RNA nanostructures. The RNA nanostructures consist of two RNA strands: “Cy5-labelled long strand” (green) and “Cy3-labelled short strand” (blue). The two K-turn motifs are shown in red and black letters. Letters highlighted in yellow indicate the mutated nucleobases in the K-turn motifs.

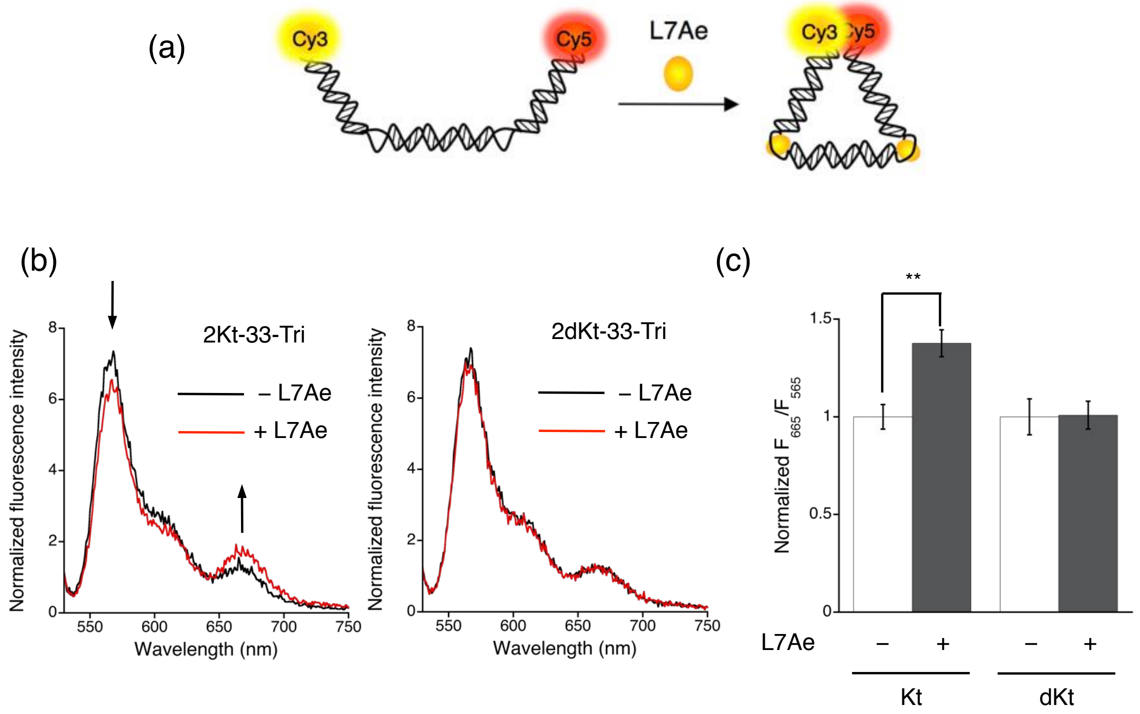

Supplementary Figure 6

(a) Schematic illustration of FRET experiments. (b) Fluorescence spectra of Cy3- and Cy5-labelled 2Kt-33-Tri (left) and 2dKt-33-Tri (right) in the absence (black) and presence (red) of L7Ae. (c) Quantification of the FRET signal change. The data are presented as the mean ± SD (n = 3). **P < 0.01 (Welch's t-test).

Tri-MGA-ON-U0, U2, U4

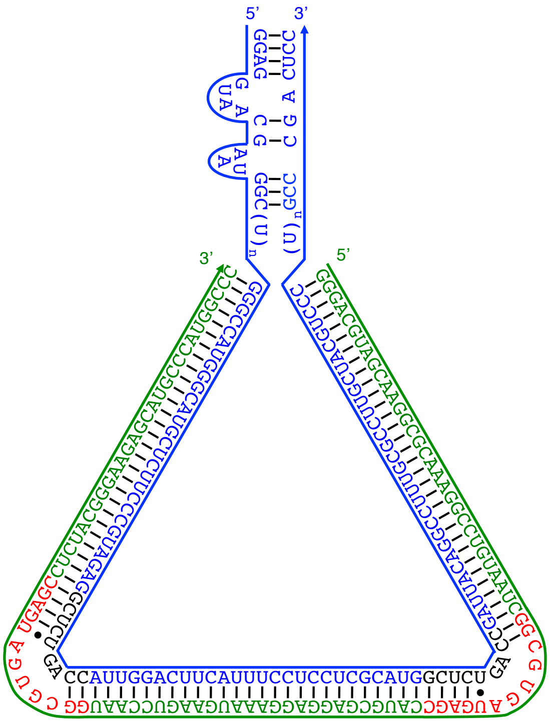

10 20 30 40 50

2Kt-33-Tri-long+C: GGGACGUAGCAAGGCGCAAAGGCCUGUAAUCGGCGUGAUGAGCCAUGCGA
GGAGGAAAUGAAGUCCAAUGGCGUGAUGAGCCUCUACGGGAAGAGCAUGC
CCAUGGCCC

10 20 30 40 50

Tri-MGA-ON-short-U0: GGAGGUAACGAAUGGCGGGCCAUGGGCAUGCUCUUCCCGUAGAGGCUCUG
(original stem) ACCAUUGGACUUCAUUUCCUCCUCGCAUGGCUCUGACCGAUUACAGGCCU
UUGCGCCUUGCUACGUCCCGCCCGACUCC

10 20 30 40 50

Tri-MGA-ON-short-U2: GGAGGUAACGAAUGGCUUGGGCCAUGGGCAUGCUCUUCCCGUAGAGGCUC
(original stem) UGACCAUUGGACUUCAUUUCCUCCUCGCAUGGCUCUGACCGAUUACAGGC
CUUUGCGCCUUGCUACGUCCCUUGCCCGACUCC

10 20 30 40 50

Tri-MGA-ON-short-U4: GGAGGUAACGAAUGGCUUUUGGGCCAUGGGCAUGCUCUUCCCGUAGAGGC
(original stem) UCUGACCAUUGGACUUCAUUUCCUCCUCGCAUGGCUCUGACCGAUUACAG
GCCUUUGCGCCUUGCUACGUCCCUUUUGCCCGACUCC

Supplementary Figure 7

2D structures and RNA sequence of Tri-MGA-ON-U0, Tri-MGA-ON-U2, and Tri-MGA-ON-U4 RNA nanostructures with different linker lengths. The RNA nanostructures consist of two RNA strands: “long strand” (green) and “biMGA-conjugated short strand” (blue). The two K-turn motifs are shown in red and black letters. BiMGA sequences are underlined.

Z-MGA-OFF-U0, U2, U4

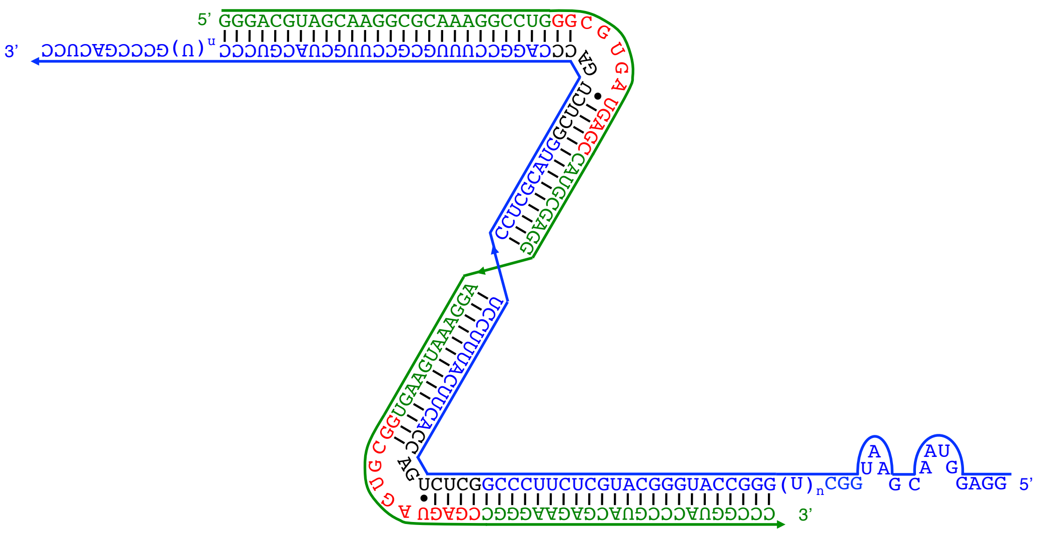

2Kt-28-Z-long: GGGACGUAGCAAGGCGCAAAGGCCUGGGCGUGAUGAGCCAUGCGAGGAGG
AAAUGAAGUGGCGUGAUGAGCCGGGAAGAGCAUGCCCAUGGCCC

Z-MGA-OFF-short-U0: (original stem) GGAGGUAACGAAUGGCGGGCCAUGGGCAUGCUCUUCCCGGCUCUGACCAC
UUCAUUUCCUCCUCGCAUGGCUCUGACCCAGGCCUUUGCGCCUUGCUACG
UCCCGCCCGACUCC

Z-MGA-OFF-short-U2: (original stem) GGAGGUAACGAAUGGCUUGGCCAUGGGCAUGCUCUUCCCGGCUCUGACC
ACUUCAUUUCCUCCUCGCAUGGCUCUGACCCAGGCCUUUGCGCCUUGCUA
CGUCCCUUGCCCGACUCC

Z-MGA-OFF-short-U4: (original stem) GGAGGUAACGAAUGGCUUUUGGGCCAUGGGCAUGCUCUUCCCGGCUCUGA
CCACUUCAUUUCCUCCUCGCAUGGCUCUGACCCAGGCCUUUGCGCCUUGC
UACGUCCCUUUUGCCCGACUCC

Supplementary Figure 8

2D structures and RNA sequence of Z-MGA-OFF-U0, Z-MGA-OFF-U2, and Z-MGA-OFF-U4 RNA nanostructures with different linker lengths. The RNA nanostructures consist of two RNA strands: “long strand” (green) and “biMGA-conjugated short strand” (blue). The two K-turn motifs are shown in red and black letters. BiMGA sequences are underlined.

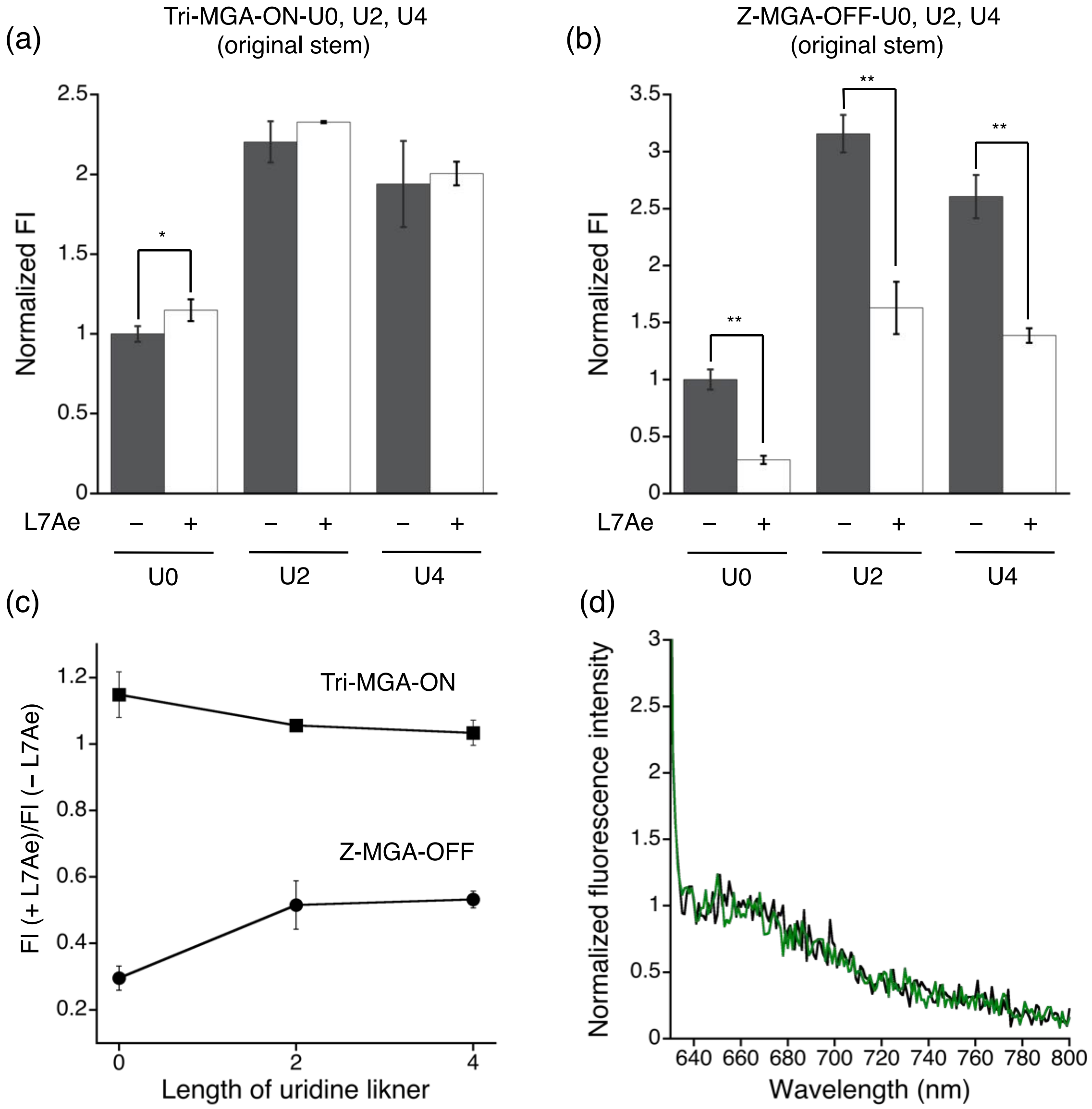

Supplementary Figure 9

Optimization of the uridine linker length of Tri-MGA-ON and Z-MGA-OFF for the switching of biMGA activity. Normalized fluorescence intensity (FI) of (a) Tri-MGA-ON and (b) Z-MGA-OFF RNA nanostructures in the absence (grey) and presence (white) of L7Ae. (c) Plots of fold change FI (+ L7Ae)/FI (− L7Ae) versus length of uridine linker for Tri-MGA-ON (square) and Z-MGA-OFF (circle). (d) Fluorescence spectra of malachite green in the absence (black) and presence (green) of L7Ae. The emission of malachite green is not affected by L7Ae. The data are presented as the mean ± SD (n = 3). *$P < 0.05$, **$P < 0.01$ (Welch's t-test).

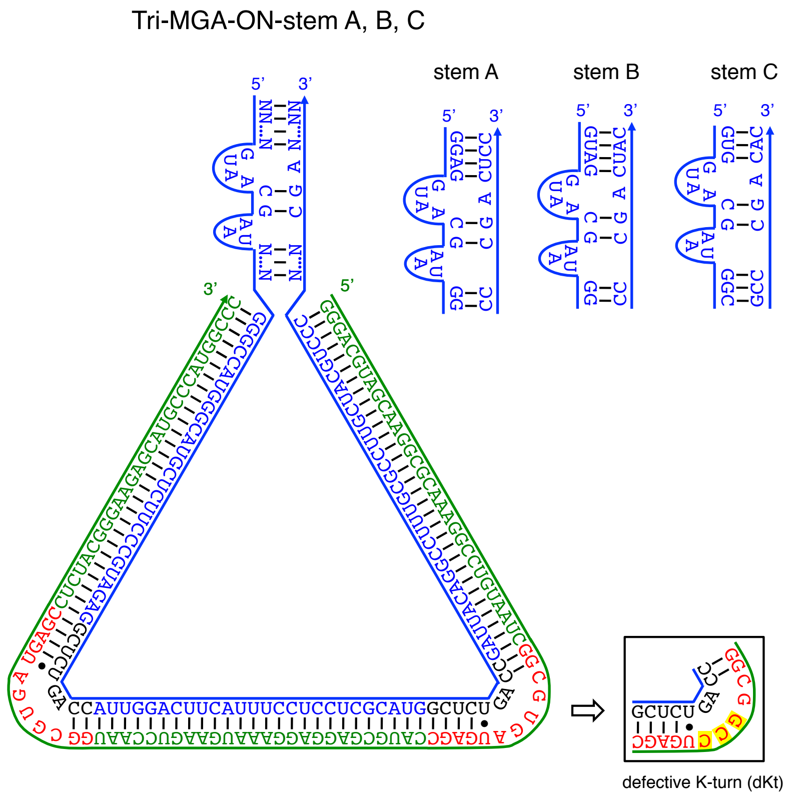

2Kt-33-Tri-long+C: GGGACGUAGCAAGGCGCAAAGGCCUGUAAUCGGCGUGAUGAGCCAUGCGA
GGAGGAAAUGAAGUCCAAUGGCGUGAUGAGCCUCUACGGGAAGAGCAUGC
CCAUGGCCC

2dKt-33-Tri-long+C: GGGACGUAGCAAGGCGCAAAGGCCUGUAAUCGGCGGCCUGAGCCAUGCGA
GGAGGAAAUGAAGUCCAAUGGCGGCCUGAGCCUCUACGGGAAGAGCAUGC
CCAUGGCCC

Tri-MGA-ON-short-U0 (stem A): GGAGGUAACGAAUGGGGGCCAUGGGCAUGCUCUUCCCGUAGAGGCUCUGA
CCAUUGGACUUCAUUUCCUCCUCGCAUGGCUCUGACCGAUUACAGGCCUU
UGCGCCUUGCUACGUCCCCCCGACUCC

Tri-MGA-ON-short-U0 (stem B): GUAGGUAACGAAUGGGGGCCAUGGGCAUGCUCUUCCCGUAGAGGCUCUGA
CCAUUGGACUUCAUUUCCUCCUCGCAUGGCUCUGACCGAUUACAGGCCUU
UGCGCCUUGCUACGUCCCCCCGACUAC

Tri-MGA-ON-short-U0 (stem C): GUGGUAACGAAUGGCGGGCCAUGGGCAUGCUCUUCCCGUAGAGGCUCUGA
CCAUUGGACUUCAUUUCCUCCUCGCAUGGCUCUGACCGAUUACAGGCCUU
UGCGCCUUGCUACGUCCCGCCCGACAC

Supplementary Figure 10

2D structures and RNA sequence of Tri-MGA-ON-U0 RNA nanostructures with different stem sequences of biMGA. The RNA nanostructures consist of two RNA strands: "long strand" (green) and "biMGA-conjugated short strand" (blue). The two K-turn motifs are shown in red and black letters. BiMGA sequences are underlined. Letters highlighted in yellow indicate the mutated nucleobases in the K-turn motifs.

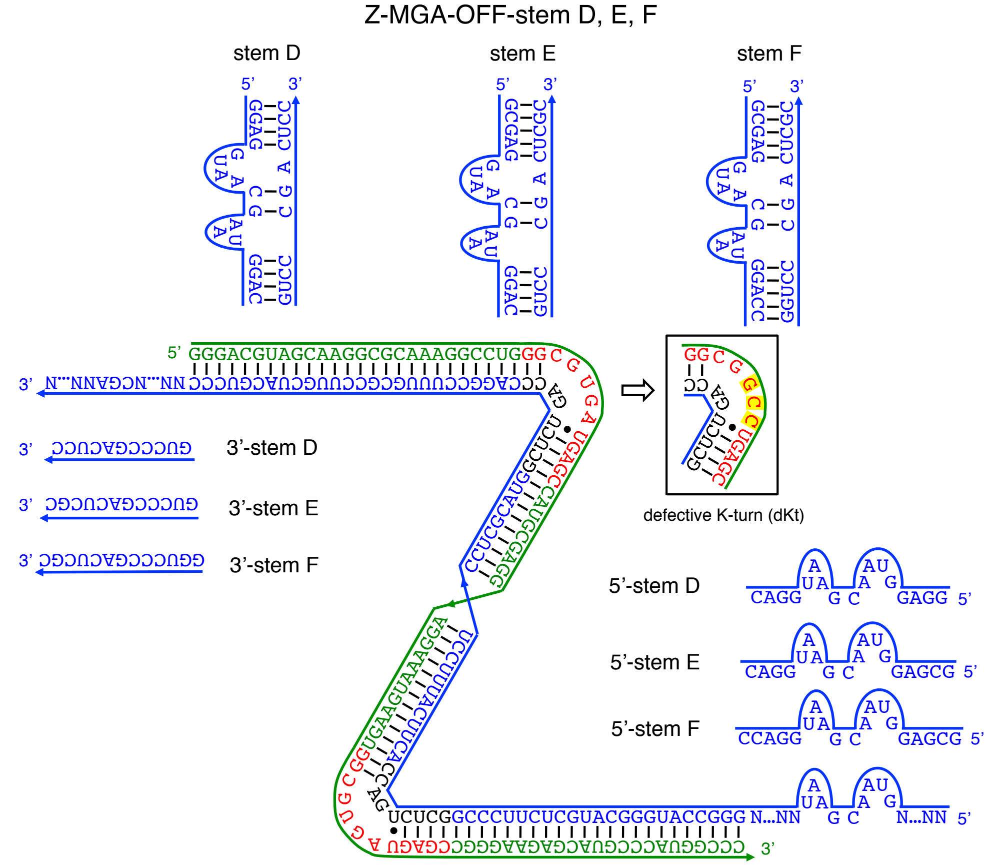

2Kt-28-Z-long: GGGACGUAGCAAGGCGCAAAGGCCUGGGCGUGAUGAGCCAUGCGAGGAGG
AAAUGAAGUGGCGUGAUGAGCCGGGAAGAGCAUGCCCAUGGCCC

2dKt-28-Z-long: GGGACGUAGCAAGGCGCAAAGGCCUGGGCGGCCUGAGCCAUGCGAGGAGG
AAAUGAAGUGGCGGCCUGAGCCGGGAAGAGCAUGCCCAUGGCCC

Z-MGA-OFF-short-U0: (stem D) GGAGGUAACGAAUGGACGGGCCAUGGGCAUGCUCUUCCCGGCUCUGACCA
CUUCAUUUCCUCCUCGCAUGGCUCUGACCCAGGCCUUUGCGCCUUGCUAC
GUCCCGUCCCGACUCC

Z-MGA-OFF-short-U0: (stem E) GCGAGGUAACGAAUGGACGGGCCAUGGGCAUGCUCUUCCCGGCUCUGACC
ACUUCAUUUCCUCCUCGCAUGGCUCUGACCCAGGCCUUUGCGCCUUGCUA
CGUCCCGUCCCGACUCGC

Z-MGA-OFF-short-U0: (stem F) GCGAGGUAACGAAUGGACCGGGCCAUGGGCAUGCUCUUCCCGGCUCUGAC
CACUUCAUUUCCUCCUCGCAUGGCUCUGACCCAGGCCUUUGCGCCUUGCU
ACGUCCCGGUCCCGACUCGC

Supplementary Figure 11

2D structures and RNA sequence of Z-MGA-OFF RNA nanostructures with different stem sequences of biMGA. The RNA nanostructures consist of two RNA strands: "long strand" (green) and "biMGA-conjugated short strand" (blue). The two K-turn motifs are shown in red and black letters. BiMGA sequences are underlined. Letters highlighted in yellow indicate the mutated nucleobases in the K-turn motifs.

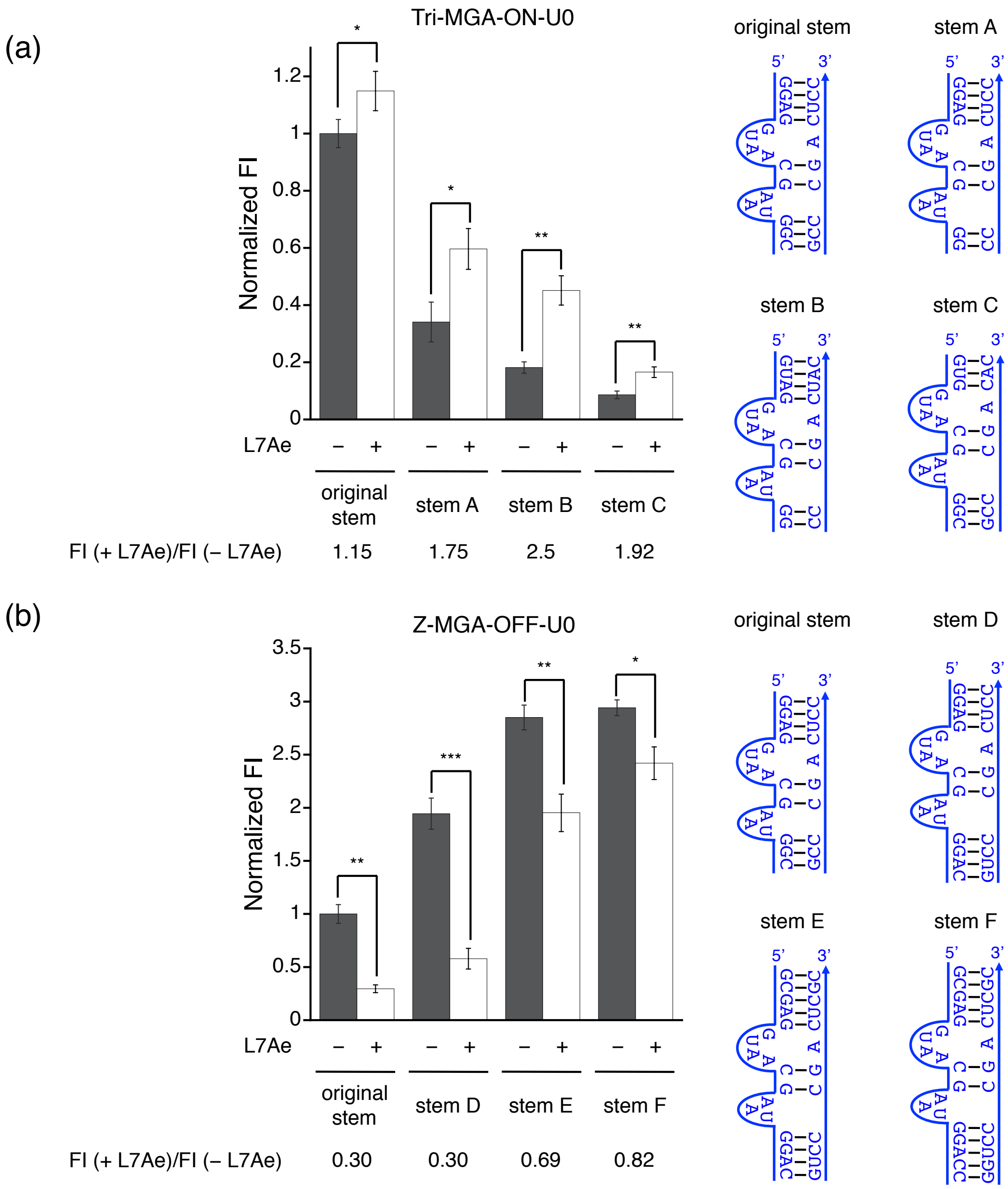

Supplementary Figure 12

Optimization of biMGA stem sequence in Tri-MGA-ON-U0 and Z-MGA-OFF-U0 RNA nanostructures for the switching of biMGA activity. Normalized fluorescence intensity (FI) of (a) Tri-MGA-ON-U0 and (b) Z-MGA-OFF-U0 RNA nanostructures in the absence (grey) and presence (white) of L7Ae. The data are presented as the mean ± SD (n = 3). *$P < 0.05$, **$P < 0.01$, ***$P < 0.001$ (Welch's t-test).

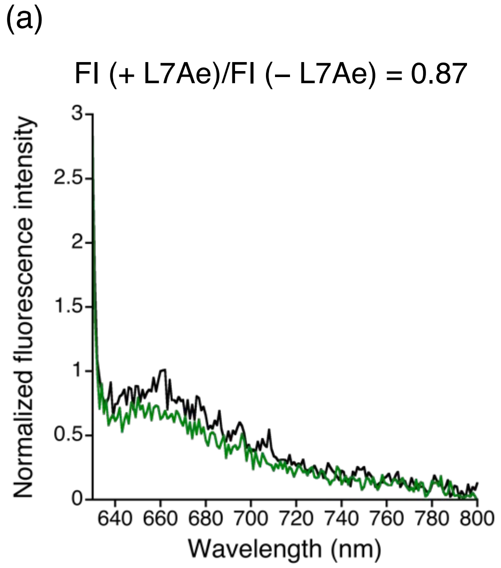

Supplementary Figure 13

Fluorescence spectra of (a) dKt-Tri-MGA-ON-U0-stem B and (b) dKt-Z-MGA-OFF-U0-stem D RNA nanostructures in the absence (black) and presence (green) of L7Ae.

(a)

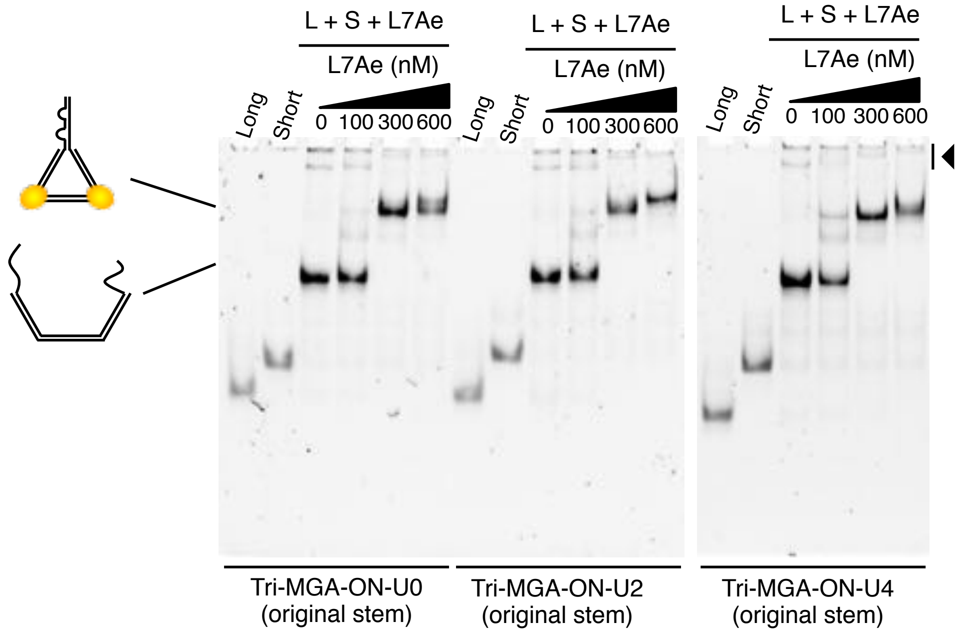

(b)

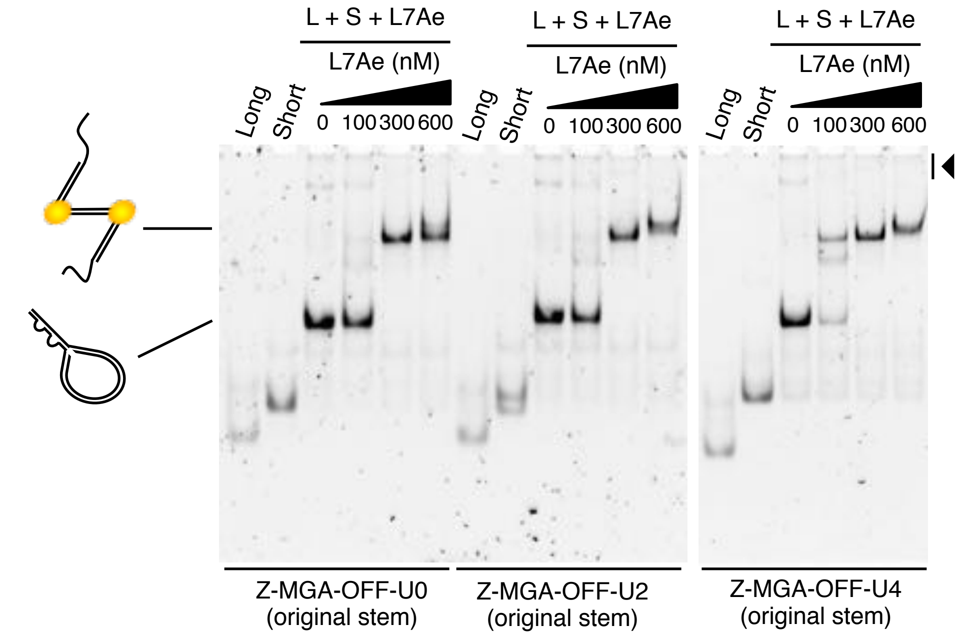

Supplementary Figure 14

EMSA to confirm interactions between the Tri-MGA-ON or Z-MGA-OFF RNA nanostructures and L7Ae. (a) Tri-MGA-ON-U0, Tri-MGA-ON-U2, and Tri-MGA-ON-U4 with the original stem. (b) Z-MGA-OFF-U0, Z-MGA-OFF-U2, and Z-MGA-OFF-U4 with the original stem. Higher order bands (black arrowheads) indicate heterogeneous oligomers composed of L- and S-RNA strands. Concentrations of long and short RNAs: each 50 nM.

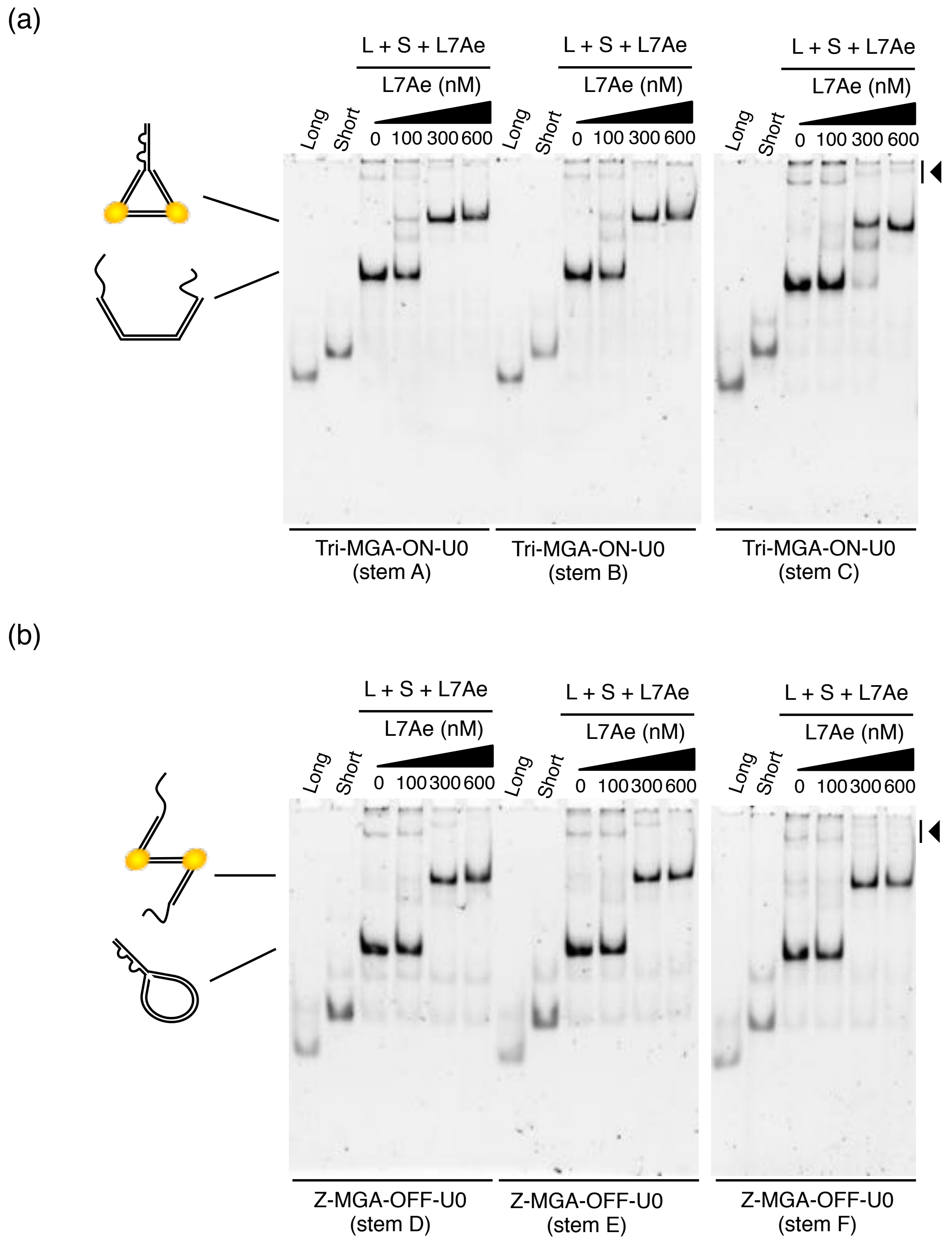

Supplementary Figure 15

EMSA to confirm interactions between the Tri-MGA-ON or Z-MGA-OFF RNA nanostructures and L7Ae. (a) Tri-MGA-ON-U0 with stems A, B, and C. (b) Z-MGA-OFF-U0 with stems D, E, and F. Higher order bands (black arrowheads) indicate heterogeneous oligomers composed of L- and S-RNA strands. Concentrations of long and short RNAs: each 50 nM.

(a)

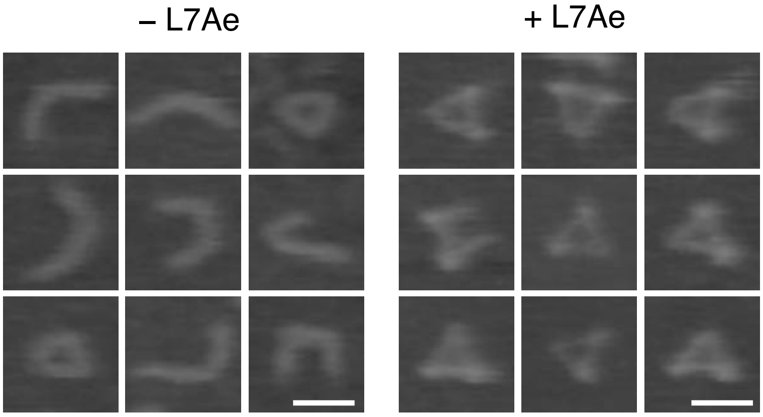

Tri-MGA-ON-U0-stem B

(b)

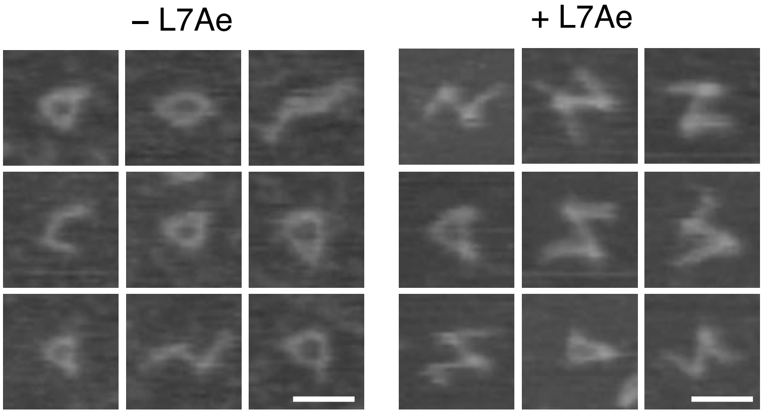

Z-MGA-OFF-U0-stem D

Supplementary Figure 16

AFM images of (a) Tri-MGA-U0-stem B and (b) Z-MGA-OFF-U0-stem D in the absence (left) and presence (right) of L7Ae. Scale bars: 20 nm.

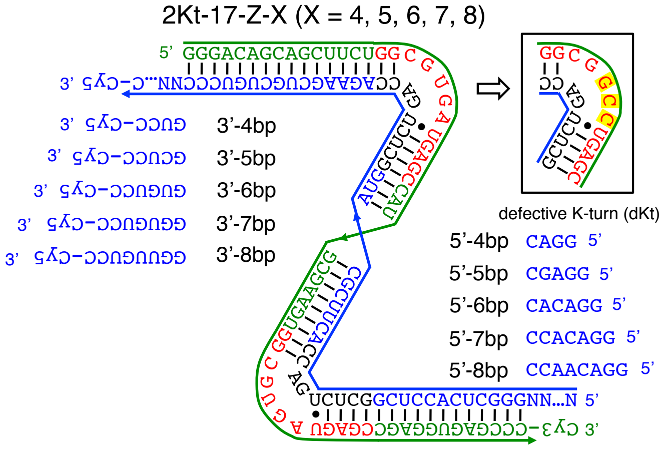

2Kt-17-Z-long-Cy3: GGGACAGCAGCUUCUGGCGUGAUGAGCCAUGCGAAGUGGCGUGAUGAGCC
GAGGUGAGCCC(Cy3)

2dKt-17-Z-long-Cy3: GGGACAGCAGCUUCUGGCGGCCUGAGCCAUGCGAAGUGGCGGCCUGAGCC
GAGGUGAGCCC(Cy3)

2Kt-17-Z-short-4-Cy5: GGACGGGCUCACCUCGGCUCUGACCACUUCGCAUGGCUCUGACCAGAAGC
UGCUGUCCCGUCC(Cy5)

2Kt-17-Z-short-5-Cy5: GGAGCGGGCUCACCUCGGCUCUGACCACUUCGCAUGGCUCUGACCAGAAG
CUGCUGUCCCGCUCC(Cy5)

2Kt-17-Z-short-6-Cy5: GGACACGGGCUCACCUCGGCUCUGACCACUUCGCAUGGCUCUGACCAGAA
GCUGCUGUCCCGUGUCC(Cy5)

2Kt-17-Z-short-7-Cy5: GGACACCGGGCUCACCUCGGCUCUGACCACUUCGCAUGGCUCUGACCAGA
AGCUGCUGUCCCGGUGUCC(Cy5)

2Kt-17-Z-short-8-Cy5: GGACAACCGGGCUCACCUCGGCUCUGACCACUUCGCAUGGCUCUGACCAG
AAGCUGCUGUCCCGGUUGUCC(Cy5)

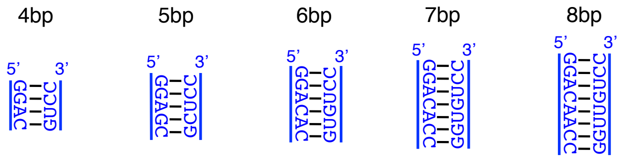

Supplementary Figure 17

2D structures and RNA sequence of 2Kt-17-Z-X RNA nanostructures with RNA duplexes of different stem lengths. The RNA nanostructures consist of two RNA strands: "long strand" (green) and "short RNA duplex-conjugated short strand" (blue). The two K-turn motifs are shown in red and black letters. The sequence of the short RNA duplexes is underlined.

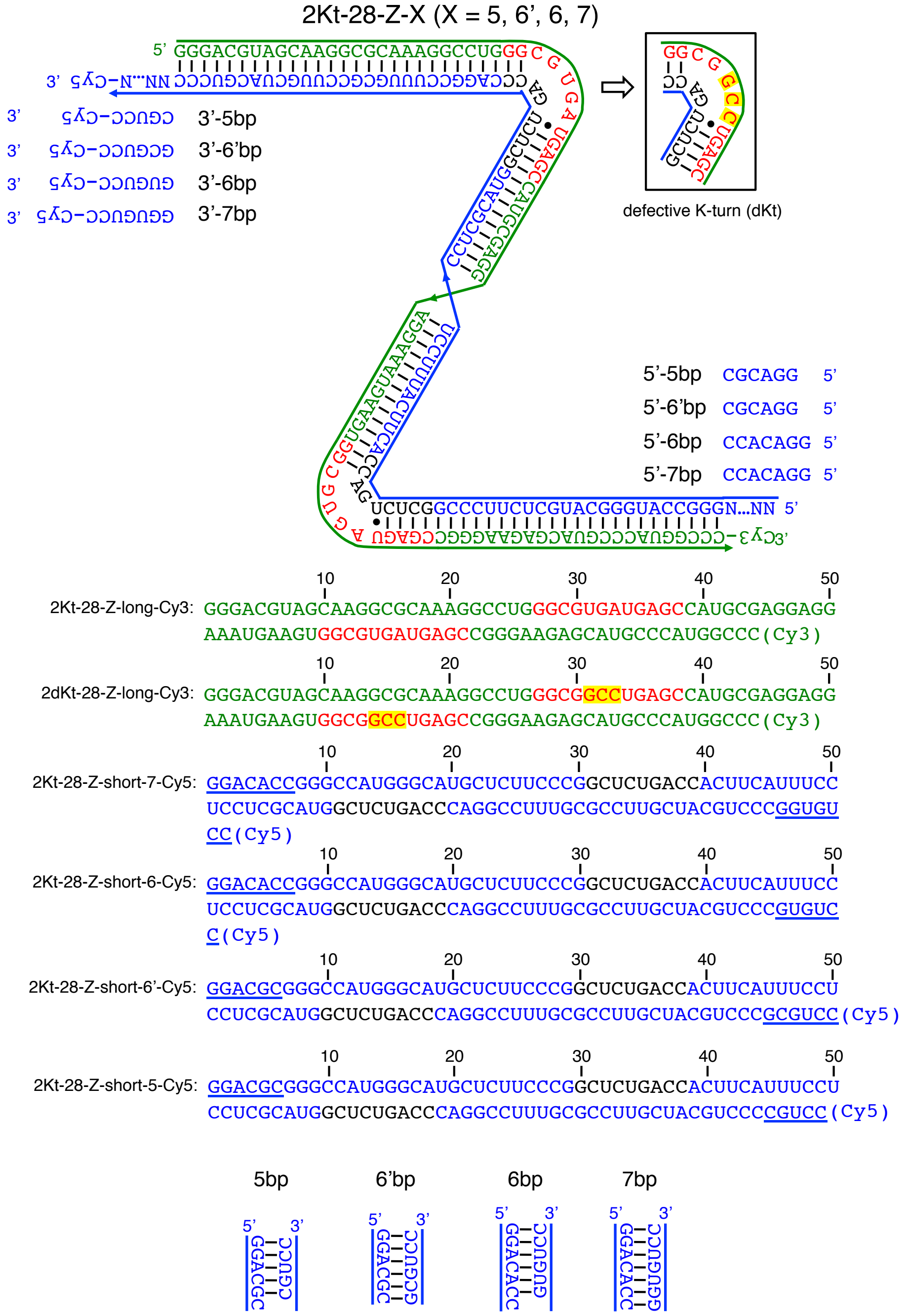

Supplementary Figure 18

2D structures and RNA sequences of 2Kt-28-Z-X RNA nanostructures with RNA duplexes of different stem lengths. The RNA nanostructures consist of two RNA strands: "long strand" (green) and "short RNA duplex-conjugated short strand" (blue). The two K-turn motifs are shown in red and black letters. The sequence of the short RNA duplexes is underlined.

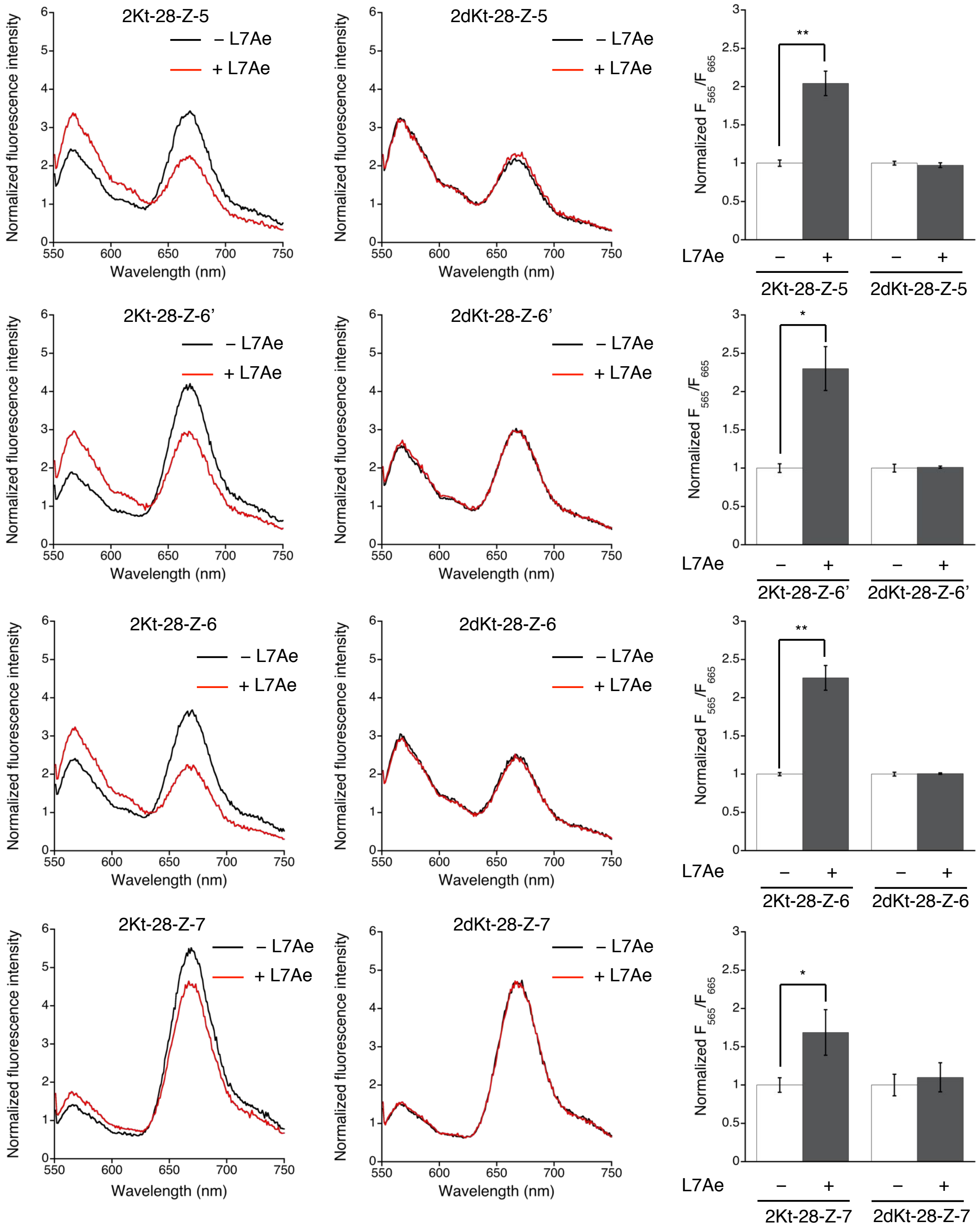

Supplementary Figure 19

Fluorescence spectra of 2Kt-28-Z-X (left) and 2dKt-28-Z-X (middle) in the absence (black) and presence (red) of L7Ae. Fold change of F_{565}/F_{665} in the absence (white) and presence (gray) of L7Ae (right). The data are presented as the mean ± SD (n = 3). *P < 0.05, **P < 0.01 (Welch’s t-test).

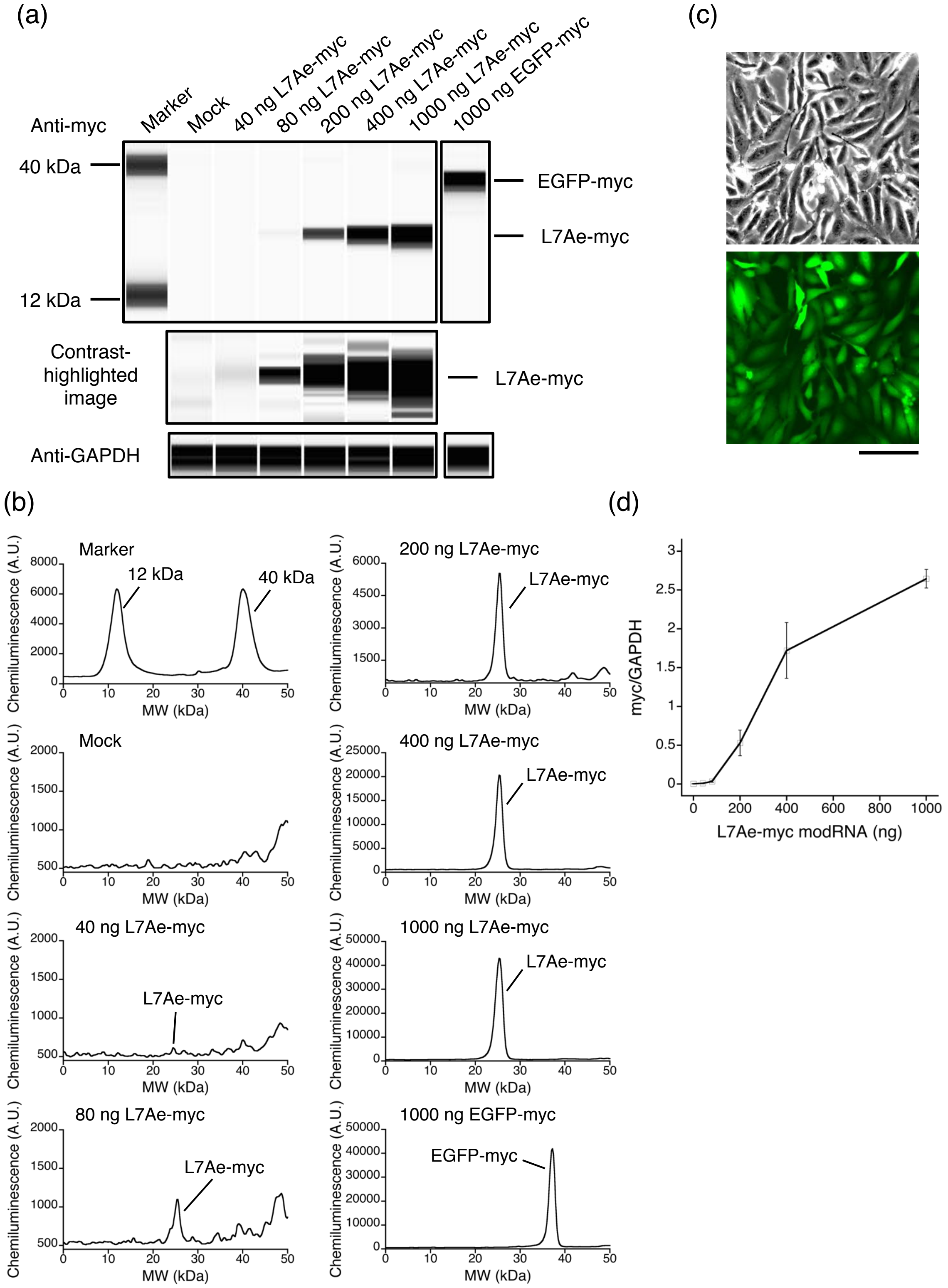
(a)
Anti-myc
Marker
Mock
40 ng L7Ae-myc
80 ng L7Ae-myc
200 ng L7Ae-myc
400 ng L7Ae-myc
1000 ng L7Ae-myc
1000 ng EGFP-myc
40 kDa
12 kDa
EGFP-myc
L7Ae-myc
Contrast-highlighted image
L7Ae-myc
Anti-GAPDH
(b)
Marker
12 kDa
40 kDa
200 ng L7Ae-myc
L7Ae-myc
Mock
400 ng L7Ae-myc
L7Ae-myc
40 ng L7Ae-myc
L7Ae-myc
1000 ng L7Ae-myc
L7Ae-myc
80 ng L7Ae-myc
L7Ae-myc
1000 ng EGFP-myc
EGFP-myc
Chemiluminescence (A.U.)
MW (kDa)
(c)
(d)
myc/GAPDH
L7Ae-myc modRNA (ng)

Supplementary Figure 20

(a) Virtual blot-like images and (b) electropherogram of capillary electrophoresis immunoassay of L7Ae-myc and EGFP-myc using anti-myc antibody. GAPDH was detected using anti-GAPDH antibody and was used as a control. (c) Fluorescence microscopic image of cells 24 h after transfection with EGFP-myc modRNA. Scale bar: 100 μm. (d) Relative expression level of L7Ae-myc. The data are presented as the mean ± SD (n = 3).

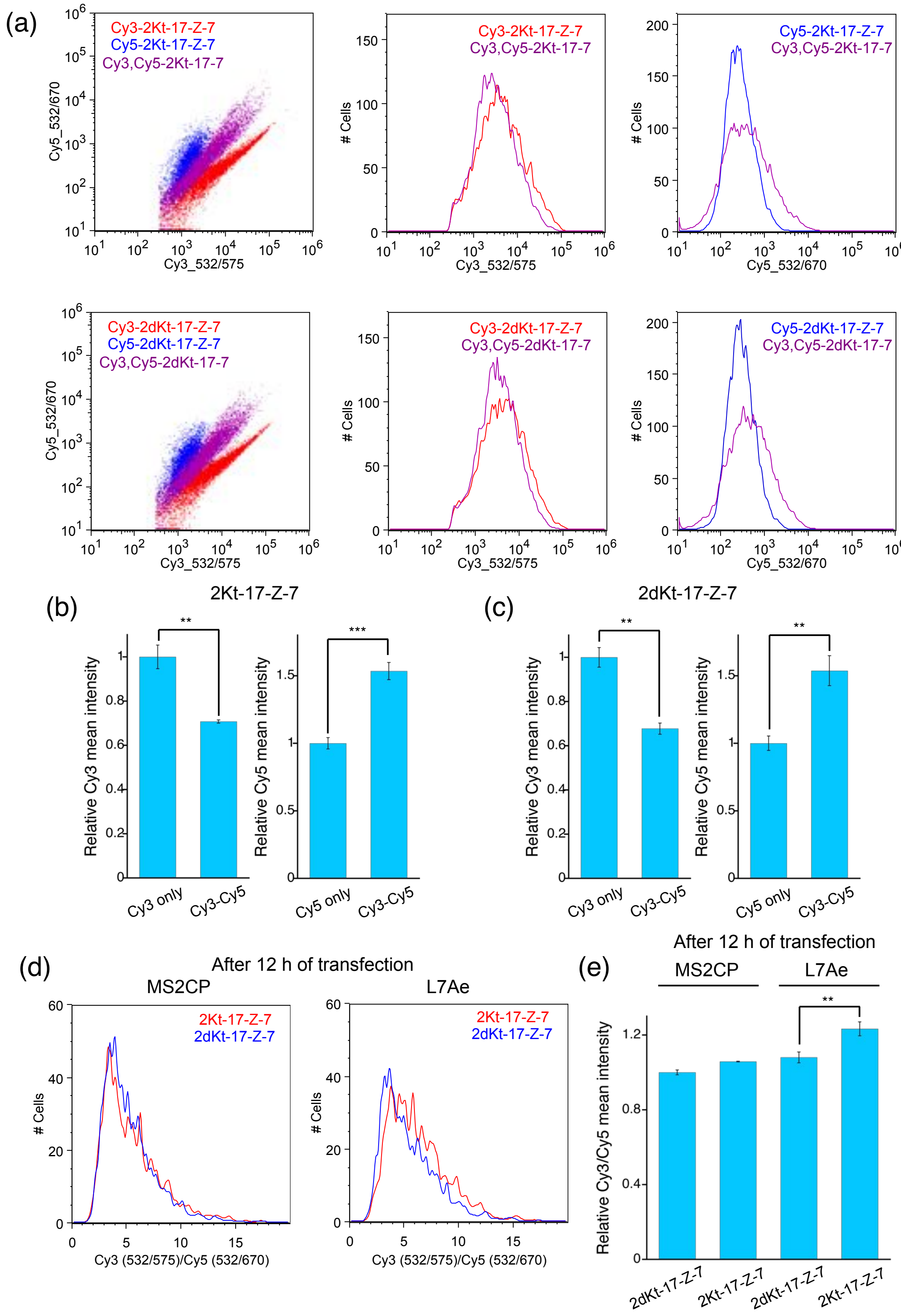
(a)
Cy3-2Kt-17-Z-7
Cy5-2Kt-17-Z-7
Cy3,Cy5-2Kt-17-7
Cy5_532/670
Cy3_532/575
Cells
Cy3-2dKt-17-Z-7
Cy5-2dKt-17-Z-7
Cy3,Cy5-2dKt-17-7
(b)
2Kt-17-Z-7
Relative Cy3 mean intensity
Relative Cy5 mean intensity
Cy3 only
Cy3-Cy5
Cy5 only
(c)
2dKt-17-Z-7
(d)
After 12 h of transfection
MS2CP
L7Ae
2Kt-17-Z-7
2dKt-17-Z-7
Cy3 (532/575)/Cy5 (532/670)
(e)
After 12 h of transfection
MS2CP
L7Ae
Relative Cy3/Cy5 mean intensity
2dKt-17-Z-7
2Kt-17-Z-7

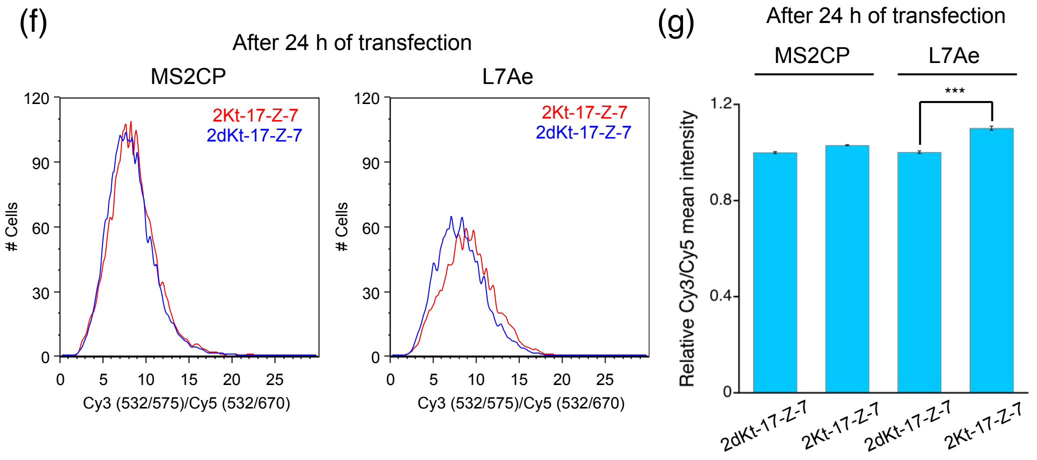

Supplementary Figure 21

(a) Two-dimensional plots of Cy3 (532/575) versus Cy5 (532/670) (left) and histogram of Cy3 (532/575) (middle) and Cy5 (532/670) (right) after 24 hours of transfection with 2Kt-17-Z-7 (top) or 2dKt-17-Z-7 (bottom) and MS2CP modRNA. Cy3-only, Cy5-only, and Cy3-Cy5-labeled RNA are shown in red, blue, and purple, respectively. (b), (c) Relative Cy3 (left) and Cy5 (right) mean intensity of Cy3-only, Cy5-only, and Cy3-Cy5-labeled 2Kt-17-Z-7 (b) and 2dKt-17-Z-7 (c). (d)–(g) Histograms of Cy3 (532/575)/Cy5 (532/670) after 12 (d) and 24 (f) hours of transfection of MS2CP modRNA (left) or L7Ae modRNA (right). Relative Cy3/Cy5 mean intensities after 12 (e) and 24 (g) hours transfection. Relative Cy3/Cy5 mean intensities were calculated by normalizing with the Cy3/Cy5 mean intensity of cells co-transfected with MS2CP modRNA and dKt-17-Z-7. The data are presented as the mean ± SD (n = 3). $^{**}P < 0.01$, $^{***}P < 0.001$ (Welch's t-test).

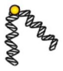

1Kt-33-Tri-long: GGGUAGCAAGAGUGAGGAGCUGUUCACAAGAGGCAUGAUGAACGUGGUGCUCAUCCUGGUCGAGCUGUCCC

1Kt-33-Tri-short: GGGACAGCUCGACCAGGAUGAGCACCACGUUCUGACCUCUUGUGAACAGCUCCUCACUCUUGCUACCC

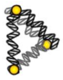

3Kt-33-Tri-long: GGGUAGCAAGAGUGAGGAGCUGUUCACAAGAGGCAUGAUGAACGUGGUGCUCAUCCUGGUCGAGCUGUAAGCGU
GAUCGAUGACACGUAACAGGUCACAAGUUCAGCCGCAUGAUCAGAGUCAGACGAGUUCGAGAUCGAUGACCCC

3Kt-33-Tri-short: GGGGUCAUCGAUCUCGAACUCGUCUGACUCUGUGACGGCUGAACUUGUGACCUGUUACGUGUCAUCGUGACUUA
CAGCUCGACCAGGAUGAGCACCACGUUCUGACCUCUUGUGAACAGCUCCUCACUCUUGCUACCC

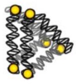

6Kt-33-Tri-long: GGGUAGCAAGAGUGAGGAGCUGUUCACAAGAGGCAUGAUGAACGUGGUGCUCAUCCUGGUCGAGCUGUAAGCGU
GAUCGAUGACACGUAACAGGUCACAAGUUCAGCCGCAUGAUCAGAGUCAGACGAGUUCGAGAUCGAUGACAUGC
AUGAUGCUUUCAGGACAGCUGACCUGUCAGUUCAUGGCGUGAUGAGCGCACCACUUGCAAGCUGCCAGUGCAAU
GCAUGAUCUCGCACCACUCUCGUGACCACGCUGAACCCC

6Kt-33-Tri-short: GGGGUUCAGCGUGGUCACGAGAGUGGUGCGAGUGACAUUGCACUGGCAGCUUGCAAGUGGUGCGCUCUGACCAU
GAACUGACAGGUCAGCUGUCCUGAAAGCUGACAUGUCAUCGAUCUCGAACUCGUCUGACUCUGUGACGGCUGAA
CUUGUGACCUGUUACGUGUCAUCGUGACUUACAGCUCGACCAGGAUGAGCACCACGUUCUGACCUCUUGUGAAC
AGCUCCUCACUCUUGCUACCC

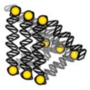

9Kt-33-Tri-long: GGGUAGCAAGAGUGAGGAGCUGUUCACAAGAGGCAUGAUGAACGUGGUGCUCAUCCUGGUCGAGCUGUAAGCGU
GAUCGAUGACACGUAACAGGUCACAAGUUCAGCCGCAUGAUCAGAGUCAGACGAGUUCGAGAUCGAUGACAUGC
AUGAUGCUUUCAGGACAGCUGACCUGUCAGUUCAUGGCGUGAUGAGCGCACCACUUGCAAGCUGCCAGUGCAAU
GCAUGAUCUCGCACCACUCUCGUGACCACGCUGAACUGGCGUGAUCAACAGCGUGCAGUGCUUCAGUUGCUACC
AUGCGUGAUGACAACCACAUGAAGCAGCACGACUUCUUCCGCAUGAUGGUAGUCAUCCAUGUCCGAAGGCUACG
UUCCC

9Kt-33-Tri-short: GGGAACGUAGCCUUCGGACAUGGAUGACUACCUGACGGAAGAAGUCGUGCUGCUUCAUGUGGUUGUCUGACAUG
GUAGCAACUGAAGCACUGCACGCUGUUGUGACCAGUUCAGCGUGGUCACGAGAGUGGUGCGAGUGACAUUGCAC
UGGCAGCUUGCAAGUGGUGCGCUCUGACCAUGAACUGACAGGUCAGCUGUCCUGAAAGCUGACAUGUCAUCGAU
CUCGAACUCGUCUGACUCUGUGACGGCUGAACUUGUGACCUGUUACGUGUCAUCGUGACUUACAGCUCGACCAG
GAUGAGCACCACGUUCUGACCUCUUGUGAACAGCUCCUCACUCUUGCUACCC

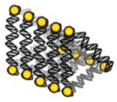

14Kt-33-Tri-long: GGGUAGCAAGAGUGAGGAGCUGUUCACAAGAGGCAUGAUGAACGUGGUGCUCAUCCUGGUCGAGCUGUAAGCGU
GAUCGAUGACACGUAACAGGUCACAAGUUCAGCCGCAUGAUCAGAGUCAGACGAGUUCGAGAUCGAUGACAUGC
AUGAUGCUUUCAGGACAGCUGACCUGUCAGUUCAUGGCGUGAUGAGCGCACCACUUGCAAGCUGCCAGUGCAAU
GCAUGAUCUCGCACCACUCUCGUGACCACGCUGAACUGGCGUGAUCAACAGCGUGCAGUGCUUCAGUUGCUACC
AUGCGUGAUGACAACCACAUGAAGCAGCACGACUUCUUCCGCAUGAUGGUAGUCAUCCAUGUCCGAAGGCUACG
UUCAGCAUGAUCACCGAGCACACCAUCUUCUUCAAGGACGAAGCGUGAUGUUCGCAACUACAAGACACGUGCAG
AGGUGGGCAUGAUCCAUGUUCGAGUACGACACCCUGGUGAACUAGCAUGAUGUGCAUCGAGCUGAAGUACAUCG
ACUUCAAUGCGUGAUCUGAAGGACGGCAACAUCCUGUAUCACAACCC

14Kt-33-Tri-short: GGGUUGUGAUACAGGAUGUUGCCGUCCUUCAGUGACAUUGAAGUCGAUGUACUUCAGCUCGAUGCACUGACUAG
UUCACCAGGGUGUCGUACUCGAACAUGGUGACCCACCUCUGCACGUGUCUUGUAGUUGCGAACUGACUUCGUCC
UUGAAGAAGAUGGUGUGCUCGGUGUGACUGAACGUAGCCUUCGGACAUGGAUGACUACCUGACGGAAGAAGUCG
UGCUGCUUCAUGUGGUUGUCUGACAUGGUAGCAACUGAAGCACUGCACGCUGUUGUGACCAGUUCAGCGUGGUC
ACGAGAGUGGUGCGAGUGACAUUGCACUGGCAGCUUGCAAGUGGUGCGCUCUGACCAUGAACUGACAGGUCAGC
UGUCCUGAAAGCUGACAUGUCAUCGAUCUCGAACUCGUCUGACUCUGUGACGGCUGAACUUGUGACCUGUUACG
UGUCAUCGUGACUUACAGCUCGACCAGGAUGAGCACCACGUUCUGACCUCUUGUGAACAGCUCCUCACUCUUGC
UACCC

Supplementary Figure 22

RNA sequences of 1, 3, 6, 9, and 14Kt-33-Tri RNA nanostructures. All RNA nanostructures consist of two RNA strands: "long strand" (green) and "short strand" (blue). The K-turn motifs are shown in red and black letters.

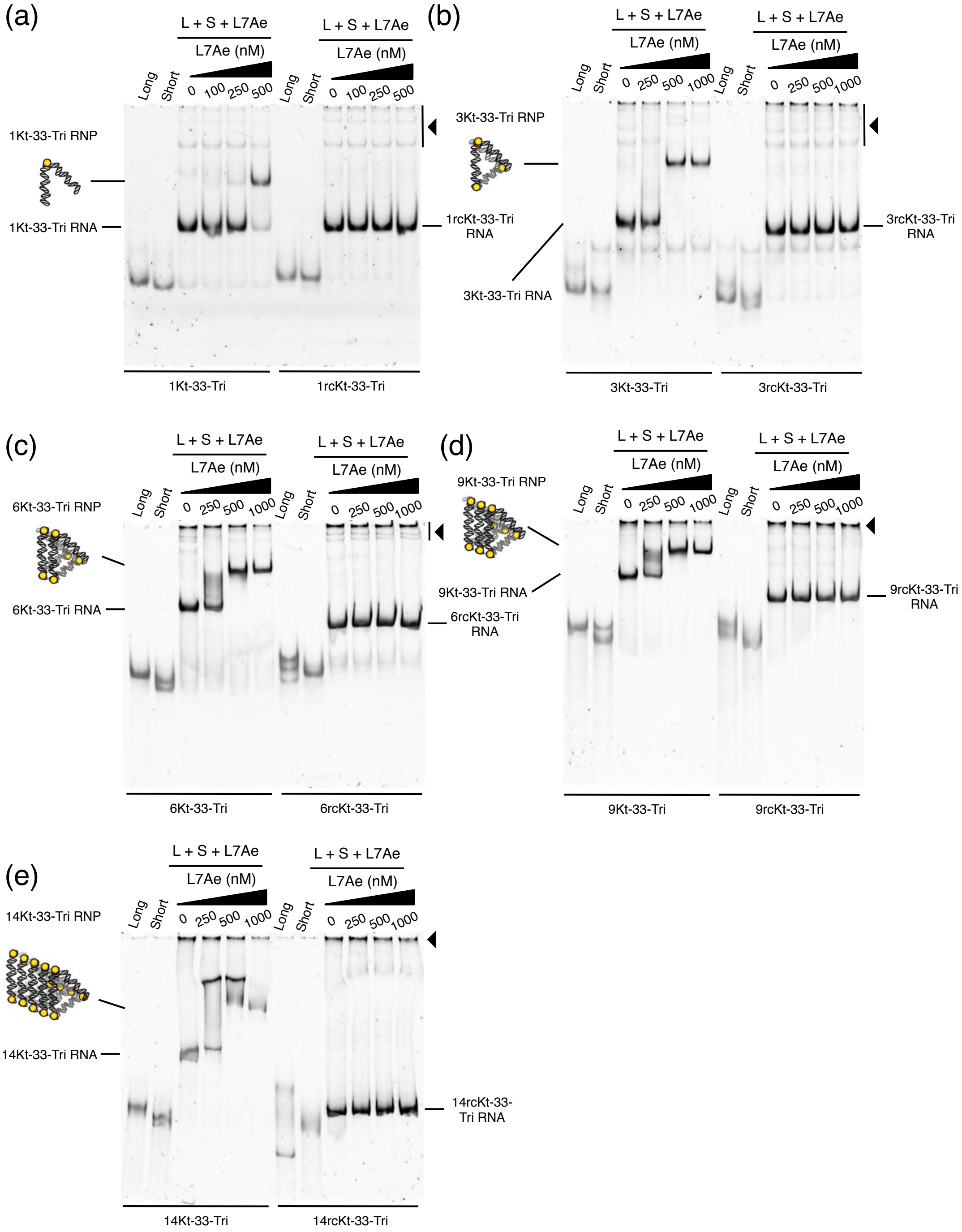

Supplementary Figure 23

EMSA to confirm interactions between the RNA nanostructures and L7Ae. (a) 1Kt-33-Tri and 1rcKt-33-Tri. (b) 3Kt-33-Tri and 3rcKt-33-Tri. (c) 6Kt-33-Tri and 6rcKt-33-Tri. (d) 9Kt-33-Tri and 9rcKt-33-Tri. (e) 14Kt-33-Tri and 14rcKt-33-Tri. Higher order bands (black arrowheads) indicate heterogeneous oligomers composed of L- and S-RNA strands. Concentrations of long and short RNAs: each (a)-(d) 50 nM and (e) 20 nM.

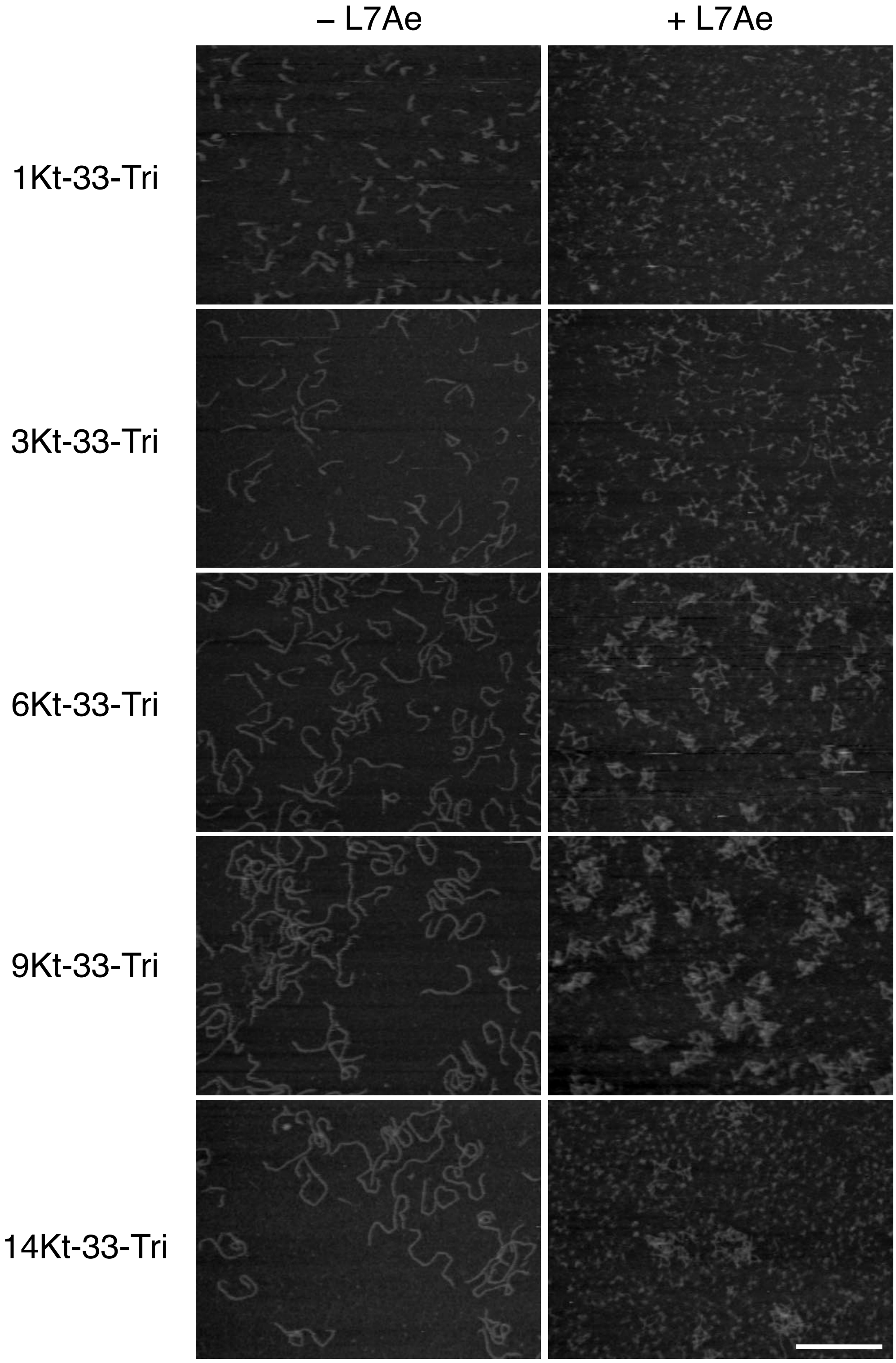

Supplementary Figure 24

AFM images of 1, 3, 6, 9, and 14Kt-33-Tri in the absence (left) and presence (right) of L7Ae. Scale bars: 100 nm.

1rcKt-33-Tri-long: GGGUUGUGAUACAGGAUGUUGCCGUCCUUCAGAUCACGCAUUGAAGUCGAUGUACUUCAGCUCGAUGCCCC

1rcKt-33-Tri-short: GGGGCAUCGAGCUGAAGUACAUCGACUUCAAUGUCACUGAAGGACGGCAACAUCCUGUAUCACAACCC

3rcKt-33-Tri-long: GGGUUGUGAUACAGGAUGUUGCCGUCCUUCAGAUCACGCAUUGAAGUCGAUGUACUUCAGCUCGAUGCACAUCA
UGCUAGUUCACCAGGGUGUCGUACUCGAACAUGGAUCAUGCCCACCUCUGCACGUGUCUUGUAGUUGCGACCC

3rcKt-33-Tri-short: GGGUCGCAACUACAAGACACGUGCAGAGGUGGGUCACCAUGUUCGAGUACGACACCCUGGUGAACUAGUCAGUG
CAUCGAGCUGAAGUACAUCGACUUCAAUGUCACUGAAGGACGGCAACAUCCUGUAUCACAACCC

6rcKt-33-Tri-long: GGGUUGUGAUACAGGAUGUUGCCGUCCUUCAGAUCACGCAUUGAAGUCGAUGUACUUCAGCUCGAUGCACAUCA
UGCUAGUUCACCAGGGUGUCGUACUCGAACAUGGAUCAUGCCCACCUCUGCACGUGUCUUGUAGUUGCGAACAU
CACGCUUCGUCCUUGAAGAAGAUGGUGUGCUCGGUGAUCAUGCUGAACGUAGCCUUCGGACAUGGAUGACUACC
AUCAUGCGGAAGAAGUCGUGCUGCUUCAUGUGGUUGCCC

6rcKt-33-Tri-short: GGGCAACCACAUGAAGCAGCACGACUUCUUCCGUCAGGUAGUCAUCCAUGUCCGAAGGCUACGUUCAGUCACAC
CGAGCACACCAUCUUCUUCAAGGACGAAGUCAGUUCGCAACUACAAGACACGUGCAGAGGUGGGUCACCAUGUU
CGAGUACGACACCCUGGUGAACUAGUCAGUGCAUCGAGCUGAAGUACAUCGACUUCAAUGUCACUGAAGGACGG
CAACAUCCUGUAUCACAACCC

9rcKt-33-Tri-long: GGGUUGUGAUACAGGAUGUUGCCGUCCUUCAGAUCACGCAUUGAAGUCGAUGUACUUCAGCUCGAUGCACAUCA
UGCUAGUUCACCAGGGUGUCGUACUCGAACAUGGAUCAUGCCCACCUCUGCACGUGUCUUGUAGUUGCGAACAU
CACGCUUCGUCCUUGAAGAAGAUGGUGUGCUCGGUGAUCAUGCUGAACGUAGCCUUCGGACAUGGAUGACUACC
AUCAUGCGGAAGAAGUCGUGCUGCUUCAUGUGGUUGUCAUCACGCAUGGUAGCAACUGAAGCACUGCACGCUGU
UGAUCACGCCAGUUCAGCGUGGUCACGAGAGUGGUGCGAGAUCAUGCAUUGCACUGGCAGCUUGCAAGUGGUGC
GCCCC

9rcKt-33-Tri-short: GGGGCGCACCACUUGCAAGCUGCCAGUGCAAUGUCACUCGCACCACUCUCGUGACCACGCUGAACUGGUCACAA
CAGCGUGCAGUGCUUCAGUUGCUACCAUGUCAGACAACCACAUGAAGCAGCACGACUUCUUCCGUCAGGUAGUC
AUCCAUGUCCGAAGGCUACGUUCAGUCACACCGAGCACACCAUCUUCUUCAAGGACGAAGUCAGUUCGCAACUA
CAAGACACGUGCAGAGGUGGGUCACCAUGUUCGAGUACGACACCCUGGUGAACUAGUCAGUGCAUCGAGCUGAA
GUACAUCGACUUCAAUGUCACUGAAGGACGGCAACAUCCUGUAUCACAACCC

14rcKt-33-Tri-long: GGGUUGUGAUACAGGAUGUUGCCGUCCUUCAGAUCACGCAUUGAAGUCGAUGUACUUCAGCUCGAUGCACAUCA
UGCUAGUUCACCAGGGUGUCGUACUCGAACAUGGAUCAUGCCCACCUCUGCACGUGUCUUGUAGUUGCGAACAU
CACGCUUCGUCCUUGAAGAAGAUGGUGUGCUCGGUGAUCAUGCUGAACGUAGCCUUCGGACAUGGAUGACUACC
AUCAUGCGGAAGAAGUCGUGCUGCUUCAUGUGGUUGUCAUCACGCAUGGUAGCAACUGAAGCACUGCACGCUGU
UGAUCACGCCAGUUCAGCGUGGUCACGAGAGUGGUGCGAGAUCAUGCAUUGCACUGGCAGCUUGCAAGUGGUGC
GCUCAUCACGCCAUGAACUGACAGGUCAGCUGUCCUGAAAGCAUCAUGCAUGUCAUCGAUCUCGAACUCGUCUG
ACUCUGAUCAUGCGGCUGAACUUGUGACCUGUUACGUGUCAUCGAUCACGCUUACAGCUCGACCAGGAUGAGCA
CCACGUUCAUCAUGCCUCUUGUGAACAGCUCCUCACUCUUGCUACCC

14rcKt-33-Tri-short: GGGUAGCAAGAGUGAGGAGCUGUUCACAAGAGGUCAGAACGUGGUGCUCAUCCUGGUCGAGCUGUAAGUCACGA
UGACACGUAACAGGUCACAAGUUCAGCCGUCACAGAGUCAGACGAGUUCGAGAUCGAUGACAUGUCAGCUUUCA
GGACAGCUGACCUGUCAGUUCAUGGUCAGAGCGCACCACUUGCAAGCUGCCAGUGCAAUGUCACUCGCACCACU
CUCGUGACCACGCUGAACUGGUCACAACAGCGUGCAGUGCUUCAGUUGCUACCAUGUCAGACAACCACAUGAAG
CAGCACGACUUCUUCCGUCAGGUAGUCAUCCAUGUCCGAAGGCUACGUUCAGUCACACCGAGCACACCAUCUUC
UUCAAGGACGAAGUCAGUUCGCAACUACAAGACACGUGCAGAGGUGGGUCACCAUGUUCGAGUACGACACCCUG
GUGAACUAGUCAGUGCAUCGAGCUGAAGUACAUCGACUUCAAUGUCACUGAAGGACGGCAACAUCCUGUAUCAC
AACCC

Supplementary Figure 25

RNA sequences of 1, 3, 6, 9, and 14rcKt-33-Tri RNA nanostructures. All RNA nanostructures consist of two RNA strands: “long strand” (green) and “short strand” (blue). The reverse-complementary K-turn motifs are shown in red and black letters.

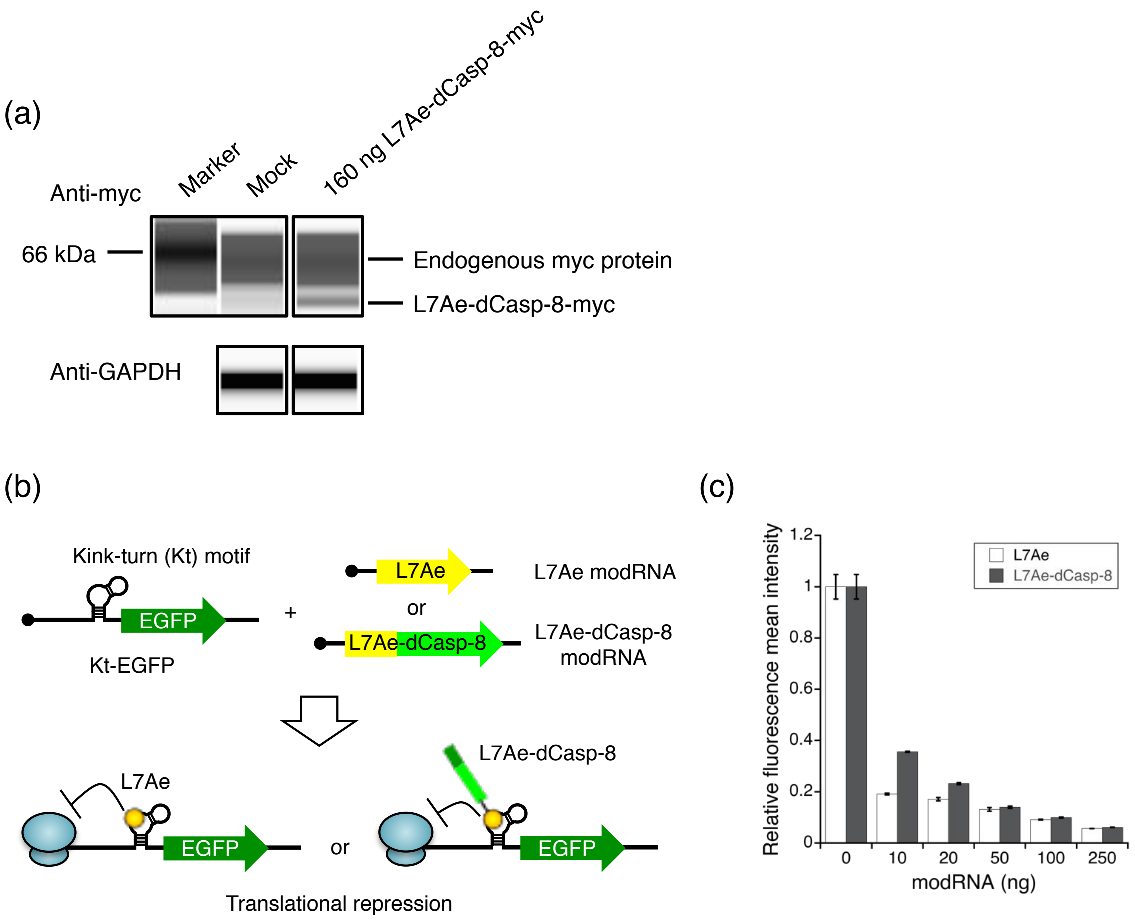

Supplementary Figure 26

(a) Virtual blot-like image of capillary electrophoresis of L7Ae-dCasp-8-myc using anti-myc antibody. GAPDH was detected using anti-GAPDH antibody and used as a control. (b) Schematic representation and (c) flow cytometric analysis of translational regulation by L7Ae and L7Ae-dCasp-8. The data are presented as the mean ± SD (*n* = 3).

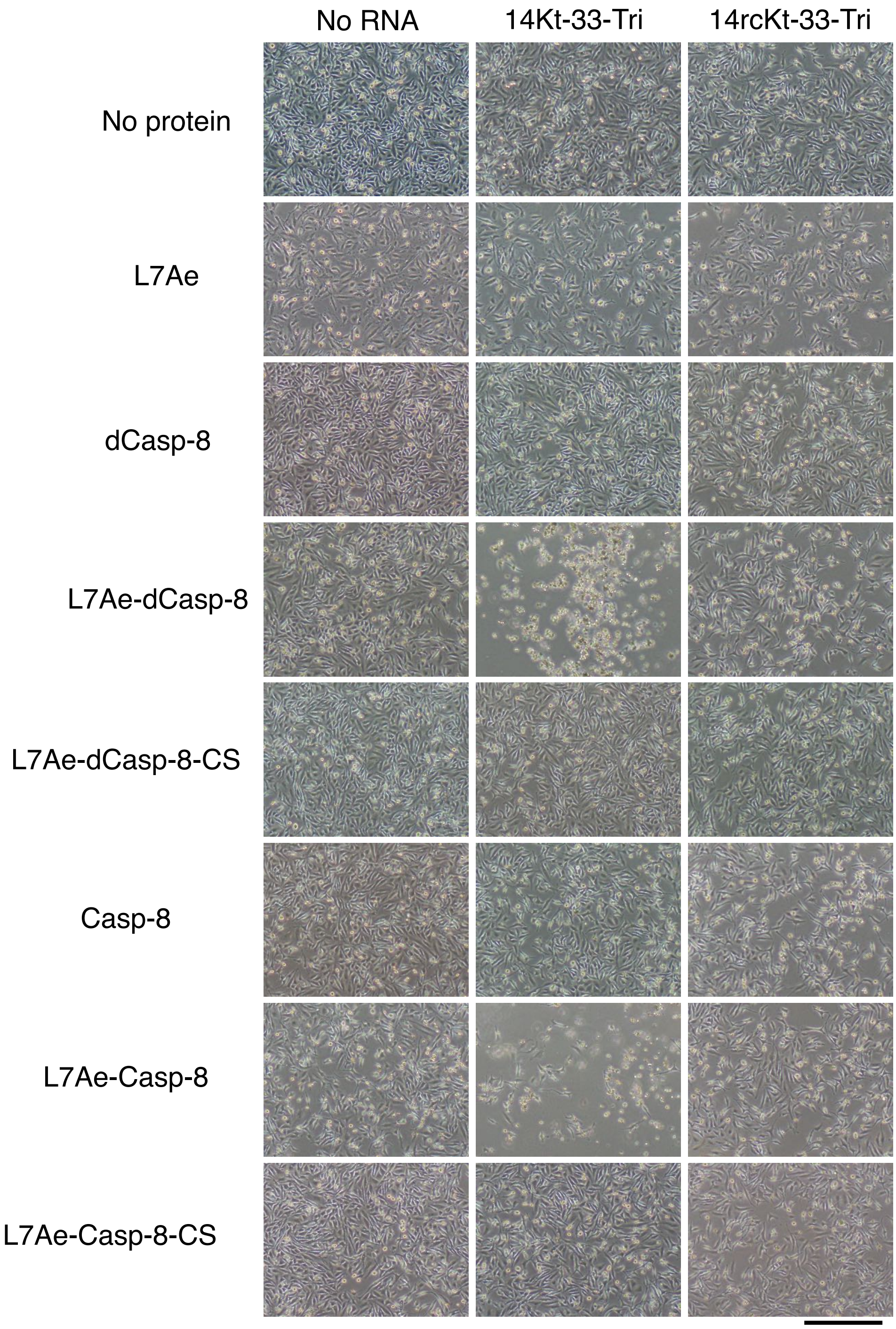

Supplementary Figure 27

Microscopic images of cells after 24 hours of transfection with RNA nanostructures and modRNAs encoding protein variants. Scale bar: 500 µm.

9pre-let7d-33-long: GGGUAGCAAGAGUGAGGAGCUGUUCACAAGAGGUU**AUAGUUUUAGGGCAGGGAUUUUGCCCACAAGGAGGUAAC**
UAUUUGAACGUGGUGCUCAUCCUGGUCGAGCUGUAAGUU**GAAGCCUUAGGGCAGGGAUUUUGCCCACAAGGAGG**
UGGCUUCUUCGAUGACACGUAACAGGUCACAAGUUCAGCCGUU**GGAUACUUAGGGCAGGGAUUUUGCCCACAAG**
GAGGUGUAUCCUUCAGAGUCAGACGAGUUCGAGAUCGAUGACAUGUU**AGACUGUUAGGGCAGGGAUUUUGCCCA**
CAAGGAGGUCAGUCUUUGCUUUCAGGACAGCUGACCUGUCAGUUCAUGGUU**GCAGACUUAGGGCAGGGAUUUUG**
CCCACAAGGAGGUGUCUGCUUGAGCGCACCACUUGCAAGCUGCCAGUGCAAUGUU**AGCACCUUAGGGCAGGGAU**
UUUGCCCACAAGGAGGUGGUGCUUUCUCGCACCACUCUCGUGACCACGCUGAACUGGUU**GUGACCUUAGGGCAG**
GGAUUUUGCCCACAAGGAGGUGGUCACUUCAACAGCGUGCAGUGCUUCAGUUGCUACCAUGUU**AGGCAGUUAGG**
GCAGGGAUUUUGCCCACAAGGAGGUCUGCCUUUGACAACCACAUGAAGCAGCACGACUUCUUCCGUU**GUAGGCU**
UAGGGCAGGGAUUUUGCCCACAAGGAGGUGCCUACUUGGUAGUCAUCCAUGUCCGAAGGCUACGUUCCC

Supplementary Figure 28

RNA sequences of 9pre-let7d-33 RNA nanostructures. 9pre-let7d-33 RNA nanostructure consist of two RNA strands: 9pre-let7d-33-long and 9Kt-33-Tri-short (shown in Fig. S22). The pre-let7d motifs are shown in red letters.

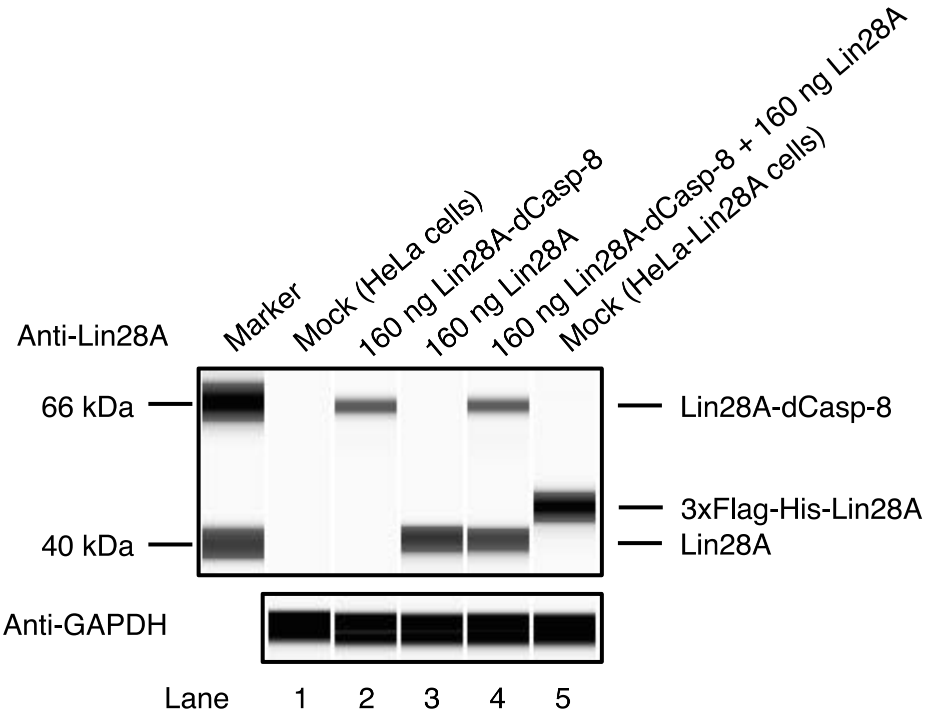

Supplementary Figure 29

Virtual blot-like image of capillary electrophoresis of Lin28A and Lin28A-dCasp-8 using anti-Lin28A antibody. Cell lysates after 24 hours of transfection with Lin28A-dCasp-8 (lane 2), Lin28A (lane 3), and both Lin28A-dCasp-8 and Lin28A (lane 4). Cell lysates of HeLa-Lin28A cells that stably express Lin28A (lane 5). GAPDH was detected using anti-GAPDH antibody and used as a control.

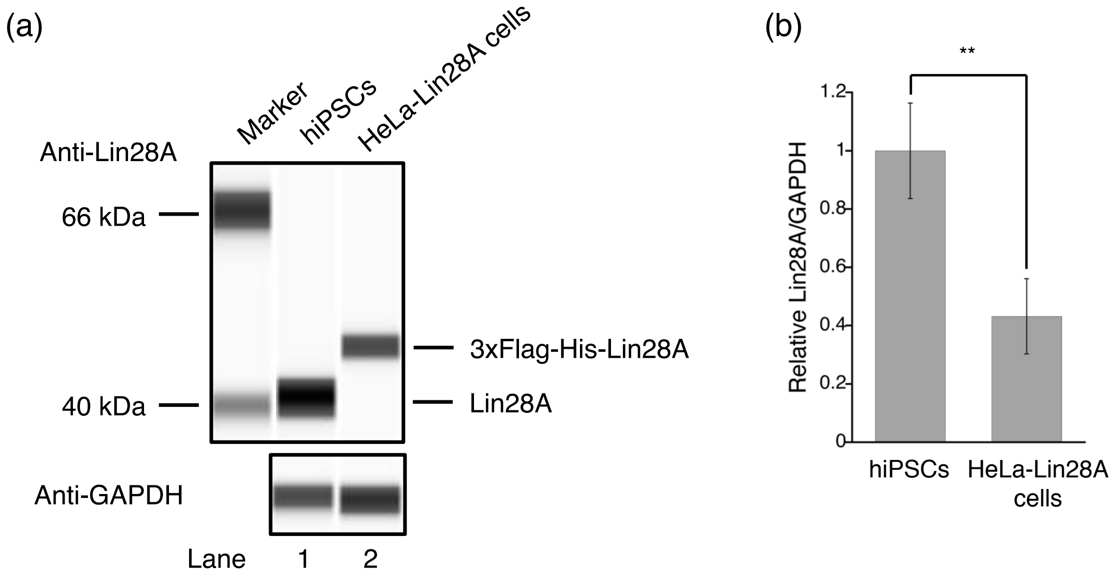

Supplementary Figure 30

(a) Virtual blot-like image of capillary electrophoresis of Lin28A using anti-Lin28A antibody. Cell lysates of hiPSCs (lane 1) and HeLa-Lin28A cells (lane 2). GAPDH was detected using anti-GAPDH antibody and used as a control. (b) Relative Lin28A/GAPDH in hiPSCs and HeLa-Lin28A cells. The data are presented as the mean ± SD (n = 4). $^{**}P < 0.01$ (Welch's t-test).

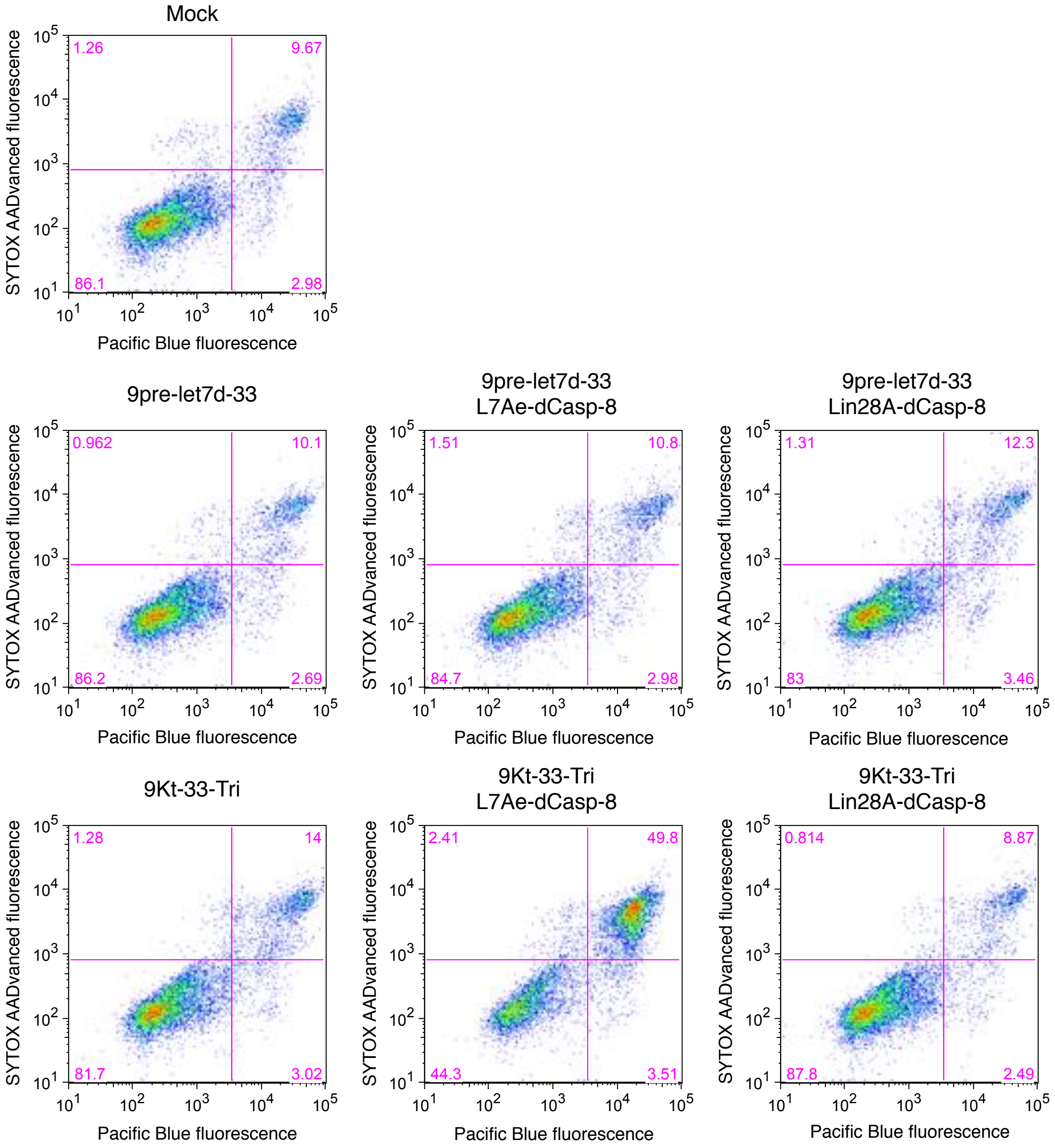

Supplementary Figure 31

Flow cytometric analysis for cell death assay of hiPSCs using Lin28A-responsive RNA scaffold. Two-dimensional plots (Pacific Blue vs. SYTOX AADvanced) after 24 hour transfection with RNA scaffolds (9pre-let7d-33 or 9Kt-33-Tri) and dCasp-8-fused proteins (Lin28A-dCasp-8 or L7Ae-dCasp-8).

14Kt-27,28-Zig-long: GGGUAGCAAGAGUGAGGAGUCCAAGAGGCAUGAUGAACGUGGUGCUCAUCCUGCUGUAAGCGUGAUCGAUGAAA
CAGGUCACAAGUUCAGCCGCAUGAUCAGAGGAGUUCGAGAUCGAUGACAUGCAUGAUGCUUUCAGGACAGCUGA
CCUGUCAUGGCGUGAUGAGGCAUGCAAGCUGCCAGUGCAAUGCAUGAUCUCGCACCAACCUCGUGACCGAACUG
GCGUGAUCAACAGCGUGCUUCGUUGCUACCAUGCGUGAUGAACGUAUAGCAGCACGACUUCUUCCGCAUGAUGG
UUCCAUUCCGAAGGCUACGUUCAGCAUGAUCACCGAGCACACGCAUCAAGGACGAAGCGUGAUGUUCGCAACUA
CACGUGCAGAGUGGGCAUGAUCCAUGUUCGACAUCCCUGGUGAACUAGCAUGAUGUGCAUCGAGCUGCAUCGCU
UCAAUGCGUGAUCUGAAGGACGUCCUCGUAUCACAACCC

14Kt-27,28-Zig-short: GGGUUGUGAUACGAGGACGUCCUUCAGUGACAUUGAAGCGAUGCAGCUCGAUGCACUGACUAGUUCACCAGGGA
UGUCGAACAUGGUGACCCACUCUGCACGUGUAGUUGCGAACUGACUUCGUCCUUGAUGCGUGUGCUCGGUGUGA
CUGAACGUAGCCUUCGGAAUGGAACCUGACGGAAGAAGUCGUGCUGCUAUACGUUCUGACAUGGUAGCAACGAA
GCACGCUGUUGUGACCAGUUCGGUCACGAGGUUGGUGCGAGUGACAUUGCACUGGCAGCUUGCAUGCCUCUGAC
CAUGACAGGUCAGCUGUCCUGAAAGCUGACAUGUCAUCGAUCUCGAACUCCUCUGUGACGGCUGAACUUGUGAC
CUGUUUCAUCGUGACUUACAGCAGGAUGAGCACCACGUUCUGACCUCUUGGACUCCUCACUCUUGCUACCC

14rcKt-27,28-Zig-long: GGGUUGUGAUACGAGGACGUCCUUCAGAUCACGCAUUGAAGCGAUGCAGCUCGAUGCACAUCAUGCUAGUUCAC
CAGGGAUGUCGAACAUGGAUCAUGCCCACUCUGCACGUGUAGUUGCGAACAUCACGCUUCGUCCUUGAUGCGUG
UGCUCGGUGAUCAUGCUGAACGUAGCCUUCGGAAUGGAACCAUCAUGCGGAAGAAGUCGUGCUGCUAUACGUUC
AUCACGCAUGGUAGCAACGAAGCACGCUGUUGAUCACGCCAGUUCGGUCACGAGGUUGGUGCGAGAUCAUGCAU
UGCACUGGCAGCUUGCAUGCCUCAUCACGCCAUGACAGGUCAGCUGUCCUGAAAGCAUCAUGCAUGUCAUCGAU
CUCGAACUCCUCUGAUCAUGCGGCUGAACUUGUGACCUGUUUCAUCGAUCACGCUUACAGCAGGAUGAGCACCA
CGUUCAUCAUGCCUCUUGGACUCCUCACUCUUGCUACCC

14rcKt-27,28-Zig-short: GGGUAGCAAGAGUGAGGAGUCCAAGAGGUCAGAACGUGGUGCUCAUCCUGCUGUAAGUCACGAUGAAACAGGUC
ACAAGUUCAGCCGUCACAGAGGAGUUCGAGAUCGAUGACAUGUCAGCUUUCAGGACAGCUGACCUGUCAUGGUC
AGAGGCAUGCAAGCUGCCAGUGCAAUGUCACUCGCACCAACCUCGUGACCGAACUGGUCACAACAGCGUGCUUC
GUUGCUACCAUGUCAGAACGUAUAGCAGCACGACUUCUUCCGUCAGGUUCCAUUCCGAAGGCUACGUUCAGUCA
CACCGAGCACACGCAUCAAGGACGAAGUCAGUUCGCAACUACACGUGCAGAGUGGGUCACCAUGUUCGACAUCC
CUGGUGAACUAGUCAGUGCAUCGAGCUGCAUCGCUUCAAUGUCACUGAAGGACGUCCUCGUAUCACAACCC

Supplementary Figure 32

RNA sequences of 14Kt-27,28-Zig and 14rcKt-27,28-Zig. All RNA nanostructures consist of two RNA strands: “long strand” (green) and “short strand” (blue). The K-turn motifs (Kt) and reverse-complementary K-turn motifs (rcKt) are shown in red and black letters.

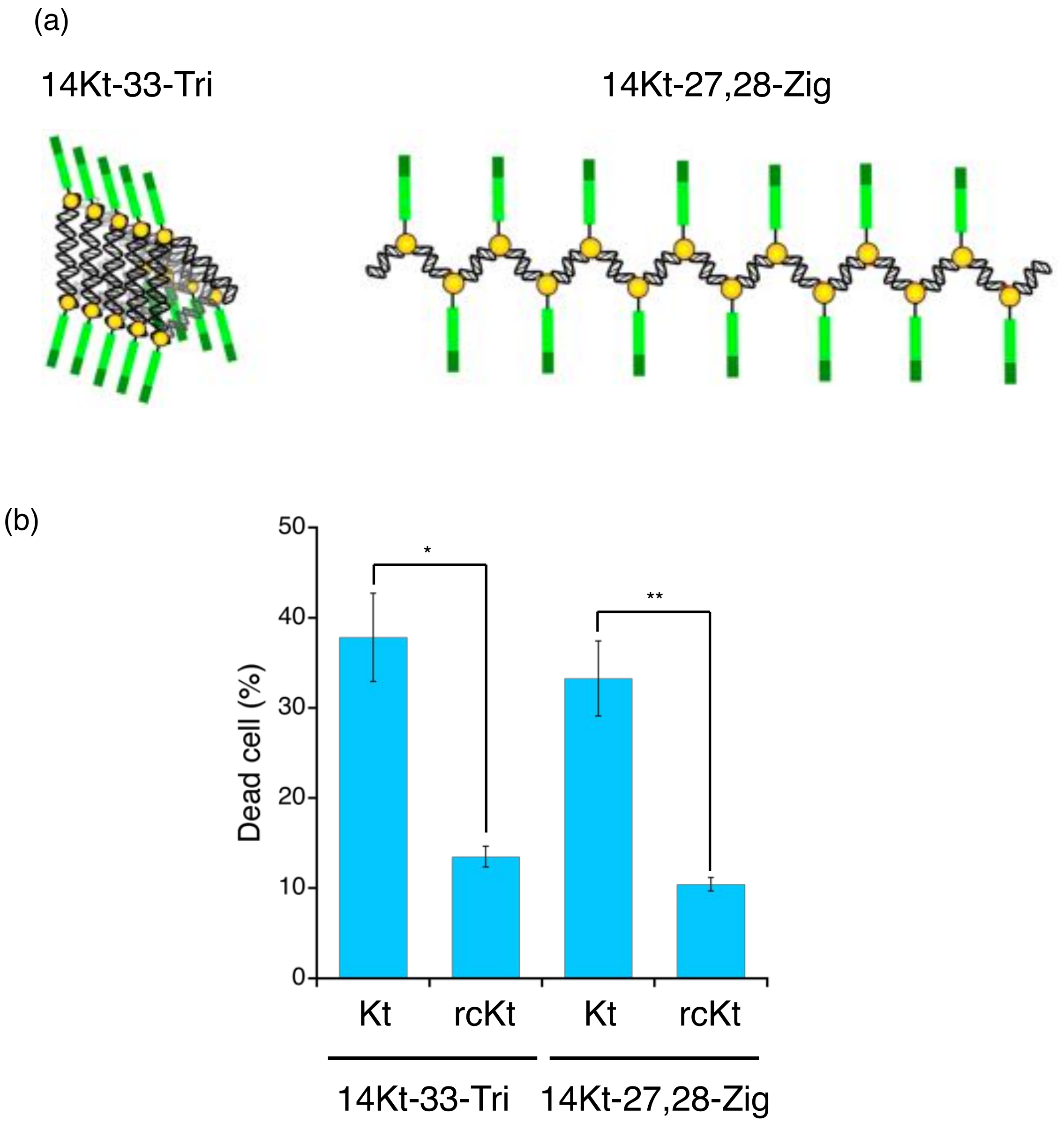

Supplementary Figure 33

(a) Schematic illustration of 14Kt-33-Tri and 14Kt-27,28-Zig. (b) Cell death induced by a specific interaction between 14Kt-33-Tri or 14Kt-27,28-Zig and L7Ae-dCasp-8. RNA nanostructures and modRNAs were co-transfected into HeLa cells. The cells were stained with Pacific Blue Annexin V and analysed using flow cytometry. Dead cells were counted by considering Pacific Blue-positive cells to be dead. Adherent cells were collected after washing cell-attached dishes with PBS and then analyzed by FACS. The data are presented as the mean ± SD (n = 3). *$P < 0.05$, **$P < 0.01$ (Welch's t-test).

(a)

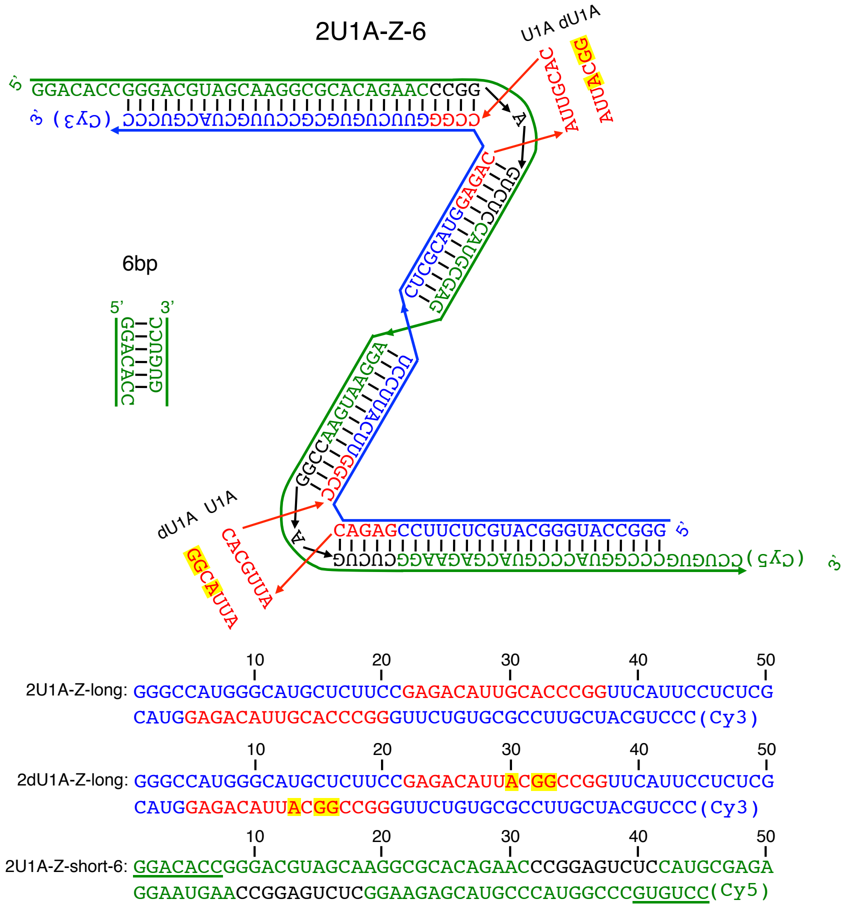

(b)

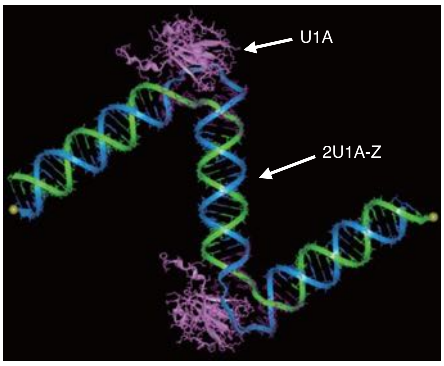

Supplementary Figure 34

(a) 2D structures and RNA sequence of 2U1A-Z-6 RNA nanostructures with a short RNA duplex of 6 bps. The RNA nanostructures consist of two RNA strands: "long strand" (blue) and "short RNA duplex-conjugated short strand" (green). The two U1A-binding motifs are shown in red and black letters. The sequence of the short RNA duplexes is underlined. Letters highlighted in yellow indicate the mutated nucleobases in the U1A-binding motifs. (b) Three-dimensional modelling of the 2U1A-Z nanostructure.

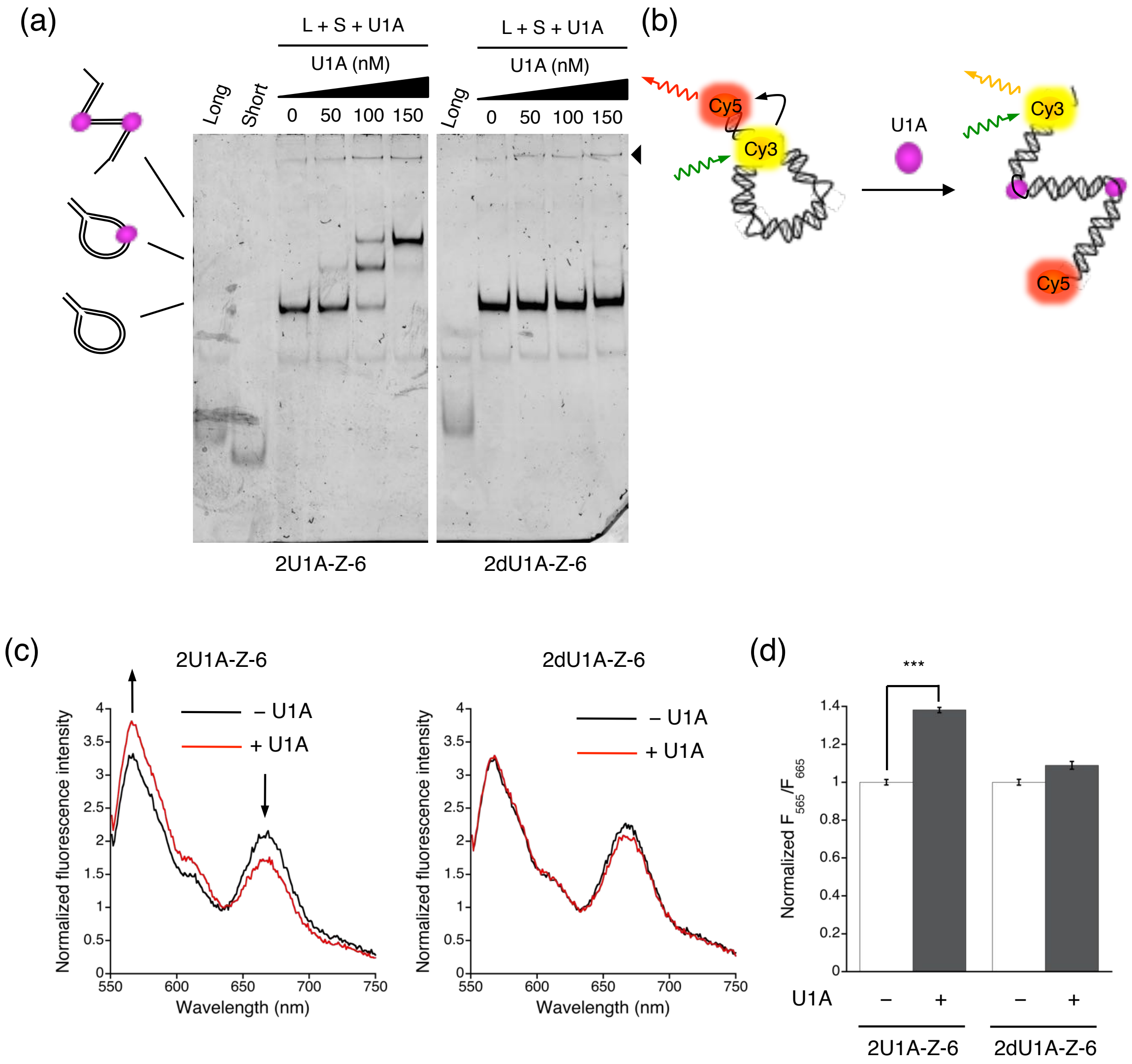

Supplementary Figure 35

(a) EMSA to confirm RNP interactions between 2U1A-Z-6 (left) or 2dU1A-Z-6 (right) nanostructures and U1A. Higher order bands (black arrowheads) indicate heterogeneous oligomers composed of L- and S-RNA strands. Concentrations of long and short RNAs: each 50 nM. (b) Schematic representation of the U1A-responsive RNA nanodevice that produces a change in FRET signal upon U1A binding. (c) Fluorescence spectra of 2U1A-Z-6 (left) and 2dU1A-Z-6 (right) in the absence (black) and presence (red) of U1A. (d) Fold change of F_{565}/F_{665} in the absence (white) and presence (gray) of U1A. The data are presented as the mean ± SD (n = 3). ***$P < 0.001$ (Welch's t-test).

Supplementary Table 1. Primer sequences used in the PCRs for RNA nanostructures

**2Kt-33-Tri-long-Fwd**	GCTAATACGACTCACTATAGGGACGTAGCAAGGCGCAAAGGCCTGTAATCGGCGTGATGAGCCATGCGAGGAGGAAATG
**2Kt-33-Tri-long-Rev**	GGCCATGGGCATGCTCTTCCCGTAGAGGCTCATCACGCCATTGGACTTCATTTCCTCCTCGCATGGCTCATCAC
**2Kt-33-Tri-long+C-Rev**	GGGCCATGGGCATGCTCTTCCCGTAGAGGCTCATCACGCCATTGGACTTCATTTCCTCCTCGCATGGCTCATCAC
**2dKt-33-Tri-long-Fwd**	GCTAATACGACTCACTATAGGGACGTAGCAAGGCGCAAAGGCCTGTAATCGGCGGCCTGAGCCATGCGAGGAGGAAATG
**2dKt-33-Tri-long-Rev**	GGCCATGGGCATGCTCTTCCCGTAGAGGCTCAGGCCGCCATTGGACTTCATTTCCTCCTCGCATGGCTCAG
**2Kt-33-Tri-short-Fwd**	GCTAATACGACTCACTATAGGGCCATGGGCATGCTCTTCCCGTAGAGGCTCTGACCATTGGACTTCATTTCCTC
**2Kt-33-Tri-short-Rev**	GGACGTAGCAAGGCGCAAAGGCCTGTAATCGGTCAGAGCCATGCGAGGAGGAAATGAAGTCCAATGGTCAG
**2Kt-28-Z-long-Fwd**	GCTAATACGACTCACTATAGGGACGTAGCAAGGCGCAAAGGCCTGGGCGTGATGAGCCATGCGAGGAGGAAATGAAGTG
**2Kt-28-Z-long-Rev**	GGGCCATGGGCATGCTCTTCCCGGCTCATCACGCCACTTCATTTCCTCCTCGCATG
**2Kt-28-Z-long-C-Rev**	GGCCATGGGCATGCTCTTCCCGGCTCATCACGCCACTTCATTTCCTCCTCGCATG
**2dKt-28-Z-long-Fwd**	GCTAATACGACTCACTATAGGGACGTAGCAAGGCGCAAAGGCCTGGGCGGCCTGAGCCATGCGAGGAGGAAATGAAGTG
**2dKt-28-Z-long-Rev**	GGGCCATGGGCATGCTCTTCCCGGCTCAGGCCGCCACTTCATTTCCTCCTCGCATG
**2dKt-28-Z-long-C-Rev**	GGCCATGGGCATGCTCTTCCCGGCTCAGGCCGCCACTTCATTTCCTCCTCGCATG
**2Kt-28-Z-short-Fwd**	GCTAATACGACTCACTATAGGGCCATGGGCATGCTCTTCCCGGCTCTGACCACTTCATTTCCTCCTCGCATGG
**2Kt-28-Z-short-Rev**	GGGACGTAGCAAGGCGCAAAGGCCTGGGTCAGAGCCATGCGAGGAGGAAATGAAGTG
**2Kt-28-Z-short-6'-Fwd**	GCTAATACGACTCACTATAGGACGCGGGCCATGGGCATGCTCTTCCCGGCTCTGACCACTTCATTTCCTCCTCGCATG
**2Kt-28-Z-short-5-C-Rev**	GACGGGGACGTAGCAAGGCGCAAAGGCCTGGGTCAGAGCCATGCGAGGAGGAAATGAAGTGGTC
**2Kt-28-Z-short-6'-C-Rev**	GACGCGGGACGTAGCAAGGCGCAAAGGCCTGGGTCAGAGCCATGCGAGGAGGAAATGAAGTGGTC
**2Kt-28-Z-short-7-Fwd**	GCTAATACGACTCACTATAGGACACCGGGCCATGGGCATGCTCTTCCCGGCTCTGACCACTTCATTTCCTCCTCGCATG
**2Kt-28-Z-short-6-C-Rev**	GACACGGGACGTAGCAAGGCGCAAAGGCCTGGGTCAGAGCCATGCGAGGAGGAAATGAAGTGGTC
**2Kt-28-Z-short-7-C-Rev**	GACACCGGGACGTAGCAAGGCGCAAAGGCCTGGGTCAGAGCCATGCGAGGAGGAAATGAAGTGGTC
**Tri-MGA-ON-short-1st-Fwd**	GGGCCATGGGCATGCTCTTCCCGTAGAGGCTCTGACCATTGGACTTCATTTCCTC
**Z-MGA-OFF-short-1st-Fwd**	GGGCCATGGGCATGCTCTTCCCGGCTCTGACCACTTCATTTCCTCCTCGCATGG
**short-MGA-original-U0-Fwd**	GCTAATACGACTCACTATAGGAGGTAACGAATGGCGGGCCATGGGCATGCTCTTCC
**short-MGA-original-U0-Rev**	GGAGTCGGGCGGGACGTAGCAAGGCGCAAAG
**short-MGA-original-U2-Fwd**	GCTAATACGACTCACTATAGGAGGTAACGAATGGCTTGGGCCATGGGCATGCTCTTCC
**short-MGA-original-U2-Rev**	GGAGTCGGGCAAGGGACGTAGCAAGGCGCAAAG
**short-MGA-original-U4-Fwd**	GCTAATACGACTCACTATAGGAGGTAACGAATGGCTTTTGGGCCATGGGCATGCTCTTCC
**short-MGA-original-U4-Rev:**	GGAGTCGGGCAAAAGGGACGTAGCAAGGCGCAAAG
**Tri-MGA-ON-stem A-Fwd**	GCTAATACGACTCACTATAGGAGGTAACGAATGGGGGCCATGGGCATGCTCTTCC
**Tri-MGA-ON-stem A-Rev**	GGAGTCGGGGGGACGTAGCAAGGCGCAAAG
**Tri-MGA-ON-stem B-Fwd**	GCTAATACGACTCACTATAGTAGGTAACGAATGGGGGCCATGGGCATGCTCTTCC
**Tri-MGA-ON-stem B-Rev**	GTAGTCGGGGGGACGTAGCAAGGCGCAAAG
**Tri-MGA-ON-stem C-Fwd**	GCTAATACGACTCACTATAGTGGTAACGAATGGCGGGCCATGGGCATGCTCTTCC
**Tri-MGA-ON-stem C-Rev**	GTGTCGGGCGGGACGTAGCAAGGCGCAAAG
**Z-MGA-OFF-stem D-Fwd**	GCTAATACGACTCACTATAGGAGGTAACGAATGGACGGGCCATGGGCATGCTCTTCC
**Z-MGA-OFF-stem D-Rev**	GGAGTCGGGACGGGACGTAGCAAGGCGCAAAG
**Z-MGA-OFF-stem E-Fwd**	GCTAATACGACTCACTATAGCGAGGTAACGAATGGACGGGCCATGGGCATGCTCTTCC
**Z-MGA-OFF-stem E-Rev**	GCGAGTCGGGACGGGACGTAGCAAGGCGCAAAG
**Z-MGA-OFF-stem F-Fwd**	GCTAATACGACTCACTATAGCGAGGTAACGAATGGACCGGGCCATGGGCATGCTCTTCC
**Z-MGA-OFF-stem F-Rev**	GCGAGTCGGGACCGGGACGTAGCAAGGCGCAAAG
**T7 primer**	GCTAATACGACTCACTATA
**2Kt-17-Z-long-Tmp**	GGCTCACCTCGGCTCATCACGCCACTTCGCATGGCTCATCACGCCAGAAGCTGCTGTCCCTATAGTGAGTCGTATTAGC
**2dKt-17-Z-long-Tmp**	GGCTCACCTCGGCTCAGGCCGCCACTTCGCATGGCTCAGGCCGCCAGAAGCTGCTGTCCCTATAGTGAGTCGTATTAGC
**2Kt-17-Z-short-Tmp**	GGGCTCACCTCGGCTCTGACCACTTCGCATGGCTCTGACCAGAAGCTGCTGTCCC
**2Kt-17-Z-short-4-Fwd**	GCTAATACGACTCACTATAGGACGGGCTCACCTCGGCTCTGACC
**2Kt-17-Z-short-4-C-Rev**	GACGGGACAGCAGCTTCTGGTCAG

**2Kt-17-Z-short-5-Fwd**	GCTAATACGACTCACTATAGGAGCGGGCTCACCTCGGCTCTGACC
**2Kt-17-Z-short-5-C-Rev**	GAGCGGGACAGCAGCTTCTGGTCAG
**2Kt-17-Z-short-6-Fwd**	GCTAATACGACTCACTATAGGACACGGGCTCACCTCGGCTCTGACC
**2Kt-17-Z-short-6-C-Rev**	GACACGGGACAGCAGCTTCTGGTCAG
**2Kt-17-Z-short-7-Fwd**	GCTAATACGACTCACTATAGGACACCGGGCTCACCTCGGCTCTGACC
**2Kt-17-Z-short-7-C-Rev**	GACACCGGGACAGCAGCTTCTGGTCAG
**2Kt-17-Z-short-8-Fwd**	GCTAATACGACTCACTATAGGACAACCGGGCTCACCTCGGCTCTGACC
**2Kt-17-Z-short-8-C-Rev**	GACAACCGGGACAGCAGCTTCTGGTCAG
**2U1A-Z-long-Fwd**	GCTAATACGACTCACTATAGGGCCATGGGCATGCTCTTCCGAGACATTGCACCCGGTTCATTCCT CTCGCATGG
**2U1A-Z-long-C-Rev**	GGACGTAGCAAGGCGCACAGAACCCGGGTGCAATGTCTCCATGCGAGAGGAATGAACCGGGTG C
**2dU1A-Z-long-Fwd**	GCTAATACGACTCACTATAGGGCCATGGGCATGCTCTTCCGAGACATTACGGCCGGTTCATTCCT CTCGCATGG
**2dU1A-Z-long-C-Rev**	GGACGTAGCAAGGCGCACAGAACCCGGCCGTAATGTCTCCATGCGAGAGGAATGAACCGGCCG TAATG
**2U1A-Z-short-6-Fwd**	GCTAATACGACTCACTATAGGACACCGGGACGTAGCAAGGCGCACAGAACCCGGAGTCTCCATG CGAGAG
**2U1A-Z-short-6-C-Rev**	GACACGGGCCATGGGCATGCTCTTCCGAGACTCCGGTTCATTCCTCTCGCATGGAGACTCCGGG TTCTG
**1,3,6,9,14Kt-33-Tri-long-Fwd**	GCTAATACGACTCACTATAGGGTAGCAAGAGTGAGGAGCTGTTCAC
**1Kt-33-Tri-long-Rev**	GGGACAGCTCGACCAGGATGAGCACCACGTTCATCATGCCTCTTGTGAACAGCTCCTCACTCTT GC
**3Kt-33-Tri-long-Rev**	GGGGTCATCGATCTCGAACTCGTCTGACTCTG
**6Kt-33-Tri-long-Rev**	GGGGTTCAGCGTGGTCACGAGAGTGGTGC
**9Kt-33-Tri-long-Rev**	GGGAACGTAGCCTTCGGACATGGATGAC
**14Kt-33-Tri-long-Rev**	GGGTTGTGATACAGGATGTTGCCGTCC (= 1,3,6,9,14rcKt-33-Tri-short-Fwd)
**1Kt-33-Tri-short-Fwd**	GCTAATACGACTCACTATAGGGACAGCTCGACCAGGATGAGCACCACGTTCTGACCTCTTGTGAA CAGCTCCTCACTC
**3Kt-33-Tri-short-Fwd**	GCTAATACGACTCACTATAGGGGTCATCGATCTCGAACTCGTCTGAC
**6Kt-33-Tri-short-Fwd**	GCTAATACGACTCACTATAGGGGTTCAGCGTGGTCACGAGAGTGGTG
**9Kt-33-Tri-short-Fwd**	GCTAATACGACTCACTATAGGGAACGTAGCCTTCGGACATGGATGAC
**14Kt-33-Tri-short-Fwd**	GCTAATACGACTCACTATAGGGTTGTGATACAGGATGTTGCCGTC
**1,3,6,9,14-Tri-33-short-Rev**	GGGTAGCAAGAGTGAGGAGCTGTTCAC (= 14rcKt-33-Tri-long-Fwd)
**1rcKt-33-Tri-long-Fwd**	GGGGCATCGAGCTGAAGTACATCGACTTCAATGCGTGATCTGAAGGACGGCAACATCCTGTATCA CAAC
**3rcKt-33-Tri-long-Fwd**	GGGTCGCAACTACAAGACACGTGCAGAGGTG
**6rcKt-33-Tri-long-Fwd**	GGGCAACCACATGAAGCAGCACGACTTCTTC
**9rcKt-33-Tri-long-Fwd**	GGGGCGCACCACTTGCAAGCTGCCAGTGC
**14rcKt-33-Tri-long-Fwd**	GGGTAGCAAGAGTGAGGAGCTGTTCAC(= 1,3,6,9,14-Tri-33-short-Rev)
**1,3,6,9,14rcKt-33-Tri-long-Rev**	GCTAATACGACTCACTATAGGGTTGTGATACAGGATGTTGCCGTCC
**1,3,6,9,14rcKt-33-Tri-short-Fwd**	GGGTTGTGATACAGGATGTTGCCGTCC (= 14Kt-33-Tri-long-Rev)
**1rcKt-33-Tri-short-Rev**	GCTAATACGACTCACTATAGGGGCATCGAGCTGAAGTACATCGACTTCAATGTCACTGAAGGACG GCAACATCCTGTATC
**3rcKt-33-Tri-short-Rev**	GCTAATACGACTCACTATAGGGTCGCAACTACAAGACACGTGCAGAGGTG
**6rcKt-33-Tri-short-Rev**	GCTAATACGACTCACTATAGGGCAACCACATGAAGCAGCACGACTTCTTC
**9rcKt-33-Tri-short-Rev**	GCTAATACGACTCACTATAGGGGCGCACCACTTGCAAGCTGCCAGTGC
**14rcKt-33-Tri-short-Rev**	GCTAATACGACTCACTATAGGGTAGCAAGAGTGAGGAGCTGTTCAC
**14Kt-27,28-Zig-long-Fwd**	GCTAATACGACTCACTATAGGGTAGCAAGAGTGAGGAGTCCAAGAG
**14Kt-27,28-Zig-long-Rev**	GGGTTGTGATACGAGGACGTCCTTCAG (= 14rcKt-27,28-Zig-short-Fwd)
**14Kt-27,28-Zig-short-Fwd**	GCTAATACGACTCACTATAGGGTTGTGATACGAGGACGTCCTTCAG
**14Kt-27,28-Zig-short-Rev**	GGGTAGCAAGAGTGAGGAGTCCAAGAG (= 14rcKt-27,28-Zig-long-Fwd)
**14rcKt-27,28-Zig-long-Fwd**	GGGTAGCAAGAGTGAGGAGTCCAAGAG (= 14Kt-27,28-Zig-short-Rev)
**14rcKt-27,28-Zig-long-Rev**	GCTAATACGACTCACTATAGGGTTGTGATACGAGGACGTCCTTCAG
**14rcKt-27,28-Zig-short-Fwd**	GGGTTGTGATACGAGGACGTCCTTCAG (= 14Kt-27,28-Zig-long-Rev)
**14rcKt-27,28-Zig-short-Rev**	GCTAATACGACTCACTATAGGGTAGCAAGAGTGAGGAGTCCAAGAG

Supplementary Table 2. Template sequences used in the PCRs for RNA nanostructures

**14Kt-33-Tri-long-DNA template (BamHI, XbaI)**	CACGGATCCGGGTAGCAAGAGTGAGGAGCTGTTCACAAGAGGCATGATGAACGTGGTGCTCATC<br>CTGGTCGAGCTGTAAGCGTGATCGATGACACGTAACAGGTCACAAGTTCAGCCGCATGATCAGA<br>GTCAGACGAGTTCGAGATCGATGACATGCATGATGCTTTCAGGACAGCTGACCTGTCAGTTCATG<br>GCGTGATGAGCGCACCACTTGCAAGCTGCCAGTGCAATGCATGATCTCGCACCACTCTCGTGAC<br>CACGCTGAACTGGCGTGATCAACAGCGTGCAGTGCTTCAGTTGCTACCATGCGTGATGACAACC<br>ACATGAAGCAGCACGACTTCTTCCGCATGATGGTAGTCATCCATGTCCGAAGGCTACGTTCAGCA<br>TGATCACCGAGCACACCATCTTCTTCAAGGACGAAGCGTGATGTTCGCAACTACAAGACACGTGC<br>AGAGGTGGGCATGATCCATGTTCGAGTACGACACCCTGGTGAACTAGCATGATGTGCATCGAGCT<br>GAAGTACATCGACTTCAATGCGTGATCTGAAGGACGGCAACATCCTGTATCACAACCCTCTAGAG<br>TC
**14Kt-33-Tri-short-DNA template (BamHI, XbaI)**	CACGGATCCGGGTTGTGATACAGGATGTTGCCGTCCTTCAGTGACATTGAAGTCGATGTACTTCA<br>GCTCGATGCACTGACTAGTTCACCAGGGTGTCGTACTCGAACATGGTGACCCACCTCTGCACGT<br>GTCTTGTAGTTGCGAACTGACTTCGTCCTTGAAGAAGATGGTGTGCTCGGTGTGACTGAACGTAG<br>CCTTCGGACATGGATGACTACCTGACGGAAGAAGTCGTGCTGCTTCATGTGGTTGTCTGACATGG<br>TAGCAACTGAAGCACTGCACGCTGTTGTGACCAGTTCAGCGTGGTCACGAGAGTGGTGCGAGT<br>GACATTGCACTGGCAGCTTGCAAGTGGTGCGCTCTGACCATGAACTGACAGGTCAGCTGTCCTG<br>AAAGCTGACATGTCATCGATCTCGAACTCGTCTGACTCTGTGACGGCTGAACTTGTGACCTGTTA<br>CGTGTCATCGTGACTTACAGCTCGACCAGGATGAGCACCACGTTCTGACCTCTTGTGAACAGCTC<br>CTCACTCTTGCTACCCTCTAGAGTC
**14Kt-27,28-Zig-long-DNA template (BamHI, XbaI)**	CACGGATCCGGGTAGCAAGAGTGAGGAGTCCAAGAGGCATGATGAACGTGGTGCTCATCCTGCT<br>GTAAGCGTGATCGATGAAACAGGTCACAAGTTCAGCCGCATGATCAGAGGAGTTCGAGATCGATG<br>ACATGCATGATGCTTTCAGGACAGCTGACCTGTCATGGCGTGATGAGGCATGCAAGCTGCCAGT<br>GCAATGCATGATCTCGCACCAACCTCGTGACCGAACTGGCGTGATCAACAGCGTGCTTCGTTGC<br>TACCATGCGTGATGAACGTATAGCAGCACGACTTCTTCCGCATGATGGTTCCATTCCGAAGGCTAC<br>GTTCAGCATGATCACCGAGCACACGCATCAAGGACGAAGCGTGATGTTCGCAACTACACGTGCA<br>GAGTGGGCATGATCCATGTTCGACATCCCTGGTGAACTAGCATGATGTGCATCGAGCTGCATCGC<br>TTCAATGCGTGATCTGAAGGACGTCCTCGTATCACAACCCTCTAGAGTC
**14Kt-27,28-Zig-short-DNA template (BamHI, XbaI)**	CACGGATCCGGGTTGTGATACGAGGACGTCCTTCAGTGACATTGAAGCGATGCAGCTCGATGCA<br>CTGACTAGTTCACCAGGGATGTCGAACATGGTGACCCACTCTGCACGTGTAGTTGCGAACTGACT<br>TCGTCCTTGATGCGTGTGCTCGGTGTGACTGAACGTAGCCTTCGGAATGGAACCTGACGGAAGA<br>AGTCGTGCTGCTATACGTTCTGACATGGTAGCAACGAAGCACGCTGTTGTGACCAGTTCGGTCAC<br>GAGGTTGGTGCGAGTGACATTGCACTGGCAGCTTGCATGCCTCTGACCATGACAGGTCAGCTGT<br>CCTGAAAGCTGACATGTCATCGATCTCGAACTCCTCTGTGACGGCTGAACTTGTGACCTGTTTCA<br>TCGTGACTTACAGCAGGATGAGCACCACGTTCTGACCTCTTGGACTCCTCACTCTTGCTACCCTC<br>TAGAGTC

Supplementary Table 3. Primer sequences used in the PCRs for modRNA

**dCasp-8-Fwd (for plasmid construction)**	CACGAATTCTCTATGAAAGATCAAGCACAGAGAGAAG
**Caspase-8-Fwd (for plasmid construction)**	CACGAATTCTCGATTTCCAGAGTTGTCTTTATGCTATTGCTG
**Caspase-8-Rev (for plasmid construction)**	GACCTCGAGGGGAGGGAAGAAGAGCTTCTTCCGTAG
**Lin28A-Fwd (for plasmid construction)**	TATGCGGCCGCTTATGGGCTCCGTGTCCAACCAGCAG
**Lin28A-Rev (for plasmid construction)**	AGAGAATTCTATTCTGTGCCTCCGGGAGCAG
**T7FwdG3C**	CAGTGAATTGTAATACGACTCACTATAGGGC
**GCT87pro_5UTR2**	GCTAATACGACTCACTATAGGTTCCTTAATCGCGGATCC
**CGCT7G2**	CGCTAATACGACTCACTATAGG
**5'-UTR-Tmp**	CAGTGAATTGTAATACGACTCACTATAGGGCGAATTAAGAGAGAAAAGAAGAGTAAGAAGAAATATAAGACACCGGTCGCCACCATG
**5'-UTR-Tmp-T21-5p**	CGACTCACTATAGGTTCCGCGATCGCGGATCCTCAACATCAGTCTGATAAGCTAAGATCACACCGGTCGCCACCATG
**5'-UTR-Tmp-T302a-5p**	CGACTCACTATAGGTTCCGCGATCGCGGATCCAGCAAGTACATCCACGTTTAAGTAGATCCACCGGTCGCCACCATG
**5'-UTR-Rev**	CATGGTGGCGACCGGTGTCTTATATTTCTTCTTACTC
**3'-UTR-Fwd**	TCTAGACCTTCTGCGGGGC
**3'-UTR-Tmp**	TCTAGACCTTCTGCGGGGCTTGCCTTCTGGCCATGCCCTTCTTCTCTCCCTTGCACCTGTACCTCTTGGTCTTTGAATAAAGCCTGAGTAGG
**3'-UTR-Rev**	TTTTTTTTTTTTTTTTTTTTCCTACTCAGGCTTTATTCAAAGACCAAG
**Rev120A**	TTTTTTTTTTTTTTTTTTTTTTTTTTTTTTTTTTTTTTTTTTTTTTTTTTTTTTTTTTTTTTTTTTTTTTTTTTTTTTTTTTTTTTTTTTTTTTTTTTTTTTTTTTTTTTTTTTTTTTTTCCTACTCAGGCTTTATTCA
**L7Ae-ORF-Fwd**	CACCGGTCGCCACCATGTACGTGAGATTTGAGGTTCCTG
**MS2CP-ORF-Fwd**	CACCGGTCGCCACCATGGGATCCGCTTC
**TAP-Rev**	GCCCCGCAGAAGGTCTAGACTATCACTCGAGATGCATATGAGATC
**myc-His6-ORF-Rev**	GCCCCGCAGAAGGTCTAGATCAATGGTGATGGTGATGATGACCG
**myc-EGFP-ORF-Fwd**	CACCGGTCGCCACCATGGAACAAAAACTCATCTCAGAAGAGGATCTGCCGCGCGGCAGCCATATGCGGCCGCTTGTGAGCAAGGGCGAGGAGCTG
**dCasp-8-ORF-Fwd**	CACCGGTCGCCACCATGTATGAAAGATCAAGCACAGAGAGAAG
**Caspase-8-ORF-Fwd**	CACCGGTCGCCACCATGGATTTCCAGAGTTGTCTTTATGC
**Caspase-8-ORF-Rev**	GCCCCGCAGAAGGTCTAGATCATTAGGGAGGGAAGAAGAGCTTC
**Lin28A-ORF-Fwd**	CACCGGTCGCCACCATGGGCTCCGTGTCCAAC
**Lin28A-ORF-Rev**	GCCCCGCAGAAGGTCTAGATTCAATTCTGTGCCTCCGGGAG

Supplementary Table 4. Primer sets used in the PCRs for RNA nanostructures

PCR product	Forward primer	Reverse primer	Template
2Kt-33-Tri-long	2Kt-33-Tri-long-Fwd	2Kt-33-Tri-long-Rev	None
2Kt-33-Tri-long+C	2Kt-33-Tri-long-Fwd	2Kt-33-Tri-long+C-Rev	None
2dKt-33-Tri-long	2dKt-33-Tri-long-Fwd	2dKt-33-Tri-long-Rev	None
2Kt-33-Tri-short	2Kt-33-Tri-short-Fwd	2Kt-33-Tri-short-Rev	None
2Kt-28-Z-long	2Kt-28-Z-long-Fwd	2Kt-28-Z-long-Rev	None
2dKt-28-Z-long	2dKt-28-Z-long-Fwd	2dKt-28-Z-long-Rev	None
2Kt-28-Z-short	2Kt-28-Z-short-Fwd	2Kt-28-Z-short-Rev	None
2Kt-28-Z-long-C	2Kt-28-Z-long-Fwd	2Kt-28-Z-long-C-Rev	None
2dKt-28-Z-long-C	2dKt-28-Z-long-Fwd	2dKt-28-Z-long-C-Rev	None
2Kt-28-Z-short-5-C	2Kt-28-Z-short-6'-Fwd	2Kt-28-Z-short-5-C-Rev	None
2Kt-28-Z-short-6'-C	2Kt-28-Z-short-6'-Fwd	2Kt-28-Z-short-6'-C-Rev	None
2Kt-28-Z-short-6-C	2Kt-28-Z-short-7-Fwd	2Kt-28-Z-short-6-C-Rev	None
2Kt-28-Z-short-7-C	2Kt-28-Z-short-7-Fwd	2Kt-28-Z-short-7-C-Rev	None
Tri-MGA-ON-short-1st-PCR	Tri-MGA-ON-short-1st-Fwd	2Kt-33-Tri-long-Rev	None
Z-MGA-OFF-short-1st-PCR	Z-MGA-OFF-short-1st-Fwd	2Kt-28-Z-short-Rev	None
Tri-MGA-ON-short-U0	short-MGA-original-U0-Fwd	short-MGA-original-U0-Rev	Tri-MGA-ON-short-1st-PCR
Tri-MGA-ON-short-U2	short-MGA-original-U2-Fwd	short-MGA-original-U2-Rev	Tri-MGA-ON-short-1st-PCR
Tri-MGA-ON-short-U4	short-MGA-original-U4-Fwd	short-MGA-original-U4-Rev	Tri-MGA-ON-short-1st-PCR
Z-MGA-OFF-short-U0	short-MGA-original-U0-Fwd	short-MGA-original-U0-Rev	Z-MGA-OFF-short-1st-PCR
Z-MGA-OFF-short-U2	short-MGA-original-U2-Fwd	short-MGA-original-U2-Rev	Z-MGA-OFF-short-1st-PCR
Z-MGA-OFF-short-U4	short-MGA-original-U4-Fwd	short-MGA-original-U4-Rev	Z-MGA-OFF-short-1st-PCR
Tri-MGA-ON-short-stem A	Tri-MGA-ON-stem A-U0-Fwd	Tri-MGA-ON-stem A-U0-Rev	Tri-MGA-ON-short-1st-PCR
Tri-MGA-ON-short-stem B	Tri-MGA-ON-stem B-U0-Fwd	Tri-MGA-ON-stem B-U0-Rev	Tri-MGA-ON-short-1st-PCR
Tri-MGA-ON-short-stem C	Tri-MGA-ON-stem C-U0-Fwd	Tri-MGA-ON-stem C-U0-Rev	Tri-MGA-ON-short-1st-PCR
Z-MGA-OFF-short-stem D	Z-MGA-OFF-stem D-U0-Fwd	Z-MGA-OFF-stem D-U0-Rev	Z-MGA-OFF-short-1st-PCR
Z-MGA-OFF-short-stem E	Z-MGA-OFF-stem E-U0-Fwd	Z-MGA-OFF-stem E-U0-Rev	Z-MGA-OFF-short-1st-PCR
Z-MGA-OFF-short-stem F	Z-MGA-OFF-stem F-U0-Fwd	Z-MGA-OFF-stem F-U0-Rev	Z-MGA-OFF-short-1st-PCR
2Kt-17-Z-long	T7 primer	2Kt-17-Z-long-Tmp	None
2dKt-17-Z-long	T7 primer	2dKt-17-Z-long-Tmp	None
2Kt-17-Z-short-4-C	2Kt-17-Z-short-4-Fwd	2Kt-17-Z-short-4-C-Rev	2Kt-17-Z-short-Tmp
2Kt-17-Z-short-5-C	2Kt-17-Z-short-5-Fwd	2Kt-17-Z-short-5-C-Rev	2Kt-17-Z-short-Tmp
2Kt-17-Z-short-6-C	2Kt-17-Z-short-6-Fwd	2Kt-17-Z-short-6-C-Rev	2Kt-17-Z-short-Tmp
2Kt-17-Z-short-7-C	2Kt-17-Z-short-7-Fwd	2Kt-17-Z-short-7-C-Rev	2Kt-17-Z-short-Tmp
2Kt-17-Z-short-8-C	2Kt-17-Z-short-8-Fwd	2Kt-17-Z-short-8-C-Rev	2Kt-17-Z-short-Tmp
2U1A-Z-long-C	2U1A-Z-long-Fwd	2U1A-Z-long-C-Rev	None
2dU1A-Z-long-C	2dU1A-Z-long-Fwd	2dU1A-Z-long-C-Rev	None
2U1A-Z-short-6-C	2U1A-Z-short-6-Fwd	2U1A-Z-short-6-C-Rev	None
1Kt-33-Tri-long	1,3,6,9,14Kt-33-Tri-long-Fwd	1Kt-33-Tri-long-Rev	None
3Kt-33-Tri-long	1,3,6,9,14Kt-33-Tri-long-Fwd	3Kt-33-Tri-long-Rev	pcDNA4/TO-14Kt-33-Tri-long
6Kt-33-Tri-long	1,3,6,9,14Kt-33-Tri-long-Fwd	6Kt-33-Tri-long-Rev	pcDNA4/TO-14Kt-33-Tri-long
9Kt-33-Tri-long	1,3,6,9,14Kt-33-Tri-long-Fwd	9Kt-33-Tri-long-Rev	pcDNA4/TO-14Kt-33-Tri-long
14Kt-33-Tri-long	1,3,6,9,14Kt-33-Tri-long-Fwd	14Kt-33-Tri-long-Rev	pcDNA4/TO-14Kt-33-Tri-long
1Kt-33-Tri-short	1Kt-33-Tri-short-Fwd	1,3,6,9,14Kt-33-Tri-short-Rev	None
3Kt-33-Tri-short	3Kt-33-Tri-short-Fwd	1,3,6,9,14Kt-33-Tri-short-Rev	pcDNA4/TO-14Kt-33-Tri-short
6Kt-33-Tri-short	6Kt-33-Tri-short-Fwd	1,3,6,9,14Kt-33-Tri-short-Rev	pcDNA4/TO-14Kt-33-Tri-short
9Kt-33-Tri-short	9Kt-33-Tri-short-Fwd	1,3,6,9,14Kt-33-Tri-short-Rev	pcDNA4/TO-14Kt-33-Tri-short
14Kt-33-Tri-short	14Kt-33-Tri-short-Fwd	1,3,6,9,14Kt-33-Tri-short-Rev	pcDNA4/TO-14Kt-33-Tri-short
1rcKt-33-Tri-long	1rcKt-33-Tri-long-Fwd	1,3,6,9,14rcKt-33-Tri-long-Rev	None
3rcKt-33-Tri-long	3rcKt-33-Tri-long-Fwd	1,3,6,9,14rcKt-33-Tri-long-Rev	pcDNA4/TO-14Kt-33-Tri-long
6rcKt-33-Tri-long	6rcKt-33-Tri-long-Fwd	1,3,6,9,14rcKt-33-Tri-long-Rev	pcDNA4/TO-14Kt-33-Tri-long
9rcKt-33-Tri-long	9rcKt-33-Tri-long-Fwd	1,3,6,9,14rcKt-33-Tri-long-Rev	pcDNA4/TO-14Kt-33-Tri-long
14rcKt-33-Tri-long	14rcKt-33-Tri-long-Fwd	1,3,6,9,14rcKt-33-Tri-long-Rev	pcDNA4/TO-14Kt-33-Tri-long
1rcKt-33-Tri-short	1,3,6,9,14rcKt-33-Tri-short-Fwd	1rcKt-33-Tri-short-Rev	None
3rcKt-33-Tri-short	1,3,6,9,14rcKt-33-Tri-short-Fwd	3rcKt-33-Tri-short-Rev	pcDNA4/TO-14Kt-33-Tri-short
6rcKt-33-Tri-short	1,3,6,9,14rcKt-33-Tri-short-Fwd	6rcKt-33-Tri-short-Rev	pcDNA4/TO-14Kt-33-Tri-short
9rcKt-33-Tri-short	1,3,6,9,14rcKt-33-Tri-short-Fwd	9rcKt-33-Tri-short-Rev	pcDNA4/TO-14Kt-33-Tri-short
14rcKt-33-Tri-short	1,3,6,9,14rcKt-33-Tri-short-Fwd	14rcKt-33-Tri-short-Rev	pcDNA4/TO-14Kt-33-Tri-short
14Kt-27,28-Zig-long	14Kt-27,28-Zig-long-Fwd	14Kt-27,28-Zig-long-Rev	pcDNA4/TO-14Kt-27,28-Zig-long
14Kt-27,28-Zig-short	14Kt-27,28-Zig-short-Fwd	14Kt-27,28-Zig-short-Rev	pcDNA4/TO-14Kt-27,28-Zig-short
14rcKt-27,28-Zig-long	14rcKt-27,28-Zig-long-Fwd	14rcKt-27,28-Zig-long-Rev	pcDNA4/TO-14Kt-27,28-Zig-long
14rcKt-27,28-Zig-short	14rcKt-27,28-Zig-short-Fwd	14rcKt-27,28-Zig-short-Rev	pcDNA4/TO-14Kt-27,28-Zig-short
9pre-let7d-33-long	1,3,6,9,14Kt-33-Tri-long-Fwd	9Kt-33-Tri-long-Rev	p9pre-let7d-33-long

Supplementary Table 5. Primer sets used in the PCRs for modRNA

PCR product	Forward primer	Reverse primer	Template
5'-UTR PCR	T7FwdG3C	5'-UTR-Rev	5'-UTR-Tmp
5'-UTR-T21-5p PCR	GCT87pro_5UTR2	5'-UTR-Rev	5'-UTR-Tmp-T21-5p
5'-UTR-T302a-5p PCR	GCT87pro_5UTR2	5'-UTR-Rev	5'-UTR-Tmp-T302a-5p
3'-UTR PCR	3'-UTR-Fwd	3'-UTR-Rev	3'-UTR-Tmp
L7Ae-coding region PCR	L7Ae-ORF-Fwd	TAP-Rev	pTAP-L7Ae
L7Ae-myc-coding region PCR	L7Ae-ORF-Fwd	myc-His6-ORF-Rev	pcDNA3.1-L7Ae-myc-His6
EGFP-myc-coding region PCR	myc-EGFP-ORF-Fwd	3'-UTR-Rev	pUC19-EGFPfull
MS2CP-coding region PCR	MS2CP-ORF-Fwd	TAP-Rev	pCTp-MS2CP
dCasp-8 coding region PCR	dCasp-8-ORF-Fwd	Caspase-8-ORF-Rev	pBS-mFLICE
L7Ae-dCasp-8-coding region PCR	L7Ae-ORF-Fwd	Caspase-8-ORF-Rev	pcDNA3.1-L7Ae-dCasp-8-myc-His6
L7Ae-dCasp-8-myc-coding region PCR	L7Ae-ORF-Fwd	myc-His6-ORF-Rev	pcDNA3.1-L7Ae-dCasp-8-myc-His6
L7Ae-dCasp-8-CS-coding region PCR	L7Ae-ORF-Fwd	Caspase-8-ORF-Rev	pcDNA3.1-L7Ae-dCasp-8-CS-myc-His6
Caspase-8-coding region PCR	Caspase-8-ORF-Fwd	Caspase-8-ORF-Rev	pBS-mFLICE
L7Ae-caspase-8-coding region PCR	L7Ae-ORF-Fwd	Caspase-8-ORF-Rev	pcDNA3.1-L7Ae-caspase-8-myc-His6
L7Ae-caspase-8-CS-coding region PCR	L7Ae-ORF-Fwd	Caspase-8-ORF-Rev	pcDNA3.1-L7Ae-caspase-8-CS-myc-His6
Lin28A coding region PCR	Lin28A-ORF-Fwd	Lin28A-ORF-Rev	pTOPO-hLin28
Lin28A-dCasp-8 coding region PCR	Lin28A-ORF-Fwd	Caspase-8-ORF-Rev	pcDNA3.1-Lin28A-dCasp-8-myc-His6

Supplementary Table 6. Transfection tables for all experiments in this study

Figure 3d, Supplementary Figure 21

	MS2CP + 2dKt-17-Z-7	MS2CP + 2Kt-17-Z-7	L7Ae + 2dKt-17-Z-7	L7Ae + 2Kt-17-Z-7
MS2CP	500 ng	500 ng		
L7Ae			500 ng	500 ng
2Kt-17-Z-7		8 pmol		8 pmol
2dKt-17-Z-7	8 pmol		8 pmol	
Plate	12 well	12 well	12 well	12 well
HeLa cell	50000 cells	50000 cells	50000 cells	50000 cells

Figure 4b-d

	1Kt	3Kt	6Kt	9Kt	14Kt
1Kt-33-Tri	0.1 pmol				
3Kt-33-Tri		0.1 pmol			
6Kt-33-Tri			0.1 pmol		
9Kt-33-Tri				0.1 pmol	
14Kt-33-Tri					0.1 pmol
L7Ae-dCasp-8	10 ng	10 ng	10 ng	10 ng	10 ng
Plate	24 well	24 well	24 well	24 well	24 well
HeLa cell	50000 cells	50000 cells	50000 cells	50000 cells	50000 cells

	1rcKt	3rcKt	6rcKt	9rcKt	14rcKt
1rcKt-33-Tri	0.1 pmol				
3rcKt-33-Tri		0.1 pmol			
6rcKt-33-Tri			0.1 pmol		
9rcKt-33-Tri				0.1 pmol	
14rcKt-33-Tri					0.1 pmol
L7Ae-dCasp-8	10 ng	10 ng	10 ng	10 ng	10 ng
Plate	24 well	24 well	24 well	24 well	24 well
HeLa cell	50000 cells	50000 cells	50000 cells	50000 cells	50000 cells

Figure 4e

	No protein			L7Ae		
	No RNA	14Kt-33-Tri	14rcKt-33-Tri	No RNA	14Kt-33-Tri	14rcKt-33-Tri
14Kt-33-Tri		0.1 pmol			0.1 pmol	
14rcKt-33-Tri			0.1 pmol			0.1 pmol
L7Ae				10 ng	10 ng	10 ng
Plate	24 well	24 well	24 well	24 well	24 well	24 well
HeLa cell	50000 cells	50000 cells	50000 cells	50000 cells	50000 cells	50000 cells

	dCasp-8			L7Ae-dCasp-8		
	No RNA	14Kt-33-Tri	14rcKt-33-Tri	No RNA	14Kt-33-Tri	14rcKt-33-Tri
14Kt-33-Tri		0.1 pmol			0.1 pmol	
14rcKt-33-Tri			0.1 pmol			0.1 pmol
dCasp-8	10 ng	10 ng	10 ng			
L7Ae-dCasp-8				10 ng	10 ng	10 ng
Plate	24 well	24 well	24 well	24 well	24 well	24 well
HeLa cell	50000 cells	50000 cells	50000 cells	50000 cells	50000 cells	50000 cells

	L7Ae-dCasp-8-CS			Casp-8		
	No RNA	14Kt-33-Tri	14rcKt-33-Tri	No RNA	14Kt-33-Tri	14rcKt-33-Tri
14Kt-33-Tri		0.1 pmol			0.1 pmol	
14rcKt-33-Tri			0.1 pmol			0.1 pmol
L7Ae-dCasp-8-CS	10 ng	10 ng	10 ng			
Casp-8				10 ng	10 ng	10 ng
Plate	24 well	24 well	24 well	24 well	24 well	24 well
HeLa cell	50000 cells	50000 cells	50000 cells	50000 cells	50000 cells	50000 cells

	L7Ae-Casp-8			L7Ae-Casp-8-CS		
	No RNA	14Kt-33-Tri	14rcKt-33-Tri	No RNA	14Kt-33-Tri	14rcKt-33-Tri
14Kt-33-Tri		0.1 pmol			0.1 pmol	
14rcKt-33-Tri			0.1 pmol			0.1 pmol
L7Ae-Casp-8	10 ng	10 ng	10 ng			
L7Ae-Casp-8-CS				10 ng	10 ng	10 ng
Plate	24 well	24 well	24 well	24 well	24 well	24 well
HeLa cell	50000 cells	50000 cells	50000 cells	50000 cells	50000 cells	50000 cells

Figure 4f

	L7Ae-dCasp-8	
	14Kt-33-Tri	14rcKt-33-Tri
14Kt-33-Tri	0.4 pmol	
14rcKt-33-Tri		0.4 pmol
L7Ae-dCasp-8	40 ng	40 ng
Plate	6 well	6 well
HeLa cell	200000 cells	200000 cells

Figure 5b

	9pre-let7d-33	9pre-let7d-33	9pre-let7d-33	9pre-let7d-33	9pre-let7d-33
	–	L7Ae-dCasp-8	Lin28A-dCasp-8	Lin28A-dCasp-8	Lin28A-dCasp-8
	–	–	–	Lin28A	MS2CP
9pre-let7d-33	0.2 pmol	0.2 pmol	0.2 pmol	0.2 pmol	0.2 pmol
L7Ae-dCasp-8		10 ng			
Lin28A-dCasp-8			40 ng	40 ng	40 ng
Lin28A				40 ng	
MS2CP					40 ng
Plate	24 well	24 well	24 well	24 well	24 well
HeLa cell	50000 cells	50000 cells	50000 cells	50000 cells	50000 cells

	9Kt-33-Tri	9Kt-33-Tri	9Kt-33-Tri	9Kt-33-Tri	9Kt-33-Tri
	–	L7Ae-dCasp-8	Lin28A-dCasp-8	Lin28A-dCasp-8	Lin28A-dCasp-8
	–	–	–	Lin28A	MS2CP
9Kt-33-Tri	0.2 pmol	0.2 pmol	0.2 pmol	0.2 pmol	0.2 pmol
L7Ae-dCasp-8		10 ng			
Lin28A-dCasp-8			40 ng	40 ng	40 ng
Lin28A				40 ng	
MS2CP					40 ng
Plate	24 well	24 well	24 well	24 well	24 well
HeLa cell	50000 cells	50000 cells	50000 cells	50000 cells	50000 cells

Figure 5c

	9pre-let7d-33	9pre-let7d-33	9pre-let7d-33
	–	L7Ae-dCasp-8	Lin28A-dCasp-8
9pre-let7d-33	0.2 pmol	0.2 pmol	0.2 pmol
L7Ae-dCasp-8		10 ng	
Lin28A-dCasp-8			40 ng
Plate	24 well	24 well	24 well
HeLa-Lin28A cell	50000 cells	50000 cells	50000 cells

	9Kt-33-Tri	9Kt-33-Tri	9Kt-33-Tri
	–	L7Ae-dCasp-8	Lin28A-dCasp-8
9Kt-33-Tri	0.2 pmol	0.2 pmol	0.2 pmol
L7Ae-dCasp-8		10 ng	
Lin28A-dCasp-8			40 ng
Plate	24 well	24 well	24 well
HeLa-Lin28A cell	50000 cells	50000 cells	50000 cells

Figure 5d

	9pre-let7d-33	9pre-let7d-33	9pre-let7d-33
	–	L7Ae-dCasp-8	Lin28A-dCasp-8
9pre-let7d-33	0.4 pmol	0.4 pmol	0.4 pmol
L7Ae-dCasp-8		30 ng	
Lin28A-dCasp-8			40 ng
Plate	24 well	24 well	24 well
hiPSC	50000 cells	50000 cells	50000 cells

	9Kt-33-Tri	9Kt-33-Tri	9Kt-33-Tri
	–	L7Ae-dCasp-8	Lin28A-dCasp-8
9Kt-33-Tri	0.4 pmol	0.4 pmol	0.4 pmol
L7Ae-dCasp-8		30 ng	
Lin28A-dCasp-8			40 ng
Plate	24 well	24 well	24 well
hiPSC	50000 cells	50000 cells	50000 cells

Figure 6b

	14Kt-33-Tri		14rcKt-33-Tri	
	(–) mimic	(+) mimic	(–) mimic	(+) mimic
14Kt-33-Tri	0.1 pmol	0.1 pmol		
14rcKt-33-Tri			0.1 pmol	0.1 pmol
miR-302 inhibitor				
miR-302-mimic		0.5 pmol		0.5 pmol
miR-302-L7Ae-dCasp-8	10 ng	10 ng	10 ng	10 ng
Plate	24 well	24 well	24 well	24 well
HeLa cell	50000 cells	50000 cells	50000 cells	50000 cells

	14Kt-33-Tri		14rcKt-33-Tri	
	(–) inhibitor	(+) inhibitor	(–) inhibitor	(+) inhibitor
14Kt-33-Tri	0.2 pmol	0.2 pmol		
14rcKt-33-Tri			0.2 pmol	0.2 pmol
miR-302 inhibitor		2 pmol		2 pmol
miR-302-mimic				
miR-302-L7Ae-dCasp-8	30 ng	30 ng	30 ng	30 ng
Plate	24 well	24 well	24 well	24 well
hiPSC	50000 cells	50000 cells	50000 cells	50000 cells

Figure 6c

	14Kt-33-Tri		14rcKt-33-Tri	
	(–) inhibitor	(+) inhibitor	(–) inhibitor	(+) inhibitor
14Kt-33-Tri	0.1 pmol	0.1 pmol		
14rcKt-33-Tri			0.1 pmol	0.1 pmol
miR-21 inhibitor		2 pmol		2 pmol
miR-21-mimic				
miR-21-L7Ae-dCasp-8	10 ng	10 ng	10 ng	10 ng
Plate	24 well	24 well	24 well	24 well
HeLa cell	50000 cells	50000 cells	50000 cells	50000 cells

	14Kt-33-Tri		14rcKt-33-Tri	
	(–) mimic	(+) mimic	(–) mimic	(+) mimic
14Kt-33-Tri	0.2 pmol	0.2 pmol		
14rcKt-33-Tri			0.2 pmol	0.2 pmol
miR-21 inhibitor				
miR-21-mimic		2 pmol		2 pmol
miR-21-L7Ae-dCasp-8	30 ng	30 ng	30 ng	30 ng
Plate	24 well	24 well	24 well	24 well
hiPSC	50000 cells	50000 cells	50000 cells	50000 cells

Supplementary Figure 26c

	0 ng modRNA	10 ng modRNA	20 ng modRNA
L7Ae-myc or L7Ae-dCasp-8-myc	0 ng	10 ng	20 ng
MS2CP	250 ng	240 ng	230 ng
Kt-EGFP	100 ng	100 ng	100 ng
Plate	24 well	24 well	24 well
HeLa cell	50000 cells	50000 cells	50000 cells

	50 ng modRNA	100 ng modRNA	250 ng modRNA
L7Ae-myc or L7Ae-dCasp-8-myc	50 ng	100 ng	250 ng
MS2CP	200 ng	150 ng	0 ng
Kt-EGFP	100 ng	100 ng	100 ng
Plate	24 well	24 well	24 well
HeLa cell	50000 cells	50000 cells	50000 cells

Supplementary Figure 33

	14Kt-33-Tri		14Kt-27,28-Zig	
	Kt	rcKt	Kt	rcKt
14Kt-33-Tri	0.1 pmol			
14rcKt-33-Tri		0.1 pmol		
14Kt-27,28-Zig			0.1 pmol	
14rcKt-27,28-Zig				0.1 pmol
L7Ae-dCasp-8	10 ng	10 ng	10 ng	10 ng
Plate	24 well	24 well	24 well	24 well
HeLa cell	50000 cells	50000 cells	50000 cells	50000 cells

Supplementary Note 1

Optimization of biMGA-conjugated RNA nanodevices for the efficient ON/OFF switching of RNA activity

To optimize the efficiency of the activation (ON) or repression (OFF) of the biMGA activity in the absence and presence of L7Ae, we first examined the effect of the U linker lengths between the RNA device (2Kt-33-Tri or 2Kt-28-Z) and biMGA (Supplementary Figs. 7–9). Increasing the length of the U linker led to a decrease in the efficiency of switching biMGA activity. Thus, RNA devices without a U linker (U0) were chosen for further engineering. To increase the efficiency of the ON or OFF switching of biMGA activity, we next investigated the effect of the stem sequences located in biMGA (Supplementary Fig. 10–12). For the activation of biMGA using 2Kt-33-Tri, we tested three stems (stems A, B, and C; Supplementary Fig. 10) that were less stable than the original stem. We expected that the less stable stems could disrupt the association of biMGA on 2Kt-33-Tri in the absence of L7Ae. In fact, RNA devices that contained stems A-C increased the change in biMGA activity before and after the addition of L7Ae [FI (+ L7Ae)/FI (– L7Ae)] compared with that of the original RNA device (Supplementary Fig. 12a). The greatest change was observed for stem B (2.5-fold). To improve the OFF switch device using 2Kt-28-Z, we tested three other stems (stems D, E, and F; Supplementary Fig. 11) that were more stable than the original stem. These stems should increase the association of biMGA on 2Kt-28-Z in the absence of L7Ae. As expected, the engineered 2Kt-28-Z with stems D-F showed higher biMGA activity than that of the original stem in the absence of L7Ae (Supplementary Fig. 12b). The lowest change [FI (+ L7Ae)/ FI (– L7Ae)] was observed for the original stem and stem D. The RNA device with stem D showed a higher fluorescence intensity in the absence of L7Ae. Mutants of the optimized RNA devices, dKt-Tri-MGA-ON and dKt-Z-MGA-OFF, showed smaller and larger [FI (+ L7Ae)/FI (– L7Ae)] values, respectively than the corresponding RNA devices (Supplementary Fig. 13). We constructed Tri-MGA-ON and Z-MGA-OFF devices (Fig. 2h–j) by employing 2Kt-33-Tri with stem B and 2Kt-28-Z with stem D, respectively.
